# Regiodivergent α- and β‑Functionalization of Saturated *N*‑Heterocycles by Photocatalytic Oxidation

**DOI:** 10.1021/jacs.5c06177

**Published:** 2025-06-30

**Authors:** Jonas W. Rackl, Alexander F. Müller, Antonia Profyllidou, Helma Wennemers

**Affiliations:** Laboratorium für Organische Chemie, 27219 ETH Zürich, D-CHAB, Zürich 8093, Switzerland

## Abstract

Synthetic methods that provide access to two different types of products via a central intermediate are highly valuable but difficult to establish. Here, we present a photocatalytic, regiodivergent method for the functionalization of saturated *N*-heterocycles at either the α- or the β-position. A *t*-butyl carbamate (Boc)-stabilized iminium ion serves as the key intermediate en route to either α-hydroxylation or β-elimination, depending on the choice of base. The operationally simple procedures use a readily available flavin-based catalyst and reagents, aqueous media and do not require metals. Combined with facile downstream derivatization, the regiodivergent reaction gives rapid access to a large set of functionalized piperidines, molecules that are highly sought-after for the synthesis of pharmaceuticals and agrochemicals.

## Graphical abstract

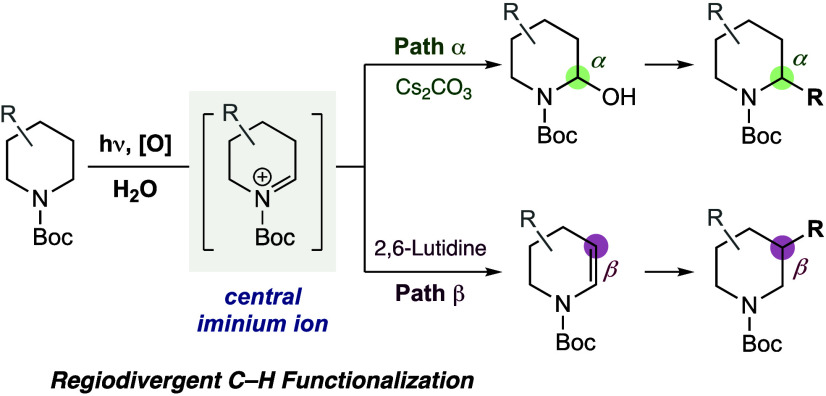

Saturated *N*-heterocycles are ubiquitous in pharmaceuticals, with more than two-thirds of the current FDA-approved drugs containing at least one saturated *N*-heterocycle (Scheme 1A).^1–3^ Among them, piperidine is the most frequent.^1–3^ Straightforward and robust synthetic methods to access functionalized piperidines and other saturated *N*-heterocycles are therefore important.^4–7^ The α-functionalization of saturated *N*-heterocycles has been achieved through directed metalation,^8–11^ electrochemistry,^12–16^ and transition metal catalysis,^17–21^ in some examples, combined with photochemistry.^6,22–31^ Functionalization in the β-position is more challenging, since the β-C–H bond is not activated and is more remote from the nitrogen center. Clever metalation approaches and radical chemistry enabled functionalization with either aryl, alkyl, or fluorine moieties at C^β^,^15,32–39^ in some cases with concomitant α-functionalization.^15,40^ Most of these α- and β-functionalization methods involve two or more steps and use *N*-heterocycles bearing a specific directing or stabilizing group that can be cumbersome to remove. Notable exceptions are photochemical methods introduced by Seidel and Nicewicz that use an easily removable protecting group and an acridinium photocatalyst in combination with a metal-based Lewis acid under inert reaction conditions for either α- or β-functionalization.^26,38^

**Scheme 1:**
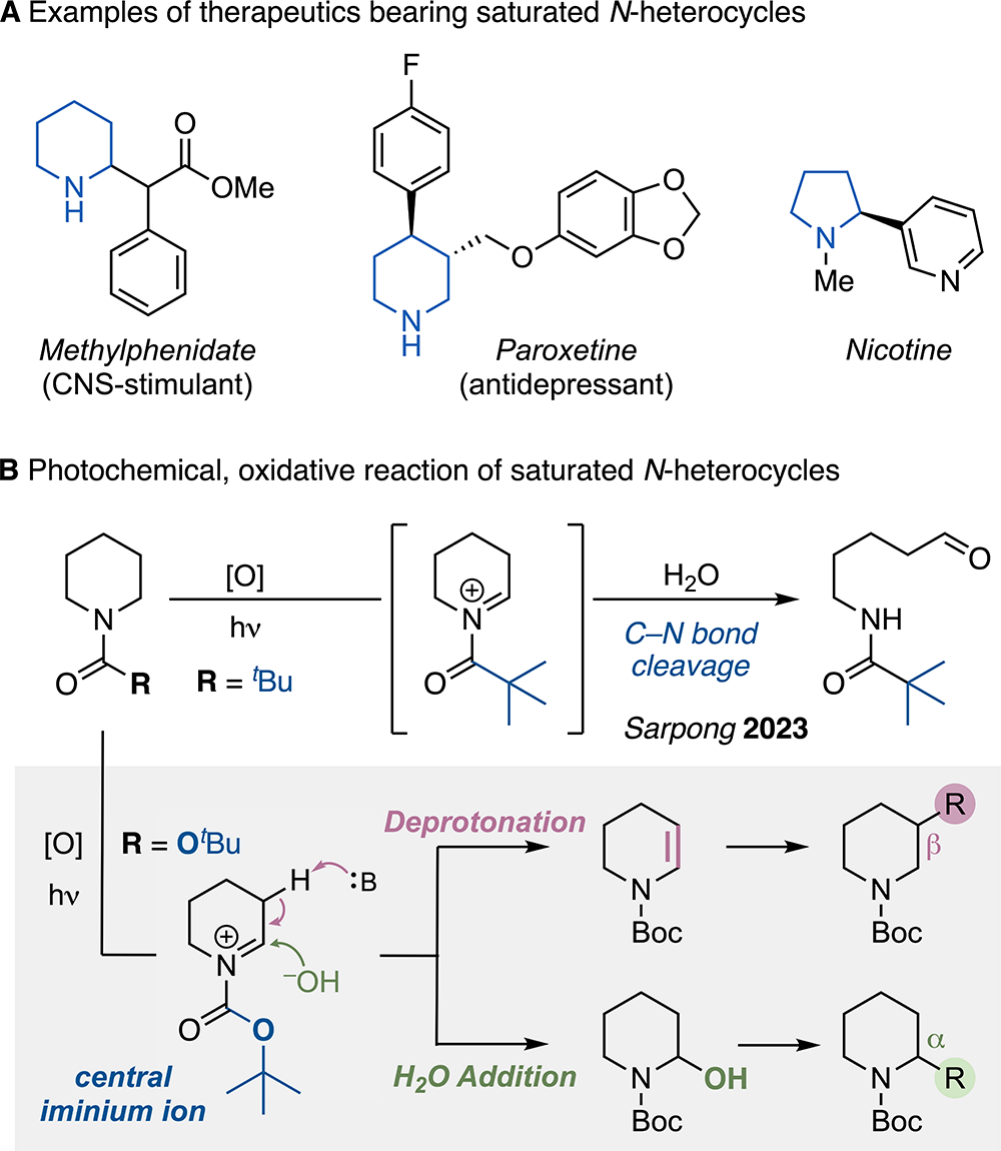
Examples of Saturated *N*-Heterocyclic Amines and Envisioned Regiodivergent Functionalization

A general method for the controlled regioselective functionalization at either the α- or the β-position, starting from the same building block, would be a powerful tool to streamline the synthesis of substituted saturated *N*-heterocycles. Such a concept would be particularly attractive if it proceeded under ambient conditions and allowed the introduction of functional handles for facile further derivatizations of the heterocyclic scaffold. We envisioned that a stabilized iminium ion arising from the photochemical oxidation of an *N*-heterocycle should allow, through the choice of an appropriate base, targeting of either the α- or β-position (Scheme 1B). Herein, we present the realization of such a regiodivergent photocatalytic oxidation to α- and β-functionalized saturated piperidines and other *N*-heterocycles.

Recently, Sarpong developed an elegant photocatalytic method for the deconstructive diversification of *N*-pivaloyl (Piv)-protected saturated *N*-heterocycles.^41^ In the presence of the photocatalyst riboflavin tetraacetate (RFTA),^42^ light, and stoichiometric amounts of potassium persulfate, the pivaloylated cyclic amines oxidize to the corresponding iminium cation, and hydrolysis provides, via a labile hemiaminal, linear aldehydes (Scheme 1B, top).

We reasoned that variation of the functional group at N from an amide to a more electron-rich carbamate should provide an iminium ion sufficiently stable to serve as a central intermediate for derivatization into either α- or β-substituted *N*-heterocycles (Scheme 1B, bottom). Nucleophilic trapping of the iminium cation by water should result in a stable hemiaminal, whereas an appropriate base should facilitate β-elimination to an enecarbamate. Both the hemiaminal and the enecarbamate are versatile building blocks for a broad range of further downstream derivatizations.

We used Boc-protected piperidine as a model heterocycle to explore the envisioned photocatalytic oxidation. The corresponding hemiaminal is a stable, isolatable compound. Under the reaction conditions established by Sarpong,^41^ *N*-Boc-piperidine did, however, not react to the corresponding hemiaminal. Yet, more than 50% of *N*-Boc-piperidine was consumed, as determined by NMR spectroscopy of the crude reaction mixture, indicating oxidation products. In addition, the pH of the mixture dropped from pH 7 to 1, further supporting the envisioned photochemical oxidation through hydrogen (H^+^ and e^–^) abstraction. The ensuing acidic aqueous environment disfavors the trapping of the intermediate iminium ion. Building on these observations, we envisioned that increasing the pH of the reaction mixture should allow for nucleophilic trapping of the intermediate iminium ion by water. Alternatively, an appropriate base should allow for abstraction of the proton at C^β^ (Scheme 1B, bottom).

We, therefore, performed the photochemical reaction in the presence of different bases (Table 1). The addition of Cs_2_CO_3_ kept the pH at 7 or above and converted 49% of *N*-Boc-piperidine to the respective hemiaminal as determined by ^1^H NMR spectroscopy (Table 1, entry 2). In contrast, 2,6-lutidine, a sterically demanding base, provided the enecarbamate (Table 1, entry 3). Other inorganic and organic bases provided either of the two products in significantly lower amounts or not at all (Table 1, entries 4–7). These observations suggest that the p*K*_aH_ and the nature of the base are key for gearing the reaction into either nucleophilic trapping by water at C^α^ or proton abstraction at C^β^.

**Table 1:**
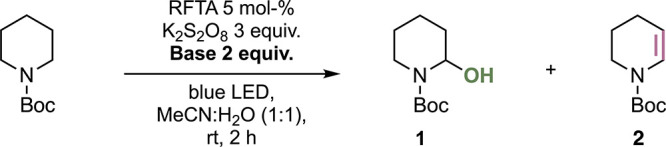
Testing of Different Bases for the Hydroxylation and Desaturation of *N*-Boc Piperidine^a^

entry	base	**1** [%]^b^ ^,^ ^c^	**2** [%]^b^ ^,^ ^c^
1	none	n.d.	n.d.
2	Cs_2_CO_3_	49	n.d.
3	2,6-Lutidine	n.d.	42
4	NaTFA	n.d.	n.d.
5	K_2_HPO_4_	n.d.	27
6	iPr_2_NEt	n.d.	*traces*
7	DMAP	n.d.	*traces*

a Reaction scale 0.3 mmol.

b n.d. = not detected.

c Determined by ^1^H NMR spectroscopy with Me_4_Si as internal standard.

Following these initial studies, both the hydroxylation and desaturation protocols were further optimized by variations of the photocatalyst, oxidant, base, and other common reaction parameters such as the reaction time and stoichiometry (Tables S1–6). These efforts yielded optimal conditions for hydroxylation at C^α^ and deprotonation at C^β^. Aside from the base, the optimal reaction conditions differ with respect to the equivalents of the photocatalyst and the oxidant as well as the reaction time (Scheme 2). For both, a mixture of acetonitrile and water (1:1) and a concentration of the *N*-heterocycle of 0.02 M proved to be optimal.

**Scheme 2:**
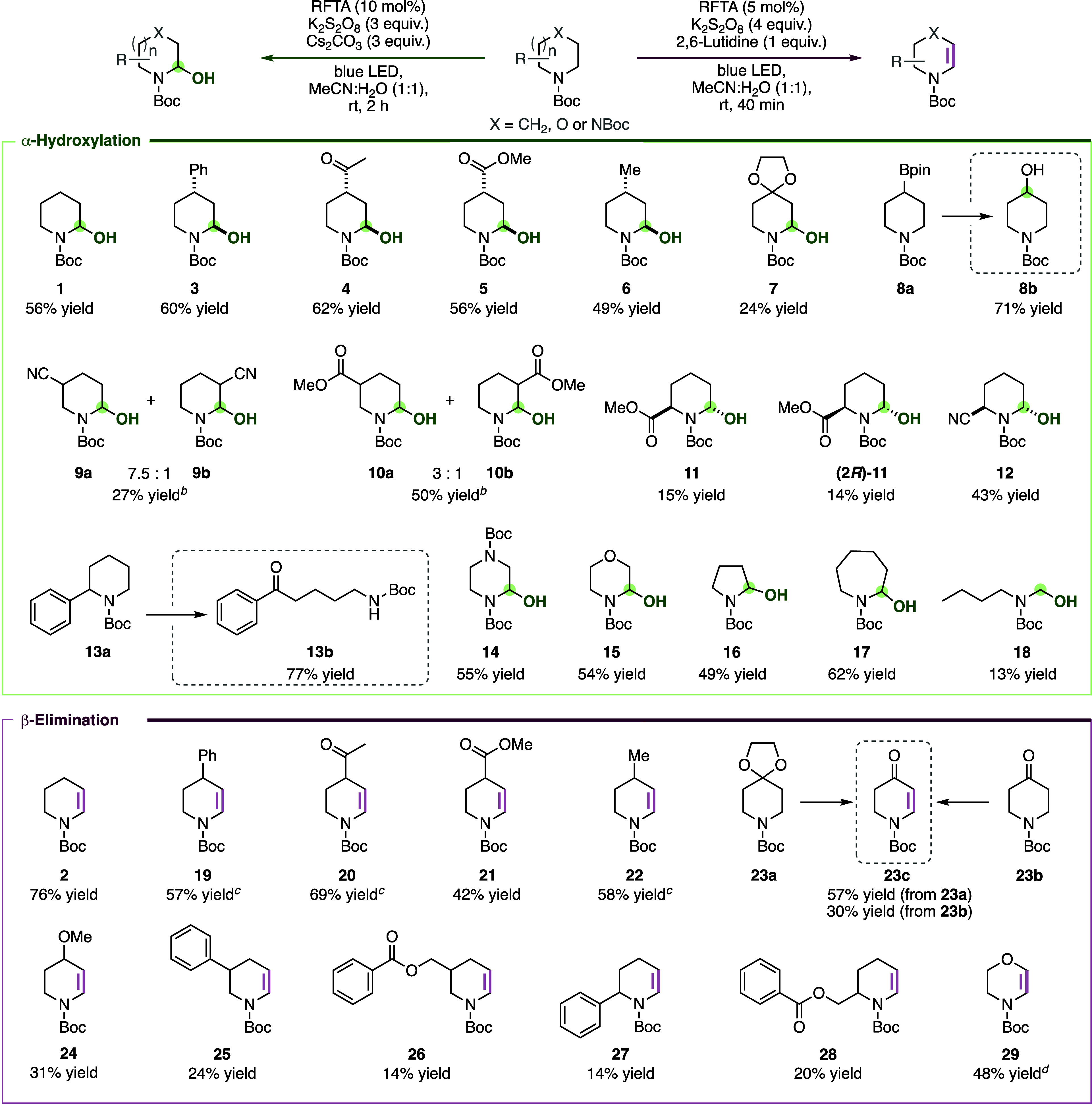
Scope of α- and β-Functionalization of Saturated *N*-Heterocycles^a^

a Yields refer to isolated compounds unless noted otherwise. Reaction scale 0.3 mmol. Note, the carbamate undergoes *trans*/*cis* isomerization.
b The relative configuration could not be determined due to overlapping signals.
c Determined by ^1^H NMR spectroscopy using C_2_H_2_Cl_4_ as internal standard.
d Obtained in one pot by α-hydroxylation followed by pTsOH 5 mol %, MgSO_4_, EtOAc, 70 °C, 12 h.

With optimal reaction conditions in hand, we investigated the scope of the α- and β-functionalization. First, we explored the scope of the hydroxylation at C^α^ (Scheme 2, top). Piperidines with substituents at C^γ^ reacted to the respective hemiaminals (**3**–**7**) with yields of 24–62%.^43^ These experiments showed that alkyl, aryl, ester, acetal, and keto groups are tolerated. NMR spectroscopic analyses imply the predominant formation of hemiaminals with a relative *trans* configuration and the OH group in an axial position, likely due to a stereoelectronic effect.^44^ Subjecting 4-Bpin-substituted *N*-Boc piperidine (**8a**) to the established reaction conditions afforded the corresponding hydroxylated product at the boron-substituted position (**8b**) in 71% yield, likely via a mechanism analogous to the Brown hydroboration–oxidation reaction.^45^ Hydroxylation of β-substituted piperidines resulted in mixtures of regioisomers (**9a/b** and **10a/b**), preferentially at the sterically more accessible α-position. These experiments showed that even photochemically active groups, such as a nitrile moiety, are tolerated under the mild reaction conditions. α-Substituted piperidines provided the respective *trans*-configured hemiaminals (**11**, **12**) regioselectively at the unsubstituted α-carbon in yields of 14–43%. Hydroxylation of the enantiomerically pure methylester of (*R*)-pipecolic acid yielded (*R*,*R*)-configured hemiaminal (2*R*)-**11**, indicating that the stereochemistry at C^α^ remains intact during the photochemical reaction. Interestingly, 2-phenylpiperidine (**13a**) underwent an unexpected ring opening by C–N bond cleavage, followed by benzylic oxidation to the acyclic benzoyl carbamate **13b**. In contrast to the prior ring cleavage by Sarpong with *N*-pivaloyl piperidines that takes place at the unsubstituted C^α^–N,^41^ this ring opening occurred at the site bearing a substituent at C^α^.

We also explored the α-hydroxylation with analogs of piperidine. The photocatalytic oxidation of *N*-Boc-morpholine and bis-Boc-protected piperazine, substrates that could undergo either double or competing hydroxylation at the carbon adjacent to oxygen, yielded exclusively the monofunctionalized products **14** and **15** in yields of ∼55%.^43^ Reactions with pyrrolidine and azepane showed that the reaction is not limited to 6-membered cyclic amines. The respective hemiaminals **16** and **17** were isolated in yields of 49% and 62%, respectively. Also noteworthy, the α-hydroxylation converted acyclic *N*-Boc-protected butyl-methylamine to the less substituted hemiaminal **18**, albeit in a low yield of 13%. Furthermore, at a 10-fold larger scale (3 mmol), hemiaminal **1** was obtained in essentially the same yield (55%).

Next, we explored the scope of the β-elimination (Scheme 2, bottom). We started with piperidines bearing different substituents at C^γ^. Enecarbamates with aryl, keto, ester, alkyl, and ether moieties (**19**–**22**, **24**) were obtained in yields of 31–69%. Boc-protected 4-piperidone and also its acetal-protected derivative yielded enecarbamate **23c**. This scope is remarkable since many of the functional groups are prone to undergo photochemical reactions. β-Substituted piperidines dehydrogenated selectively at the less substituted α,β-site (**25** and **26**). Similarly, α-substituted piperidines underwent highly regioselective desaturation at the less substituted α,β-site to enecarbamates **27** and **28**. Enecarbamate **29**, derived from *N*-Boc-morpholine, was obtained by photochemical oxidative α-hydroxylation followed by water elimination in one pot, without detectable double-desaturation. Attempts to form enecarbamates from *N*-Boc-azepane and *N*-Boc-pyrrolidine were so far unsuccessful, indicating a limitation of the reaction scope.^46^

We envisioned the α-hydroxylated cyclic amines and enecarbamates as versatile building blocks for downstream derivatization (Scheme 3, top). Indeed, α-functionalization of **1**, used as a model hemiaminal, by Mukaiyama-type alkylation with a silyl-enol ether resulted in α-alkylated piperidine **30** in 83% yield. Lewis acid activation of the hemiaminal and subsequent nucleophilic trapping allowed C–O bond formation to geraniol derivative **31** in 49% yield. Furylation (**32**) proceeded quantitatively, highlighting that aryl substituents can also be introduced at C^α^. Also α-cyanation (**33**) through activation with BF_3_·OEt_2_ and reaction with TMSCN proceeded in a good yield of 72%. The reaction with allyl-TMS provided, instead of the α-allylated piperidine, bicycle **34** in 78% yield. The reaction likely proceeds via intramolecular nucleophilic cyclization involving the Boc protecting group.^47^ The reaction sequence of α-hydroxylation followed by dehydration through the addition of a catalytic amount of pTsOH in the presence of MgSO_4_ provides an alternative two-step/one-pot route to enecarbamate **2**. The examples show the synthetic versatility of the method for the direct functionalization of piperidines at C^α^ by C–O or C–C bond formation to introduce alkoxy, alkyl, and aryl substituents.

**Scheme 3:**
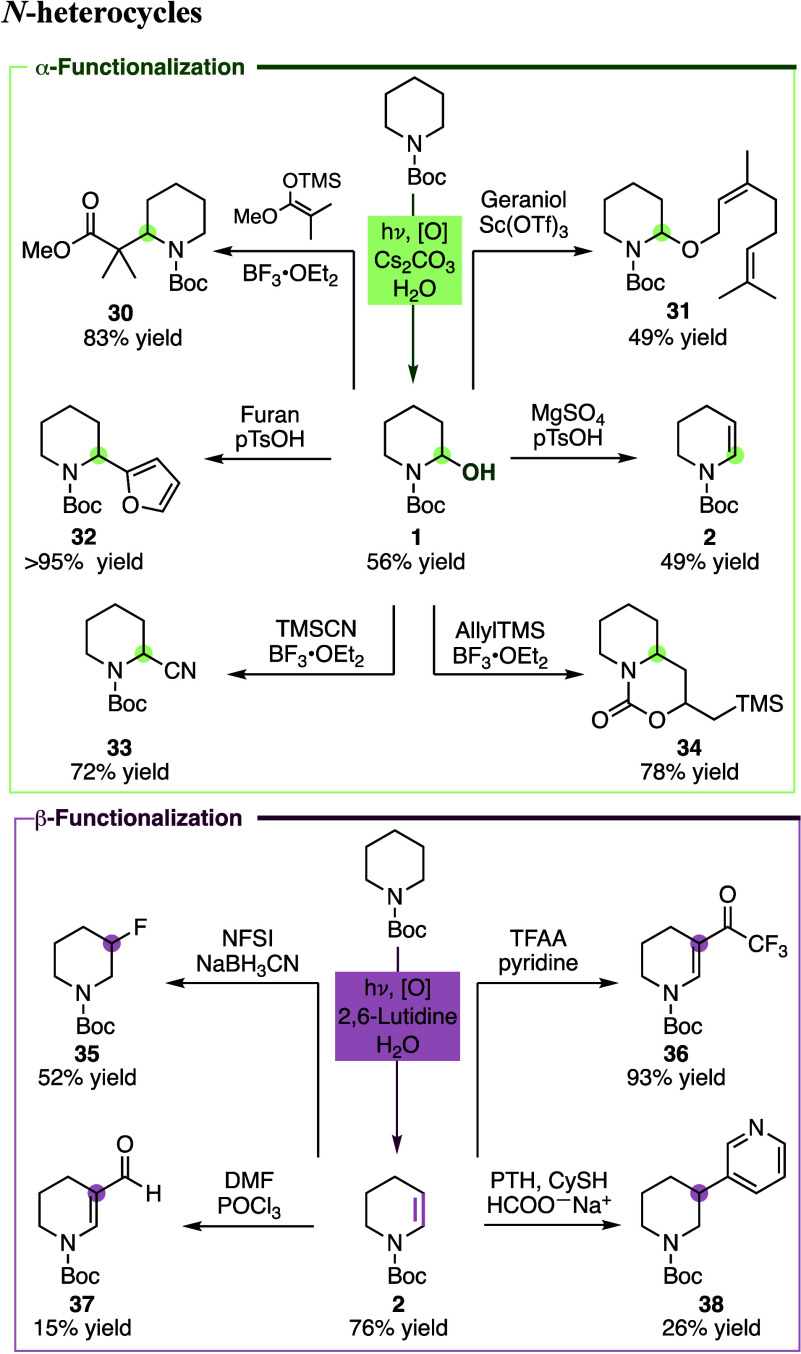
Derivatization of α- and β-Functionalized *N*-Heterocycles

The derivatization of enecarbamates at C^β^ through C–N and C–O bond formation has been introduced before.^38,48,49^ We probed whether Boc-protected enecarbamates can undergo fluorination and C–C bond formation (Scheme 3, bottom). The electrophilic fluorination of enecarbamate **2** by *N*-fluorobenzenesulfonimide (NFSI) and sodium cyanoborohydride (NaBH_3_CN) provided the β-fluorinated piperidine **35** in 52% yield.^50^ Friedel–Crafts acylation with trifluoroacetic acid anhydride (TFAA) and pyridine^51^ gave access to the β-acylated piperidine **36** in 93% yield. β-Formylated piperidine **37** was prepared by Vilsmeier–Haack reaction and photochemical, radical heteroarylation^52^ yielded the C^β^-substituted pyridine derivative **38**.

Finally, we explored the value of our methodology for the α- and β-functionalization of saturated *N*-heterocycles in peptides (Scheme 4). These more complex substrates have become more and more valuable for the development of therapeutics,^53^ with several biologically active peptides featuring pipecolic acid.^54^

**Scheme 4:**
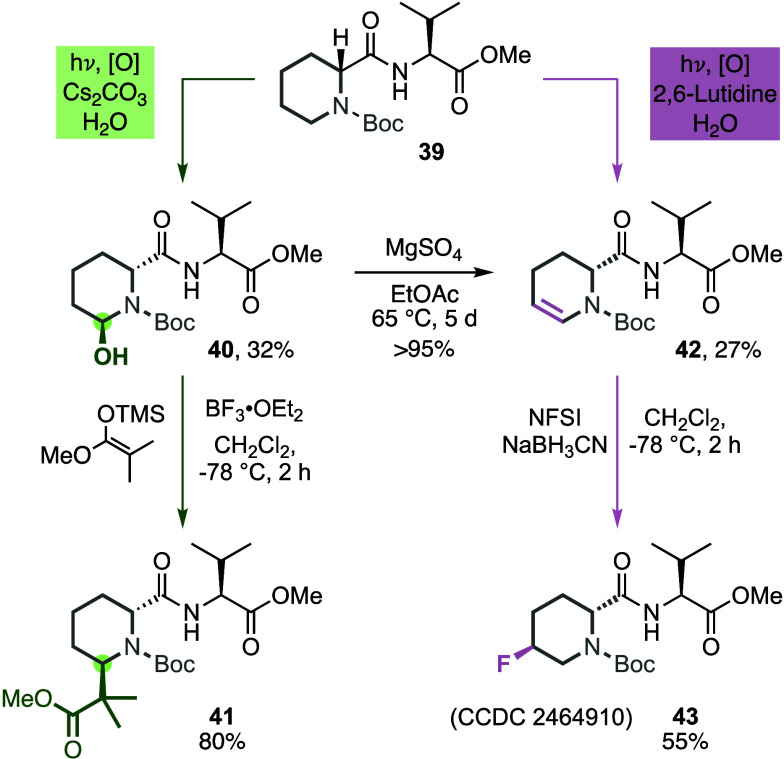
Derivatization of a Pipecolic Acid-containing Peptide at the α- and β-Position

The α-hydroxylation of model peptide **39** proceeded regioselectively with a yield of 32% (**40**). Further reaction with the Mukaiyama silyl-enol ether yielded α-C–C-functionalized peptide **41** in 80% yield. The desaturation protocol was also compatible with peptide **39** and provided the desaturated derivative **42** regioselectively in 27% yield. Alternatively, enecarbamate **42** is accessible by dehydration of hemiaminal **40** (>95% yield). Furthermore, electrophilic fluorination with NFSI and NaBH_3_CN yielded fluorinated peptide **43**. These results highlight the broad synthetic utility of our photochemical oxidative α- and β-functionalization of saturated *N*-heterocycles.

In conclusion, we developed a photocatalytic oxidative platform for the selective direct functionalization of saturated *N*-heterocycles at either the α- or the β-position. Key to control over the reaction pathway is the initial formation of a stabilized iminium ion and an appropriate base to form either the respective hemiaminal for functionalization at C^α^ or the enecarbamate for functionalization at C^β^. Piperidines bearing different substituents, including ester, CN, and carbonyl groups, as well as analogs (morpholine, piperazine, azepane, and pyrrolidine) reacted readily to the desired products. Combined with downstream derivatization, this approach enables direct access to a broad range of piperidines with alkyl, aryl, acyl, F, CN, and alkoxy groups at either the α- or β-position. The method uses a readily available organic photocatalyst and oxidant in an aqueous environment, tolerates air, and does not require metals. Thus, this operationally simple photocatalytic oxidation enables rapid access to a large portion of chemical space starting from readily available saturated *N*-heterocycles. We, therefore, envision that our results will be of practical utility and inspire the further development of straightforward derivatization methods of saturated (hetero)­cyclic scaffolds.

## Supplementary Material



## Notes

Experimental details on the reactions including the analytical data of the presented compounds (PDF)

## ±

Department of Chemistry, Massachusetts Institute of Technology, Cambridge, Massachusetts 02139, United States of America

## ABBREVIATIONS

RFTA: Riboflavin tetra acetate
Piv: pivaloyl
Boc: *tert*-butyloxycarbonyl
PTH: 10-phenylphenothiazine
CySH: cyclohexanethiol
NFSI: *N*-fluorobenzenesulfonimide
TFAA: trifluoroacetic acid anhydride
