# Supplementary material for: Regiodivergent α- and β‑Functionalization of Saturated N‑Heterocycles by Photocatalytic Oxidation
Source: J Am Chem Soc. 2025 Jun 30;147(27):23381–6. doi: 10.1021/jacs.5c06177 (PMC12257516; doi:10.1021/jacs.5c06177)
Supplement: Supplementary file 1 [file ja5c06177_si_001.pdf]

Supporting Information

Regiodivergent α- and β-Functionalization of Saturated *N*-Heterocycles by Photocatalytic Oxidation

Jonas W. Rackl,‡ Alexander F. Müller,‡ Antonia Profyllidou, Helma Wennemers*

Laboratorium für Organische Chemie, ETH Zürich
Vladimir-Prelog-Weg 3, 8093 Zurich, Switzerland
*helma.wennemers@org.chem.ethz.ch

Contents

1. General Information ... 3

  1.1 Reagents, Materials and Solvents ... 3

  1.2 Experimental Procedures ... 3

  1.3 Analytical Instrumentation ... 3

  1.4 Photochemical Setup ... 4

2. Optimization of Reaction Conditions & Control Experiments ... 5

  2.1 Optimization of the α-Hydroxylation ... 5

  2.2 Optimization of the β-Elimination ... 6

3. Catalyst and Starting Materials ... 8

  3.1 Synthesis of the Photocatalyst Riboflavin Tetraacetate (RFTA) ... 8

  3.2 General Procedure for the Synthesis of Non-Commercially Available Piperidines ... 9

  3.3 Analytical Data ... 10

4. Substrate Scope ... 11

  4.1 Hemiaminals ... 11

    4.1.1 General Procedure ... 11

    4.1.2 Analytical Data ... 11

  4.2 Enecarbamates ... 19

    4.2.1 General Procedure ... 19

    4.2.2 Analytical Data ... 19

5. Product Derivatization ... 24

  5.1 Derivatization of Hemiaminals ... 24

  5.2 Derivatization of Enecarbamates ... 27

6. NMR Spectra of Non-Commercial Starting Materials ... 37

7. NMR Spectra of Products ... 39

  7.1 Hemiaminals ... 39

  7.2 Enecarbamates ... 57

8. NMR Spectra of Derivatives of 1 and 2 ... 63

  8.1 Hemiaminal Derivatives ... 63

  8.2 Enecarbamate Derivatives ... 67

9. References ... 69

1. General Information

1.1 Reagents, Materials and Solvents

Reagents and materials were of the highest commercially available grade and used without further purification unless stated otherwise. Except for compounds **SI-1**, **SI-2**, and **39** that were synthesized following protocols described in the subsequent sections, all starting materials were purchased and used directly without further purification.

1.2 Experimental Procedures

Reactions were monitored by ^{1}H-NMR spectroscopy and/or thin layer chromatography (TLC) using Merck silica gel 60 F_{254} glass plates. Visualization of the compounds on TLC was achieved under UV light (254 nm) irradiation and subsequent $KMnO_4$ staining accompanied by gentle heating. Automated flash column chromatography was performed using a Teledyne Isco CombiFlash MPLC system with prepacked silica columns. Solvents for extractions were technical grade and solvents for chromatography were HPLC grade or of technical quality and distilled before use. Preparative thin layer chromatography was performed on Merck silica gel 60 F_{254} glass plates.

1.3 Analytical Instrumentation

Gas chromatography (GC) was carried out on a Shimadzu GC-2025 Series GC-FID instrument with H_2 as carrier gas. An OPTIMA® 5 MS Accent column (0.25 µm, 30 m, 0.25 mm ID) from Macherey-Nagel was used for analysis (heat-temperature curve: starting at 50°C, hold for 2 min, heat to 320 °C with a rate of 30 °C/min, hold for 9 min; total time 20 min).

NMR spectra were recorded on a Bruker Avance 400 (400 MHz/101 MHz), a Bruker Avance 500 (500 MHz/126 MHz), a Bruker Ascend 400 (400 MHz/101 MHz) and a Bruker Ultrashield 400 (400 MHz/101 MHz), typically at 20-25°C. Chemical shifts (δ) of ^{1}H and ^{13}C spectra are reported in parts per million (ppm) relative to the residual solvent signal of $CHCl_3$ (δ 7.26 for ^{1}H NMR, δ 77.16 for ^{13}C NMR in $CDCl_3$) and DMSO (δ 2.50 for ^{1}H NMR in $(CD_3)_2SO$). ^{1}H and ^{13}C NMR spectroscopy data are reported as follows: chemical shift (δ ppm), multiplicity (br = broad, s = singlet, d = doublet, t = triplet, q = quartet, m = multiplet), coupling constant (Hz), integration. Structural elucidation was aided by an additional acquisition of various 2D spectra (^{1}H-^{1}H COSY, ^{1}H-^{13}C HSQC, ^{1}H-^{13}C HMBC).

High-resolution mass spectra were measured by the Molecular and Biomolecular Analysis Service (MoBiAS) in the Laboratory of Organic Chemistry at ETH Zürich on the following instruments: Thermo Scientific QExactive GC Orbitrap (high-resolution EI) and Bruker maXis - ESI-QTOF (high-resolution ESI) spectrometers.

High-performance liquid chromatography (HPLC) analyses were performed on an analytical Ultimate 3000 HPLC system with a diode array detector and chiral stationary phase columns from Daicel (IG). Sample for HPLC analysis was prepared by dissolving the sample in n-hexane/i-PrOH (4:1) and filtering through an Acrodisc® CR 4 mm syringe filter with a 0.45 µm PTFE membrane. The separation conditions are reported below.

1.4 Photochemical Setup

Unless otherwise stated, all photochemical reactions were carried out in the open-source *ETHos* photoreactor.[1] The reactions were stirred and irradiated by commercially available Kessil lamps (Kessil TunaBlue A 160WE, 40 W, wavelength: 380 – 500 nm). The temperature inside the photoreactor was kept at rt by a temperature-sensor controlled fan.

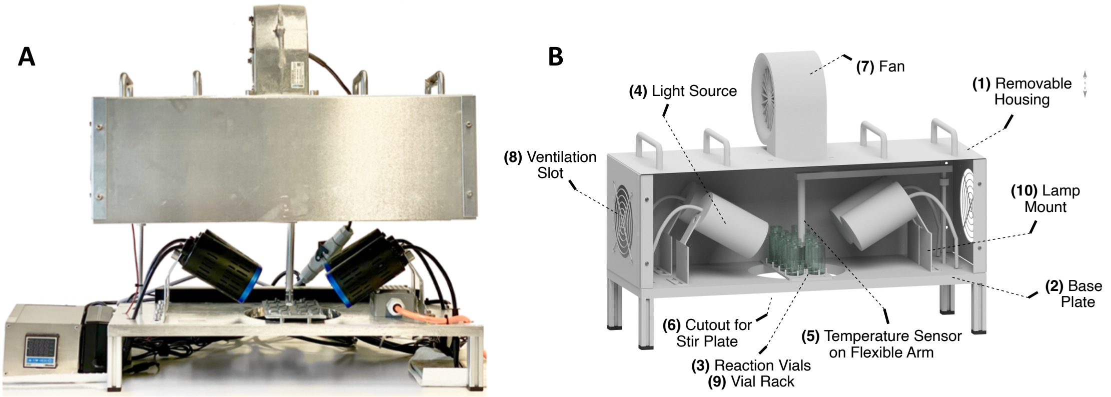

Figure S1. A) Photograph of the photoreactor with the outer housing lifted to view the internal setup. B) CAD representation of the photoreactor with the front panel removed to visualize the reactor’s internal components. The removable housing (**1**), with an external fan (**7**) and ventilation slots (**8**), is mounted on a base plate (**2**). The circular cutout (**6**) in the base plate accommodates a magnetic stirring unit. Light sources (**4**), attached to mounts (**10**) at opposite sides, establish the central irradiation area, which houses the vial rack (**9**) and the temperature sensor (**5**).

For the photochemical reaction at larger scale (3 mmol in batch), a round bottom flask (250 mL) instead of a vial was used.

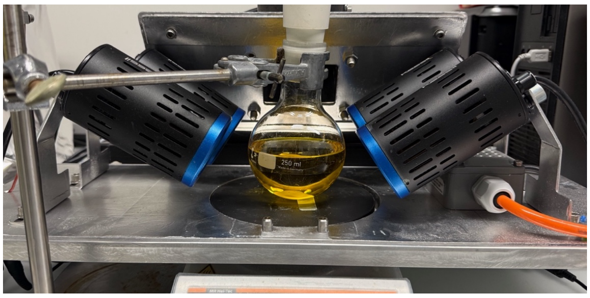

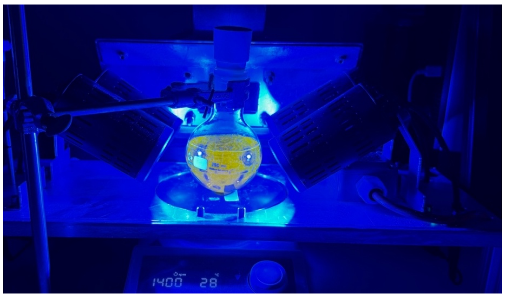

2. Optimization of Reaction Conditions & Control Experiments

2.1 Optimization of the α-Hydroxylation

Amount of Cs_2CO_3

An 8-dram vial equipped with a stirring bar was charged with Boc-protected piperidine (1.0 equiv., 0.3 mmol, 55.6 mg), $K_2S_2O_8$ (3.0 equiv., 0.9 mmol, 243 mg), Cs_2CO_3 (x equiv.), MeCN:H_2O (1:1, 15 mL) and RFTA (5 mol%, 8.2 mg). The mixture was subsequently sonicated until no solid were visible. The solution was irradiated for 2 h under stirring. Then, brine was added (5 mL) and the mixture was extracted with EtOAc (3x5 mL). The combined organic layers were dried over Na_2SO_4, filtered and concentrated under a stream of N_2.

Table S1: Effect of the amount of Cs_2CO_3 on the α-hydroxylation of *N*-Boc piperidine

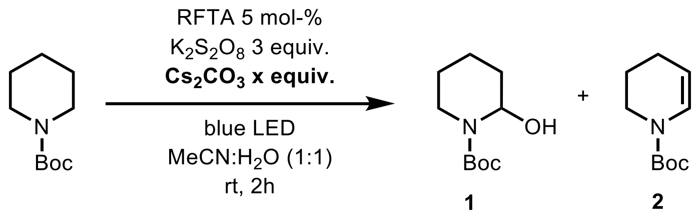

Entry	Cs_2CO_3 [equiv.]	1 [%][a]	2 [%][a]
1	1	9	19
2	2	46	0
3	3	44	0
4	4	43	0

[a] Determined by ^{1}H-NMR spectroscopy with Me_4Si as internal standard.

Catalyst Loading

An 8-dram vial equipped with a stirring bar was charged with Boc-protected piperidine (1.0 equiv., 0.3 mmol, 55.6 mg), $K_2S_2O_8$ (3.0 equiv., 0.9 mmol, 243 mg), Cs_2CO_3 (3 equiv.), MeCN:H_2O (1:1, 15 mL) and RFTA (5 mol%, 8.2 mg) and the mixture was subsequently sonicated until no solid was visible.

Entries 1 & 2 The solution was irradiated for 2 h under stirring. Brine was then added (5 mL) and the mixture was extracted with EtOAc (3x5 mL). The combined organic layers were dried over Na_2SO_4, filtered and concentrated under a stream of N_2.

Entries 3 & 4 The solution was irradiated for 2 h under stirring. Afterwards, more RFTA (5 mol%, 8.2 mg) was added, and irradiation was continued for 1 h (entry 3) or 2 h (entry 4). Subsequently brine (5 mL) was added and the mixture was extracted with EtOAc (3x5 mL). The combined organic layers were dried over Na_2SO_4, filtered and concentrated under a stream of N_2.

Table S2: Effect of the catalyst loading on the α-hydroxylation of *N*-Boc piperidine

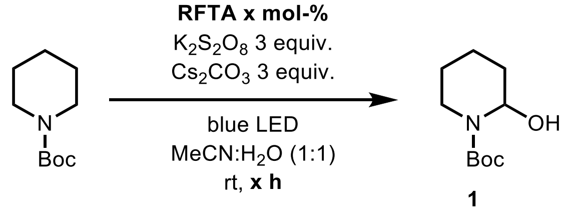

1

Entry	RFTA [mol-%]	time [h]	1[%][a]
1	5	2	44
2	5	3	< 44
3	10	2	67
4	10	3	61

[a] Determined by ^{1}H-NMR spectroscopy with Me_4Si as internal standard.

2.2 Optimization of the β-Elimination

General Procedure

Boc-protected piperidine (1.0 equiv., 0.3 mmol, 55.6 mg), $K_2S_2O_8$ (x equiv.) 2,6-lutidine (x equiv.), MeCN:H_2O (15 mL total volume) and RFTA were placed in an 8-dram vial equipped with a stirring bar. The mixture was sonicated until no solid was visible. The solution was irradiated for 40 min while stirring. Then, brine (5 mL) was added, and the mixture was extracted with EtOAc (3x5 mL). The combined organic layers were dried over $MgSO_4$, filtered, and concentrated under a stream of N_2.

Ratio of H_2O and MeCN

Table S3: Effect of the solvent ratio on the desaturation of *N*-Boc piperidine

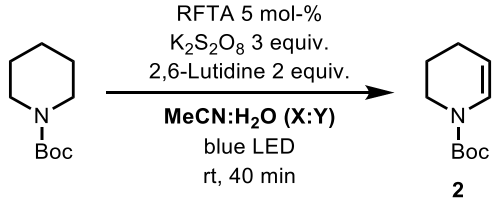

2

Entry	H_2O:MeCN	2 [%][a]
1	25:75	traces
2	50:50	65
3	75:25	57
4	90:10	32

[a] Conversion determined by GC-FID analysis.

Amount of Oxidant $K_2S_2O_8$

Table S4: Effect of the oxidant amount on the desaturation of *N*-Boc piperidine

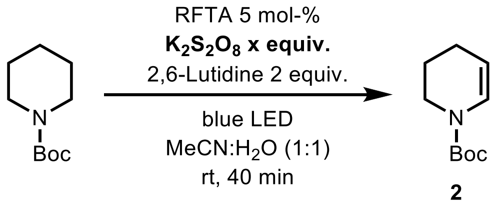

2

Entry	$K_2S_2O_8$	2 [%][a]
1	1	traces
2	3	65
3	4	70
4	8	71

[a] Conversion determined by GC-FID analysis.

Catalyst Loading

Table S5: Effect of the catalyst loading on the desaturation of *N*-Boc piperidine

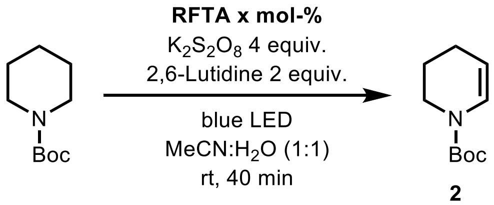

2

Entry	RFTA [mol-%]	2 [%][a]
1	2	34
2	5	70
3	10	57

[a] Conversion determined by GC-FID analysis.

Amount of 2,6-Lutidine

Table S6: Effect of the amount of 2,6-lutidine on the desaturation of *N*-Boc piperidine

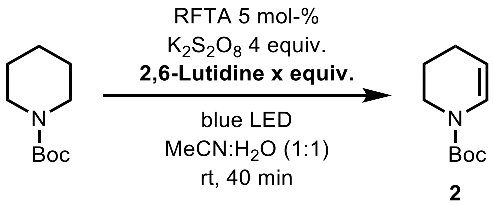

2

Entry	2,6-Lutidine [equiv.]	2 [%][a]
1	1	76
2	2	70
3	4	67
4	6	56

[a] Conversion determined by GC-FID analysis.

Concentration

Table S7: Effect of the concentration on the desaturation of *N*-Boc piperidine.

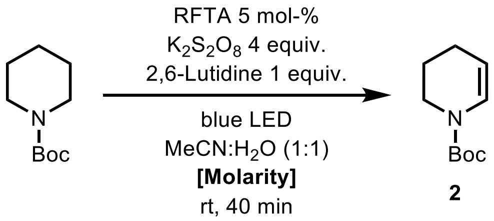

Entry	Concentration [M]	2 [%][a]
1	0.02	76
2	0.04	66
3	0.08	13

[a] Conversion determined by GC-FID analysis.

3. Catalyst and Starting Materials

3.1 Synthesis of the Photocatalyst Riboflavin Tetraacetate (RFTA)

(2*S*,3*R*,4*R*)-5-(7,8-dimethyl-2,4-dioxo-3,4-dihydrobenzo[*g*]pteridin-10(2*H*)-yl)pentane-1,2,3,4-tetrayl tetraacetate (RFTA)

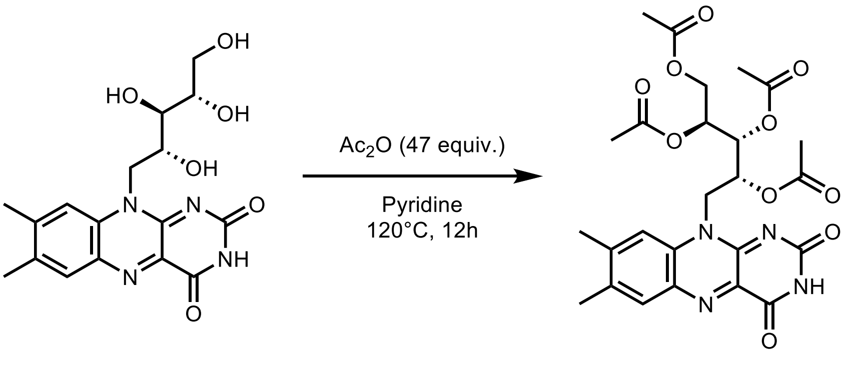

Riboflavin **Riboflavin tetraacetate (RFTA)**

According to a known procedure,[2] acetic anhydride (118 mL, 47 equiv.) was added to a solution of (–)-Riboflavin (10.0 g, 26.6 mmol, 1 equiv.) in pyridine (120 mL) in a round-bottom flask. After refluxing for 12 h at 120 °C, the reaction mixture was cooled to room temperature, diluted with CH_2Cl_2 (200 mL), poured into ice-chilled aq. HCl (1M, 200 mL) and extracted with CH_2Cl_2 (3 x 100 mL). The combined organic layers were washed with aqueous HCl (1 M, 100 mL) and water (100 mL), dried over Na_2SO_4, and concentrated under reduced pressure. Purification of the crude product by flash-column chromatography (SiO_2, 10% MeOH/CH_2Cl_2) afforded **RFTA** as a yellow-brown solid (5.30 g, 37%).

^{1}H NMR (500 MHz, $CDCl_3$) δ 8.61 (s, 1H), 8.02 (s, 1H), 7.56 (s, 1H), 5.66 (d, *J* = 8.9 Hz, 1H), 5.48 - 5.43 (m, 1H), 5.40 (ddd, *J* = 6.5, 5.8, 2.9 Hz, 1H), 4.43 (dd, *J* = 12.3, 2.9 Hz, 1H), 4.24 (dd, *J* = 12.4, 5.8 Hz, 1H), 2.56 (s, 3H), 2.44 (d, *J* = 1.0 Hz, 3H), 2.28 (s, 3H), 2.21 (s, 3H), 2.07 (s, 3H), 1.75 (s, 3H). **^{13}C NMR** (126 MHz, $CDCl_3$) δ 170.8, 170.4, 170.0, 159.5, 154.6, 150.9, 148.3, 137.2, 136.2, 134.8, 133.1, 131.4, 115.7, 70.6, 69.1, 62.0, 45.2, 21.6, 21.2, 20.9, 20.8, 20.5, 19.6.

The analytical data is consistent with those previously reported.[2]

3.2 General Procedure for the Synthesis of Non-Commercially Available Piperidines

General Procedure I

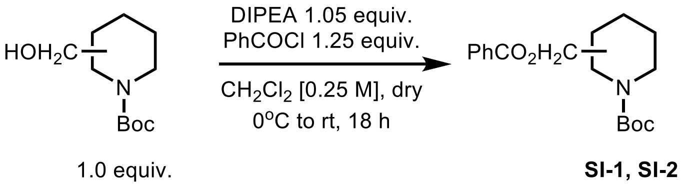

A flame-dried, round-bottom flask equipped with a magnetic stirrer was charged with a solution of the respective Boc-protected amine (10 mmol, 1 equiv., 0.25 M) in dry CH_2Cl_2 (100 mL) under a N_2 atmosphere. The solution was cooled to 0 °C and dry benzoyl chloride (12.5 mmol, 1.25 equiv., 3.6 mL) was added dropwise over 5-10 min. Subsequently, *N,N*-diisopropylethylamine (10.5 mmol, 1.05 equiv., 4.6 mL) was added dropwise over 5-10 min, the reaction mixture was stirred at 0 °C for an additional 15 min, allowed to warm to room temperature, and then stirred overnight (18 h). The reaction mixture was quenched with a saturated solution of sodium bicarbonate ($NaHCO_3$), the organic layer was separated, and the aqueous layer was extracted with CH_2Cl_2 (3x20 mL). The combined organic layers were washed with brine, dried over anhydrous Na_2SO_4, and filtered. The solvent was removed under reduced pressure and the product was purified by flash column chromatography.

3.3 Analytical Data

tert-Butyl 3-((benzoyloxy)methyl)piperidine-1-carboxylate (SI-1)

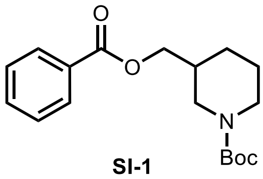

SI-1

Compound **SI-1** was synthesized according to General Procedure I starting from *tert*-butyl 3-(hydroxymethyl)piperidine-1-carboxylate. The crude was purified by flash column chromatography (SiO_2, ^{n}hexane:EtOAc 9:1) to furnish the product as a white solid in 64% yield (2.04 g).

^{1}H NMR (500 MHz, $CDCl_3$): δ 8.06 – 8.04 (m, 2H), 7.58 – 7.54 (m, 1H), 7.45 – 7.42 (m, 2H), 4.27 – 3.91 (m, 4H), 2.85 – 2.67 (m, 2H), 2.04 – 1.96 (m, 1H), 1.90 – 1.85 (m, 1H), 1.73 – 1.67 (m, 1H), 1.57-1.45 (m, 10H), 1.34 – 1.25 (m, 1H). **^{13}C NMR** (126 MHz, $CDCl_3$): δ 166.6, 155.0, 133.1, 130.3, 129.7, 128.5, 79.6, 66.9, 47.2, 44.5, 35.7, 28.6, 27.5, 24.5. **HRMS** (ESI) *m/z*: Calculated for $C_{18}H_{25}NNaO_4$ $[M+Na]^+$ 342.1676, found 342.1666.

tert-Butyl 2-((benzoyloxy)methyl)piperidine-1-carboxylate (SI-2)

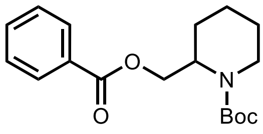

SI-2

Compound **SI-2** was synthesized according to General Procedure I starting from *tert*-butyl 2-(hydroxymethyl)piperidine-1-carboxylate. The crude was purified by flash column chromatography (SiO_2, ^{n}hexane:EtOAc 9:1) to furnish the product as a white solid in 53% yield (1.69 g).

^{1}H NMR (500 MHz, $CDCl_3$): δ 8.05 – 8.02 (m, 2H), 7.55 – 7.52 (m, 1H), 7.41 (t, *J* = 7.6 Hz, 2H), 4.66 (br s, 1H), 4.52 – 4.48 (m, 1H), 4.34 (br s, 1H), 4.06 (br s, 1H), 2.88 (td, *J* = 13.5, 1.9 Hz, 1H), 1.76 – 1.64 (m, 4H), 1.58 – 1.36 (m, 11H). **^{13}C NMR** (126 MHz, $CDCl_3$): δ 166.5, 155.1, 133.1, 130.2, 129.9, 128.4, 79.6, 62.4, 49.0, 39.3, 28.4, 25.7, 25.4, 19.6. **HRMS** (ESI) *m/z*: Calculated for $C_{18}H_{25}NNaO_4$ $[M+Na]^+$ 342.1676, found 342.1669.

The analytical data is consistent with that previously reported.[3]

tert-Butyl (*R*)-2-(((*S*)-1-methoxy-3-methyl-1-oxobutan-2-yl)carbamoyl)piperidine-1-carboxylate (39)

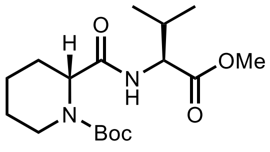

39

A flame-dried, round-bottomed flask equipped with a magnetic stirrer was charged with a solution of methyl *L*-valinate hydrochloride (10 mmol, 1 equiv., 1.7 g, 0.12 M) in dry CH_2Cl_2 (83.3 mL) under a N_2 atmosphere. The solution was cooled to 0 °C, *N,N*-diisopropylethylamine (10 mmol, 1 equiv., 1.7 mL) was added dropwise over 5-10 min, and the resulting solution was stirred at 0 °C for an additional 10 min. (*R*)-1-(*tert*-butoxycarbonyl)piperidine-2-carboxylic acid (10 mmol, 1 equiv., 2.3 g, 0.12 M), hydroxybenzotriazole (9 mmol, 0.9 equiv., 1.2 g), and 3-(((ethylimino)methylene)amino)-*N,N*-dimethylpropan-1-amine hydrochloride (10 mmol, 1 equiv., 1.9 g) were added and the resulting reaction mixture was allowed to warm to room temperature, and then stirred overnight for 18 h. The mixture was cooled to 0 °C and quenched with 1 M aqueous HCl (18 mmol, 1.8 equiv., 18 mL). The organic layer was separated, and the aqueous layer was extracted with CH_2Cl_2 (3x20 mL). The combined organic layers were washed with brine, dried over anhydrous Na_2SO_4 and filtered. The solvent was removed under reduced pressure and the crude was purified by flash column chromatography (SiO_2, ^{n}hexane:EtOAc 2:1) to furnish the product as a white solid in 81% yield (2.80 g).

^{1}H NMR (500 MHz, $CDCl_3$): δ 6.62 – 6.40 (m, 1H), 4.80 (br s, 1H), 4.54 (br s, 1H), 4.17 – 4.01 (m, 1H), 3.72 (m, 3H), 2.85 (apparent t, *J* = 12.9 Hz, 1H), 2.29 (br s, 1H), 2.22 – 2.15 (m, 1H), 1.62 – 1.34 (m, 14H), 0.94 – 0.92 (m, 3H), 0.86 – 0.85 (m, 3H). **^{13}C NMR** (126 MHz, $CDCl_3$): δ 172.5, 171.4, 80.7, 57.0, 55.8 (br), 53.9 (br), 52.2, 42.5 (br), 41.3 (br), 31.0, 28.5, 25.0, 20.6, 19.2, 17.6. **HRMS** (ESI) *m/z*: Calculated for $C_{17}H_{30}N_2NaO_5$ $[M+Na]^+$ 365.2047, found 365.2038.

4. Substrate Scope

4.1 Hemiaminals

4.1.1 General Procedure

General Procedure II: α-Hydroxylation

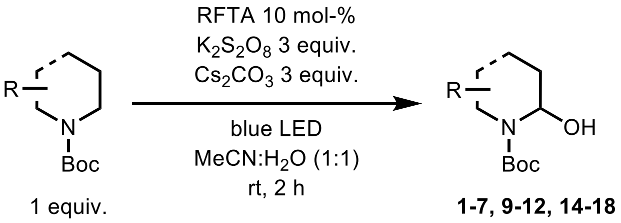

The Boc-protected amine (1.0 equiv., 0.3 mmol), $K_2S_2O_8$ (3.0 equiv., 0.9 mmol, 243 mg), Cs_2CO_3 (3.0 equiv., 0.9 mmol, 293 mg), MeCN:H_2O (1:1, 15 mL) and RFTA (5 mol%, 8.2 mg) were placed in an 8-dram vial equipped with a stirring bar. The mixture was sonicated until no solid was visible. The solution was irradiated for 1 h under stirring. Afterwards, more RFTA (5 mol%, 8.2 mg) was added, the mixture was briefly sonicated and further irradiated for an additional hour. Brine was added (5 mL) and the mixture was extracted with EtOAc (3x5 mL). The combined organic layers were dried over Na_2SO_4, filtered, and concentrated under a stream of N_2.

4.1.2 Analytical Data

***tert*-Butyl 2-hydroxypiperidine-1-carboxylate (1)**

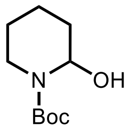

1

Hemiaminal **1** was prepared according to General Procedure II starting from *tert*-butyl piperidine-1-carboxylate. The crude was purified by preparative thin layer chromatography (SiO_2, ^{n}hexane:EtOAc 3:1) to furnish the product as a colorless oil in 56% yield (34 mg).

^{1}H NMR (400 MHz, $CDCl_3$): δ 5.71 (s, 1H), 3.79 (br d, *J* = 12.8 Hz, 1H), 3.09 (td, *J* = 12.8, 3.3 Hz, 1H), 1.90 - 1.84 (m, 1H), 1.78 - 1.65 (m, 2H), 1.63 - 1.52 (m, 2H), 1.47 (s, 9H), 1.45 - 1.41 (m, 1H). **^{13}C NMR** (101 MHz, $CDCl_3$): δ 155.4, 80.1, 74.6, 39.2, 30.5, 28.4, 24.8, 17.8.

The analytical data is consistent with that previously reported.[4]

tert-Butyl 2-hydroxy-4-phenylpiperidine-1-carboxylate (3)

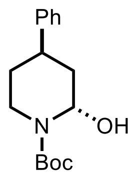

3

Hemiaminal **3** was prepared according to General Procedure II starting from *tert*-butyl 4-phenylpiperidine-1-carboxylate. The crude was purified by preparative thin layer chromatography (SiO_2, ^{n}hexane:EtOAc 3:1) to furnish the product as a clear oil in 60% yield (50 mg).

^{1}H NMR (500 MHz, $CDCl_3$): δ 7.33 – 7.29 (m, 2H), 7.24 – 7.20 (m, 3H), 5.89 (br s, 1H), 3.96 (br s, 1H), 3.27 (td, *J* = 12.8, 3.0 Hz, 1H), 3.14 (tt, *J* = 12.7, 3.5 Hz, 1H), 2.1 (ddt, *J* = 13.6, 3.4, 2.3 Hz, 1H), 1.90 (dt, *J* = 13.1, 2.9 Hz, 1H), 1.78 (tdd, *J* = 13.4, 3.6, 1.6 Hz, 1H), 1.70 (qd, *J* = 12.8, 4.5 Hz, 1H), 1.50 (s, 9H). **^{13}C NMR** (126 MHz, $CDCl_3$): δ 145.7, 128.7, 127.0, 126.5, 80.6, 74.9 (br), 39.5 (br), 38.4, 35.5, 32.6, 28.6 (*Note*: The signal corresponding to the Boc carbonyl C is not visible.). **HRMS** (ESI) *m/z*: Calculated for $C_{16}H_{23}NNaO_3$ $[M+Na]^+$ 300.1570, found 300.1576.

Assignment of the relative stereochemistry:

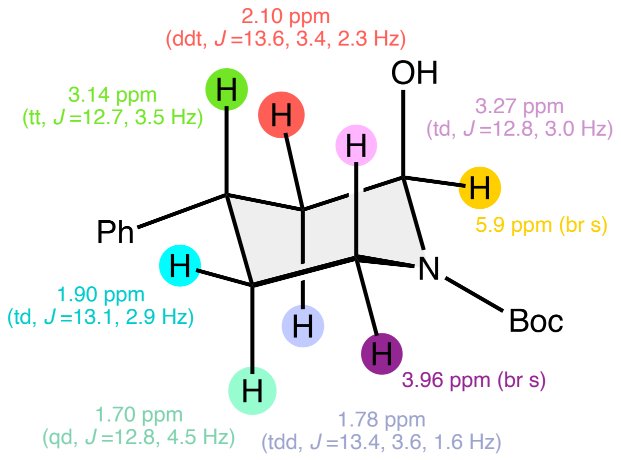

tert-Butyl 4-acetyl-2-hydroxypiperidine-1-carboxylate (4)

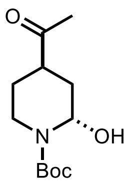

4

Hemiaminal **4** was synthesized according to General Procedure II starting from *tert*-butyl 4-acetylpiperidine-1-carboxylate. The crude was purified by preparative thin layer chromatography (SiO_2, ^{n}hexane:EtOAc 2:1) to furnish the product as a colorless oil in 62% yield (45 mg).

^{1}H NMR (500 MHz, $CDCl_3$): δ 5.83 - 5.61 (m, 1H), 3.87 - 3.80 (m, 1H), 3.14 (td, *J* = 13.1, 3.2 Hz, 1H), 2.91 (tt, *J* = 12.4, 3.5 Hz), 2.16 (s, 3H), 2.08 - 2.05 (m, 1H), 1.90 - 1.88 (m, 1H), 1.60 - 1.54 (m, 1H), 1.51 - 1.46 (m, 1H), 1.45 (s, 9H). **^{13}C NMR** (126 MHz, $CDCl_3$): δ 210.6, 155.1, 80.6, 74.1 (73.8), 42.8, 38.3, 32.5, 28.4, 28.1, 26.9. **HRMS** (ESI) *m/z*: Calculated for $C_{12}H_{21}NNaO_4$ $[M+Na]^+$ 266.1363, found 266.1369.

1-(*tert*-Butyl) 4-methyl 2-hydroxypiperidine-1,4-dicarboxylate (5)

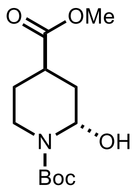

5

Hemiaminal **5** was synthesized according to General Procedure II starting from 1-(*tert*-butyl) 4-methyl piperidine-1,4-dicarboxylate. The crude was purified by flash column chromatography (SiO_2, ^{n}hexane:EtOAc 2:1) to furnish the product as a colorless oil in 56% yield (41 mg).

^{1}H NMR (500 MHz, $CDCl_3$): δ 5.79 - 5.61 (m, 1H), 3.66 (s, 3H), 3.84 (br s, 1H), 3.11 (td, *J* = 13.1, 3.1 Hz, 1H), 2.86 (tt, *J* = 12.5, 3.6 Hz, 1H), 2.12 (ddt, *J* = 13.6, 3.8, 2.2 Hz, 1H), 1.99 – 1.89 (m, 1H), 1.66 (td, *J* = 13.1, 3.5 Hz, 1H), 1.55 (qd, *J* = 13.1, 4.7 Hz, 1H), 1.44 (s, 9H). **^{13}C NMR** (126 MHz, $CDCl_3$): δ 175.6, 155.2, 80.7, 74.3, 51.9, 38.3, 35.3, 33.3, 28.5, 27.7. **HRMS** (ESI) *m/z*: Calculated for $C_{12}H_{21}NNaO_5$ $[M+Na]^+$ 282.1312, found 282.1315.

***tert*-Butyl 2-hydroxy-4-methylpiperidine-1-carboxylate (6)**

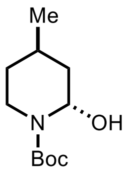

6

Hemiaminal **6** was synthesized according to General Procedure II starting from *tert*-butyl 4-methylpiperidine-1-carboxylate. The crude was purified by preparative thin layer chromatography (SiO_2, ^{n}hexane:EtOAc 2:1) to furnish the product as a colorless oil in 49% yield (31 mg).

^{1}H NMR (500 MHz, $CDCl_3$): δ 5.73 (s, 1H), 3.81 (s, 1H), 3.08 (td, J = 13.0, 3.0 Hz, 1H), 1.99 – 1.82 (m, 2H), 1.65 (d, J = 14.0 Hz, 1H) 1.47 (s, 9H), 1.28 – 1.16 (m, 1H), 1.16 – 1.03 (m, 1H), 0.92 (d, *J* = 6.6 Hz, 3H). **^{13}C NMR** (126 MHz, $CDCl_3$): δ 155.5, 80.2, 75.0, 39.2 (br), 39.1, 33.4, 28.4, 28.4, 24.2, 21.9. **HRMS** (ESI) *m/z*: Calculated for $C_{11}H_{21}NNaO_3$ $[M+Na]^+$ 238.1414, found 238.1412.

***tert*-Butyl 7-hydroxy-1,4-dioxa-8-azaspiro[4.5]decane-8-carboxylate (7)**

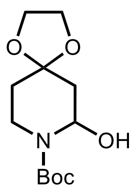

7

Hemiaminal **7** was synthesized according to General Procedure II starting from *tert*-butyl 1,4-dioxa-8-azaspiro[4.5]decane-8-carboxylate. The crude was purified by preparative thin layer chromatography (SiO_2, ^{n}hexane:EtOAc 2:1) to furnish the product as a colorless oil in 24% yield (19 mg).

^{1}H NMR (400 MHz, $CDCl_3$): δ 5.80 (s, 1H), 4.26 (d, *J* = 9.6 Hz, 1H), 4.07 – 3.96 (m, 4H), 3.28 – 3.22 (m, 1H), 1.98 – 1.88 (m, 2H), 1.75 – 1.69 (m, 2H), 1.47 (s, 9H). **^{13}C NMR** (101 MHz, $CDCl_3$): δ 154.7, 107.5, 80.5, 76.0, 65.0, 64.4, 39.2, 36.4, 34.0, 28.5. **HRMS** (ESI) *m/z*: Calculated for $C_{12}H_{19}NNaO_5$ $[M+Na]^+$ 280.1155, found 280.1150.

tert-Butyl 4-hydroxypiperidine-1-carboxylate (8b)

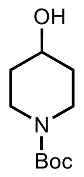

8b

Hemiaminal **8b** was synthesized according to General Procedure II starting from *tert*-butyl 4-(4,4,5,5-tetramethyl-1,3,2-dioxaborolan-2-yl)piperidine-1-carboxylate. The crude was purified by preparative thin layer chromatography (SiO_2, ^{n}hexane:EtOAc 4:1) to furnish the product as a colorless oil in 71% yield (43 mg).

^{1}H NMR (400 MHz, $CDCl_3$): δ 3.87 – 3.79 (m, 3H), 3.01 (ddd, *J* = 13.4, 9.7, 3.4 Hz, 2H), 1.87 – 1.81 (m, 2H), 1.49 – 1.44 (m, 11H). **^{13}C NMR** (101 MHz, $CDCl_3$): δ 155.0, 79.7, 67.9, 41.4, 34.3, 28.6. **HRMS** (ESI) *m/z*: Calculated for $C_{10}H_{19}NNaO_3$ $[M+Na]^+$ 224.1263, found 224.1263.

The analytical data is consistent with those previously reported.[5]

tert-Butyl 5-cyano-2-hydroxypiperidine-1-carboxylate (9a) and **tert-butyl 3-cyano-2-hydroxypiperidine-1-carboxylate (9b)**

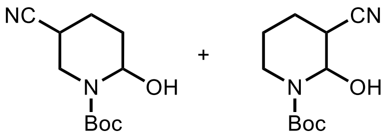

9a **9b**

Hemiaminals **9a** and **9b** were synthesized according to General Procedure II starting from *tert*-butyl 3-cyanopiperidine-1-carboxylate. The crude was purified by preparative thin layer chromatography (SiO_2, ^{n}hexane:EtOAc 2:1) to furnish a mixture of regioisomers **9a**:**9b** (7.5:1) as a colorless oil in 27% overall yield (18 mg).

^{1}H NMR (500 MHz, $CDCl_3$): δ 5.92-5.90 (m, 1H, **9b**, *major*), 5.82 (br s, 1H, **9a**, *major*), 5.73 (br s, 1H, **9a**, *minor*), 4.12-4.06 (m, 1H, **9a**, *major* and 1H, **9a**, *minor*), 3.89 (br s, 1H, **9b**, *major*), 3.35-3.26 (m, 1H, **9a**, *major* and 1H, **9a**, *minor*), 3.13-3.06 (m, 1H, **9b**, *major*), 3.03 (br s, 1H, **9b**, *major*), 2.91 (br s, 1H, **9a**, *major*), 2.59 (tt, *J* = 11.9, 4.1 Hz, 1H, **9a**, *minor*), 2.15-1.85 (m, 4H, **9a**, *major* and 4H, **9a**, *minor* and 4H, **9b**, *major*), 1.48-1.46 (m, 9H, **9a**, *major* and 9H, **9a**, *minor* and 9H, **9b**, *major*). **^{13}C NMR** (126 MHz, $CDCl_3$): δ 154.7 (**9a**, *major*), 154.4 (**9a**, *minor*), 120.3 (**9a**, *major*), 120.2 (**9a**, *minor*), 119.1 (**9b**, *major*), 81.6 (**9a**, *minor*), 81.5 (**9a**, *major*), 81.5 (**9b**, *major*), 74.4 (br, **9b**, *major*), 73.8 (br, **9a**, *major*), 73.3 (br, **9a**, *minor*), 40.3 (br, **9a**, *major* and **9a**, *minor*), 38.4 (**9b**, *major*)[§], 32.4 (**9b**, *major*), 29.4 (**9a**, *minor*), 28.4 (**9a**, *minor*), 28.4 (**9b**, *major*), 28.4 (**9a**, *major*), 27.3 (**9a**, *minor*), 26.9 (**9a**, *major*), 26.8 (**9a**, *major*), 22.2 (**9a**, *minor*), 21.2 (**9b**, *major*), 21.0 (**9a**, *major*), 20.87 (**9b**, *major*). **HRMS** (ESI) *m/z*: Calculated for $C_{11}H_{18}N_2NaO_3$ $[M+Na]^+$ 249.1210, found 249.1207.

[§]Elucidated by HSQC.

1-(*tert*-Butyl) 3-methyl 6-hydroxypiperidine-1,3-dicarboxylate (10a) and **1-(*tert*-butyl) 3-methyl 2-hydroxypiperidine-1,3-dicarboxylate (10b)**

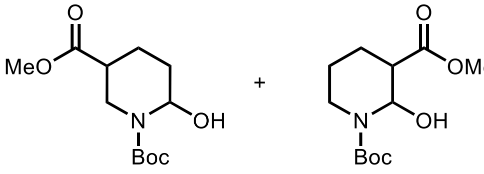

10a **10b**

Hemiaminals **10a** and **10b** were synthesized according to General Procedure II starting from 1-(*tert*-butyl) 3-methyl piperidine-1,3-dicarboxylate. The crude was purified by preparative thin layer chromatography (SiO_2, ^{n}hexane:EtOAc 3:1) to furnish a mixture of regioisomers **10a**:**10b** (3:1) as a colorless oil in 50% overall yield (39 mg).

^{1}H NMR (500 MHz, $CDCl_3$): δ 6.01-5.99 (m, 1H, **10b**, *major*), 5.70 (br s, 1H, **10a**, *major*), 5.65 (br s, 1H, **10a**, *minor*), 4.16-4.13 (m, 1H, **10a**, *minor*), 4.00 (br s, 1H, **10a**, *major*), 3.83-3.65 (m, 3H, **10a**, *major* and 3H, **10a**, *minor*), 3.37-3.33 (m, 1H, **10a**, *minor*), 3.18-3.13 (m, 1H, **10a**, *major*), 3.08-3.01 (m, 1H, **10b**, *major*), 2.64-2.62 (m, 1H, **10a**, *minor*), 2.53-2.48 (m, 1H, **10b**, *major*), 2.46-2.39 (m, 1H, **10a**, *major*), 1.94-1.42 (m, 13H, **10a**, *major* and 13H, **10a**, *minor* and 13H, **10b**, *major*). **^{13}C NMR** (126 MHz, $CDCl_3$): δ 173.86 (**10a**, *major*), 173.84 (**10a**, *minor*), 173.59 (**10b**, *major*)§, 155.07 (br, **10a**, *major* and **10a**, *minor* and **10b**, *major*), 80.75 (**10a**, *major*), 80.60 (**10b**, *major*), 80.44 (**10a**, *minor*), 74.47 (br, **10a**, *minor* and **10b**, *major*), 73.58 (br, **10a**, *major*), 52.01 (**10b**, *major*), 51.90 (**10a**, *minor*), 51.86 (**10a**, *major*), 46.13 (**10b**, *major*), 41.31 (**10a**, *major*), 40.03 (br, **10a**, *major*), 39.64 (br, **10a**, *minor*), 38.67 (**10a**, *minor*), 38.57 (br, **10b**, *major*), 29.92 (**10a**, *major*), 28.43 (**10a**, *major* and **10a**, *minor* and **10b**, *major*), 27.21 (**10a**, *minor*), 24.26 (**10b**, *major*), 21.32 (**10a**, *major*), 20.38 (**10b**, *major*), 19.25 (**10a**, *minor*). **HRMS** (ESI) *m/z*: Calculated for $C_{12}H_{21}NNaO_5$ $[M+Na]^+$ 282.1312, found 282.1305.

§Elucidated by HSQC.

1-(*tert*-Butyl) 2-methyl 6-hydroxypiperidine-1,2-dicarboxylate (11)

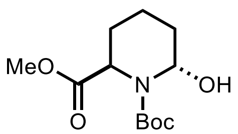

11

Hemiaminal **11** was synthesized according to General Procedure II starting from 1-(*tert*-butyl) 2-methyl piperidine-1,2-dicarboxylate. The crude was purified by preparative thin layer chromatography (SiO_2, ^{n}hexane:EtOAc 2:1) to furnish the product as a colorless oil in 15% yield (11 mg).

^{1}H NMR (500 MHz, $CDCl_3$): δ 5.72 – 5.64 (m, 1H), 4.79 – 4.63 (m, 1H), 3.76 (s, 3H), 2.19 - 2.17 (m, 1H), 1.90 (br s, 1H), 1.78 – 1.65 (m, 2H), 1.49 – 1.44 (m, 11H) **^{13}C NMR** (126 MHz, $CDCl_3$): δ 155.7, 81.3, 75.0, 74.2, 53.9, 53.6, 53.0, 52.6, 31.7, 30.9, 28.4, 26.4, 26.1, 14.3 (The ester carbonyl signal is not visible.). **HRMS** (ESI) *m/z*: Calculated for $C_{12}H_{21}NNaO_5$ $[M+Na]^+$ 282.1312, found 282.1315.

1-(*tert*-butyl) 2-methyl (2*R*,6*S*)-6-hydroxypiperidine-1,2-dicarboxylate ((2*R*)-11)

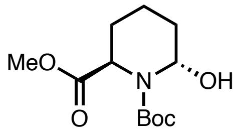

(2*R*)-11

Hemiaminal **(2*R*)-11** was synthesized according to General Procedure II starting from 1-(*tert*-butyl) 2-methyl (2*R*)-6-hydroxypiperidine-1,2-dicarboxylate. The crude was purified by preparative thin layer chromatography (SiO_2, ^{n}hexane:EtOAc 2:1) to furnish the product as a colorless oil in 14% yield (11 mg).

The NMR spectra are in agreement with those of **11**.

Comparison by chiral stationary-phase HPLC analysis of the reactions yielding **11** and **(2*R*)-11** corroborated the stereochemical integrity of the stereocenter at C^{α}: IG column; hexane/^{i}PrOH 95:5; flow rate: 5.0 mL/min; temperature: 25 °C; detection at 220 nm. 46.0 min (corresponding to the trans product derived from 1-(tert-butyl) 2-methyl (2*R*)-6-hydroxypiperidine-1,2-dicarboxylate).

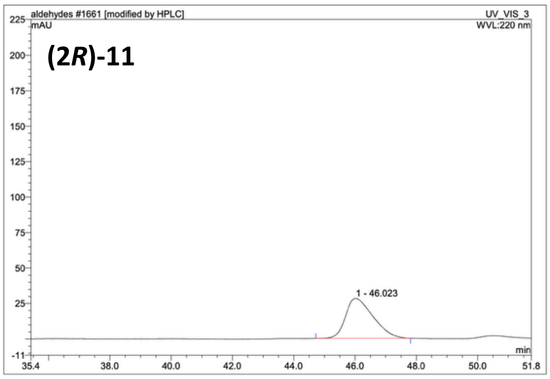

No.	Ret.Time min		Peak Name	Height mAU	Area mAU*min	Rel.Area %	Amount	Type
1	46.02	n.a.		28.206	29.288	100.00	n.a.	BMB*
Total:				28.206	29.288	100.00	0.000	

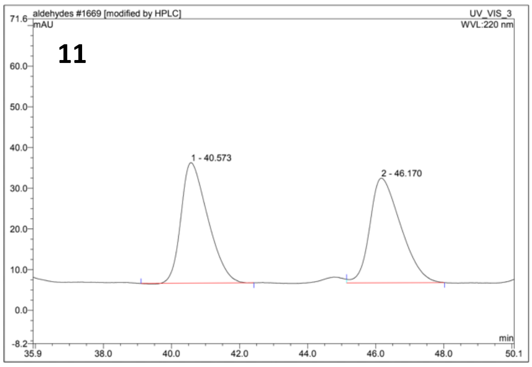

No.	Ret.Time min		Peak Name	Height mAU	Area mAU*min	Rel.Area %	Amount	Type
1	40.57	n.a.		29.623	27.018	49.88	n.a.	BMB*
2	46.17	n.a.		25.724	27.143	50.12	n.a.	MB*
Total:				55.347	54.160	100.00	0.000	

***tert*-Butyl 2-cyano-6-hydroxypiperidine-1-carboxylate (12)**

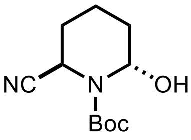

12

Hemiaminal **12** was synthesized according to General Procedure II starting from *tert*-butyl-2-cyanopiperidine-1-carboxylate. The crude was purified by preparative thin layer chromatography (SiO_2, ^{n}hexane:EtOAc 4:1) to furnish the product as a colorless oil in 43% yield (29 mg).

^{1}H NMR (500 MHz, $CDCl_3$): δ 5.72 (br s, 1H), 4.94 (br s, 1H), 2.19 – 2.01 (m, 3H), 1.80 – 1.72 (m, 1H), 1.69 – 1.64 (m, 1H), 1.60 – 1.56 (m, 1H), 1.52 (s, 9H). **^{13}C NMR** (126 MHz, $CDCl_3$): δ 119.7, 82.8, 74.5§, 42.4§, 30.0, 28.4, 28.1, 14.4 (*Note*: The signal corresponding to the Boc carbonyl C is not visible.). **HRMS** (ESI) *m/z*: Calculated for $C_{11}H_{18}N_2NaO_3$ $[M+Na]^+$ 249.1210, found 249.1210.

§Elucidated by HSQC.

***tert*-Butyl (5-oxo-5-phenylpentyl)carbamate (13b)**

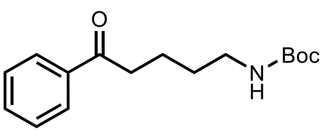

13b

Hemiaminal **13b** was synthesized according to General Procedure II starting from *tert*-butyl 2-phenylpiperidine-1-carboxylate. The crude was purified by preparative thin layer chromatography (SiO_2, ^{n}hexane:EtOAc 4:1) to furnish the product as a white solid in 77% yield (64 mg).

^{1}H NMR (MHz, $CDCl_3$): δ 7.97 – 7.94 (m, 2H), 7.56 (tt, *J* = 7.4, 1.2, 1H), 7.46 (t, *J* = 7.5 Hz, 2H), 4.59 (br s, 1H), 3.17 (q, *J* = 6.1 Hz, 2H), 3.01 (t, *J* = 7.2 Hz, 2H), 1.82 – 1.74 (m, 2H), 1.62 – 1.56 (m, 2H), 1.44 (s, 9H). **^{13}C NMR** (101 MHz, $CDCl_3$): δ 200.0, 136.9, 133.2, 128.8, 128.2, 79.1, 40.3, 38.1, 29.6, 28.6, 21.4 (*Note*: The signal corresponding to the Boc carbonyl C is not visible.). **HRMS** (ESI) *m/z*: Calculated for $C_{16}H_{23}NNaO_3$ $[M+Na]^+$ 300.1570, found 300.1569

The analytical data is consistent with those previously reported.[6]

Di-*tert*-butyl 2-hydroxypiperazine-1,4-dicarboxylate (14)

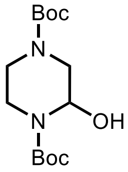

14

Hemiaminal **14** was synthesized according to General Procedure II starting from di-tert-butyl piperazine-1,4-dicarboxylate. The crude was purified by preparative thin layer chromatography (SiO_2, ^{n}hexane:EtOAc 3:1) to furnish the product as a colorless oil in 55% yield (determined by ^{1}H-NMR against $C_2H_2Cl_4$ as internal standard).

^{1}H NMR (500 MHz, $CDCl_3$): δ 5.55 (s, 1H), 4.07 – 3.58 (m, 3H), 3.38 – 2.97 (m, 4H), 1.48 (s, 9H), 1.47 (s, 9H). **^{13}C NMR** (126 MHz, $CDCl_3$): δ 155.5, 81.2, 80.4, 74.3, 47.6 (br), 43.5 (42.5), 38.9, 28.5, 28.5. **HRMS** (ESI) *m/z*: Calculated for $C_{14}H_{26}N_2NaO_5$ $[M+Na]^+$ 325.1734, found 325.1731.

***tert*-Butyl 3-hydroxymorpholine-4-carboxylate (15)**

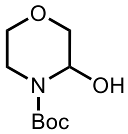

15

Hemiaminal **15** was synthesized according to General Procedure II starting from *tert*-butyl morpholine-4-carboxylate. The crude was purified by flash column chromatography (SiO_2, ^{n}hexane:EtOAc 2:1) to furnish the product as a colorless oil in 54% yield (33 mg).

^{1}H NMR (500 MHz, $CDCl_3$): δ 5.39 (s, 1H), 3.93 - 3.91 (m, 2H), 3.62 - 3.49 (m, 3H), 3.35 (br s, 1H), 1.47 (s, 9H). **^{13}C NMR** (126 MHz, $CDCl_3$): δ 155.1, 81.0, 73.4, 70.5, 66.5, 39.5, 28.3. **HRMS** (ESI) *m/z*: Calculated for $C_9H_{17}NNaO_4$ $[M+Na]^+$ 226.1050, found 226.1044.

***tert*-Butyl 2-hydroxypyrrolidine-1-carboxylate (16)**

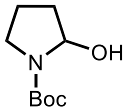

16

Hemiaminal **16** was synthesized according to General Procedure II starting from *tert*-butyl pyrrolidine-1-carboxylate. The crude was purified by flash column chromatography (SiO_2, ^{n}hexane:EtOAc 2:1) to furnish the product as a colorless oil in 49% yield (28 mg).

^{1}H NMR (500 MHz, $CDCl_3$): δ 5.47 - 5.38 (m, 1H), 3.56 – 3.43 (m, 1H), 3.34 – 3.20 (m, 1H), 2.10 – 1.76 (m, 4H),1.48 (s, 9H). **^{13}C NMR** (126 MHz, $CDCl_3$): δ 155.1 (153.6), 81.8 (81.5), 80.0 (80.4), 45.9 (45.8), 32.7 (33.5), 28.5 (28.6), 22.7 (22.1). Values in brackets are from the cis-isomer.

The analytical data is consistent with those previously reported.[Error! Unknown switch argument.]

***tert*-butyl 2-hydroxyazepane-1-carboxylate (17)**

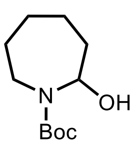

17

Hemiaminal **17** was synthesized according to General Procedure II starting from *tert*-butyl azepane-1-carboxylate. The crude was purified by flash column chromatography (SiO_2, ^{n}hexane:EtOAc 2:1) to furnish the product as a white crystalline solid in 62% yield (28 mg).

^{1}H NMR (500 MHz, $CDCl_3$): δ 5.47 (br d, 1H), 3.78 - 3.59 (br d, 1H), 3.14 - 3.04 (m, 1H), 2.21 - 2.14 (m, 1H), 1.80 - 1.56 (m, 5H), 1.51 - 1.49 (m, 9H), 1.41 - 1.27 (m, 2H). **^{13}C NMR** (126 MHz, $CDCl_3$): δ 156.5, (155.2), 80.1, 79.9 (80.5), 40.4 (41.0), 34.2 (34.5), 29.7, 29.5, 28.5, 23.0. Values in brackets relate to the *cis*-isomer.

The analytical data is consistent with those previously reported.[Error! Unknown switch argument.]

***tert*-Butyl butyl(hydroxymethyl)carbamate (18)**

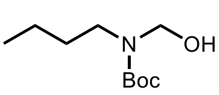

18

Hemiaminal **18** was synthesized according to General Procedure II starting from *tert*-butyl butyl(methyl)carbamate. The crude was purified by flash column chromatography (SiO_2, ^{n}hexane:EtOAc 2:1) to furnish the product as a colorless oil in 13% yield (7.7 mg).

^{1}H NMR (500 MHz, $CDCl_3$): δ 4.72 (d, J = 7.6 Hz, 2H), 3.28 (d, J = 6.5 Hz, 2H), 1.55 - 1.50 (m, 2H), 1.48 (s, 9H), 1.31 (sext, J = 7.4 Hz, 2H), 0.93 (t, J = 7.4 Hz, 3H). **^{13}C NMR** (126 MHz, $CDCl_3$): δ 156.4,[§] 80.5,[§] 73.1, 47.4, 31.8, 28.6, 20.1, 14.0. **HRMS** (ESI) *m/z*: Calculated for $C_{10}H_{21}NNaO_3$ $[M+Na]^+$ 226.1414, found 226.1411. [§]Elucidated via HMBC.

***tert*-Butyl-(6*R*)-2-hydroxy-6-(((*S*)-1-methoxy-3-methyl-1-oxobutan-2-yl)carbamoyl)piperidine-1-carboxylate (40)**

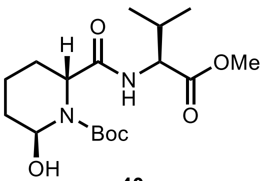

40

Hemiaminal **40** was synthesized according to General Procedure II starting from **39**. The crude was purified by preparative thin layer chromatography (SiO_2, ^{n}hexane:EtOAc 1:1) to furnish the product as a white solid in 32% yield (34 mg).

^{1}H NMR (500 MHz, $CDCl_3$, mixture of *cis-trans* isomers): δ 8.30 (br s, 0.5H), 7.46 (br s, 0.5H), 5.76 – 5.67 (m, 1H), 4.69 – 4.43 (m, 2H), 3.69 – 3.68 (m, 3H), 2.20 – 1.41 (m, 16H), 0.91 – 0.83 (m, 6H). **^{13}C NMR** (126 MHz, $CDCl_3$): δ 174.2, 172.9, 172.5, 155.7, 81.6, 81.4, 74.9, 73.9, 57.8, 57.3, 57.0, 54.1, 52.3, 52.2, 52.1, 31.5, 31.2, 30.8, 30.5, 28.3, 27.2, 26.8, 19.4, 19.2, 17.7, 17.6, 13.9 **^{1}H NMR** (500 MHz, DMSO): δ 9.03 (d, *J* = 8.6 Hz, 1H), 6.83 – 6.76 (m, 1H), 5.58 (br s, 1H), 4.41 – 4.39 (m, 1H), 4.18 (br s, 1H), 3.63 (s, 3H), 2.12 – 2.01 (m, 1H), 1.94 – 1.87 (m, 1H), 1.77 – 1.37 (m, 14H), 0.86 – 0.82 (m, 6H). **HRMS** (ESI) *m/z*: Calculated for $C_{17}H_{30}N_2NaO_6$ $[M+Na]^+$ 381.1996, found 381.1988. Note: The stereochemical assignment was carried out by NMR spectroscopic analysis. The observed NOEs and coupling constants support a relative *trans* configuration. Exchange signals in the NOESY indicate *cis*/*trans* isomers around the *N*-Boc moiety.

4.2 Enecarbamates

4.2.1 General Procedure

General Procedure III: Desaturation Protocol

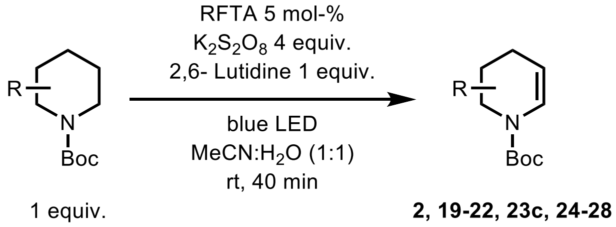

The Boc-protected amine (1.0 equiv., 0.3 mmol), $K_2S_2O_8$ (4.0 eq., 1.2 mmol, 324 mg), 2,6-lutidine (1.0 eq., 0.3 mmol, 34.7 µL), $MeCN:H_2O$ (1:1, 15 mL) and RFTA (5 mol%, 8.2 mg) were placed in an 8-dram vial equipped with a stirring bar and the mixture was sonicated until no solid was visible. The solution was irradiated for 40 min under stirring. Afterwards, brine was added (5 mL) and the mixture was extracted with EtOAc (3x5 mL). The combined organic layers were dried over $MgSO_4$, filtered, and concentrated under a stream of N_2.

4.2.2 Analytical Data

***tert*-Butyl 3,4-dihydropyridine-1(2*H*)-carboxylate (2)**

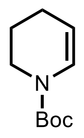

2

Enecarbamate **2** was synthesized according to General Procedure III starting from *tert*-butyl-piperidine-1-carboxylate. The crude was purified by preparative thin layer chromatography (SiO_2, ^{n}hexane:EtOAc 8:1) to furnish the product as a colorless oil in 76% yield (determined by GC-FID analysis).

^{1}H NMR (400 MHz, $CDCl_3$): δ 6.89 – 6.63 (m, 1H), 4.94 – 4.71 (m, 1H), 3.57-3.49 (m, 2H), 2.01 (tdd, *J* = 6.1, 3.9, 2.0 Hz, 2H), 1.83-1.75 (m, 2H), 1.47 (s, 9H). **^{13}C NMR** (101 MHz, $CDCl_3$): δ 152.5 (152.9), 125.8 (125.4), 105.3 (105.8), 80.6 (80.4), 41.6 (42.7), 28.5 (28.6), 21.9, 21.6. Values in brackets relate to the *cis*-isomer.

The analytical data is consistent with those previously reported.[Error! Unknown switch argument.]

***tert*-Butyl 4-phenyl-3,4-dihydropyridine-1(2*H*)-carboxylate (19)**

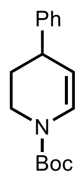

19

Enecarbamate **19** was synthesized according to General Procedure III starting from *tert*-butyl 4-phenylpiperidine-1-carboxylate. The crude was purified by preparative thin layer chromatography (SiO_2, ^{n}hexane:EtOAc 7:1) to furnish the product as a colorless oil in 57% yield (determined by ^{1}H-NMR spectroscopy against $C_2H_2Cl_4$ as internal standard).

^{1}H NMR (500 MHz, $CDCl_3$): δ 7.33 – 7.19 (m, 5H), 7.11 – 6.92 (m, 1H), 5.01 – 4.82 (m, 1H), 3.69 – 3.46 (m, 3H), 2.14 (br s, 1H), 1.84 – 1.76 (m, 1H), 1.52 (s, *9H*). **^{13}C NMR** (126 MHz, $CDCl_3$): δ 152.4 (152.8), 145.4, 128.5, 127.8, 126.5, 126.2 (126.5), 107.9 (108.3), 80.9 (80.8), 39.8 (40.7), 38.3 (38.1), 31.3, 28.5. **HRMS** (ESI) *m/z*: Calculated for $C_{16}H_{22}NO_2$ $[M+H]^+$ 260.1651, found 260.1646. Values in brackets relate to the *cis*-isomer.

tert-Butyl 4-acetyl-3,4-dihydropyridine-1(2H)-carboxylate (20)

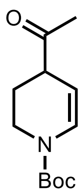

20

Enecarbamate **20** was synthesized according to General Procedure III starting from *tert*-butyl 4-acetylpiperidine-1-carboxylate. The crude was purified by preparative thin layer chromatography (SiO_2, ^{n}hexane:EtOAc 3:1) to furnish the product as a colorless oil in 69% yield (determined by ^{1}H-NMR spectroscopy against $C_2H_2Cl_4$ as internal standard).

^{1}H NMR (500 MHz, $CDCl_3$): δ 7.06 – 6.83 (m, 1H), 5.08 – 4.81 (m, 1H), 3.64 (ddd, *J* = 13.0, 6.8, 3.8 Hz, 1H), 3.54 – 3.35 (m, 1H), 3.08 (br s, 1H), 2.20 (s, 3H), 2.15 – 2.07 (m, 1H), 1.89 – 1.71 (m, 1H), 1.46 (s, 9H). **^{13}C NMR** (126 MHz, $CDCl_3$): δ 208.18, 152.1 (152.7), 127.4 (127.1), 101.1 (101.3), 81.2, 45.7 (45.5), 39.58 (40.6,), 28.4, 28.2, 23.2 (22.8). Values in brackets relate to the *cis*-isomer.

The analytical data is consistent with those previously reported.[7]

1-(tert-Butyl) 4-methyl 3,4-dihydropyridine-1,4(2H)-dicarboxylate (21)

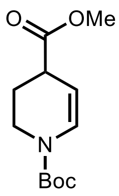

21

Enecarbamate **21** was synthesized according to General Procedure III starting from 1-(*tert*-butyl) 4-methyl piperidine-1,4-dicarboxylate. The crude was purified by preparative thin layer chromatography (SiO_2, ^{n}hexane:EtOAc 2:1) to furnish the product as a colorless oil in 42% yield (30 mg).

^{1}H NMR (500 MHz, $CDCl_3$): δ 7.01 – 6.77 (m, 1H), 5.00 – 4.81 (m, 1H), 3.69 (s, 3H), 3.66 – 3.49 (m, 2H), 3.11 (s, 1H), 2.14 – 2.04 (m, 1H), 1.93 (s, 1H), 1.46 (s, 9H). **^{13}C NMR** (126 MHz, $CDCl_3$): δ 174.0, 152.1 (152.6), 127.2 (126.8), 101.5 (101.9), 81.1 (81.0), 52.1, 39.5 (40.5), 37.5 (37.2), 28.4, 24.1 (24.0). Values in brackets relate to the *cis*-isomer.

The analytical data is consistent with those previously reported.[8]

tert-Butyl 4-methyl-3,4-dihydropyridine-1(2H)-carboxylate (22)

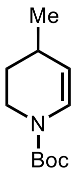

22

Enecarbamate **22** was synthesized according to General Procedure III starting from *tert*-butyl 4-methylpiperidine-1-carboxylate. The crude was purified by preparative thin layer chromatography (SiO_2, ^{n}hexane:EtOAc 8:1) to furnish the product as a colorless oil in 58% yield (determined by ^{1}H-NMR spectroscopy against $C_2H_2Cl_4$ as internal standard).

^{1}H NMR (500 MHz, $CDCl_3$): δ 6.86 – 6.59 (m, 1H), 4.81 – 4.62 (m, 1H), 3.77 – 3.62 (m, 1H), 3.37 (ddd, *J* = 13.0, 9.8, 3.3 Hz, 1H), 2.31 – 2.23 (m, 1H), 1.88 (br s, 1H), 1.48 (s, 9H), 1.45 – 1.39 (m, 1H), 1.00 (d, *J* = 7.0 Hz, 3H). **^{13}C NMR** (126 MHz, $CDCl_3$): δ 152.5, 124.6 (124.3), 111.7 (112.2), 80.6 (80.5), 40.2 (41.2), 30.2, 28.6, 27.0 (26.8), 21.5. Values in brackets relate to the *cis*-isomer.

The analytical data is consistent with those previously reported.[9]

tert-Butyl 4-oxo-3,4-dihydropyridine-1(2H)-carboxylate (23c)

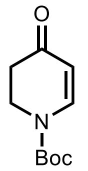

23c

Enecarbamate **23c** was synthesized according to General Procedure III starting from *tert*-butyl 4-oxopiperidine-1-carboxylate. The crude was purified by preparative thin layer chromatography (SiO_2, ^{n}hexane:EtOAc 6:1) to furnish the product as a colorless oil in 30% yield (18 mg).

^{1}H NMR (400 MHz, $CDCl_3$): δ 7.79 (br s, 1H), 5.32 – 5.24 (br d, J = 8.4 Hz, 1H), 3.95 (t, J = 7.4 Hz, 2H), 2.52 (t, J = 7.4 Hz, 2H), 1.52 (s, 9H). **^{13}C NMR** (101 MHz, $CDCl_3$): δ 193.7, 151.4, 144.1, 106.8, 83.6, 42.4, 35.8, 28.2.

The analytical data is consistent with those previously reported.[10]

tert-Butyl 4-methoxy-3,4-dihydropyridine-1(2H)-carboxylate (24)

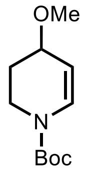

24

Enecarbamate **24** was synthesized according to General Procedure III starting from *tert*-butyl 4-methoxypiperidine-1-carboxylate. The crude was purified by preparative thin layer chromatography (SiO_2, ^{n}hexane:EtOAc 2:1) to furnish the product as a colorless oil in 31% yield (20 mg).

^{1}H NMR (500 MHz, $CDCl_3$): δ 7.05 – 6.83 (m, 1H), 5.11 – 4.94 (m, 1H), 3.88 – 3.75 (m, 1H), 3.72 (q, *J* = 4.1 Hz, 1H), 3.35 (s, 3H), 3.34 – 3.29 (m, 1H), 1.96 (br s, 1H), 1.77 – 1.70 (m, 1H), 1.49 (s, 9H). **^{13}C NMR** (126 MHz, $CDCl_3$): δ 128.5, 103.9, 81.3, 69.6, 55.5, 38.5, 37.5, 28.4 (*Note*: The signal corresponding to the Boc carbonyl C is not visible.). **HRMS** (ESI) *m/z*: Calculated for $C_{11}H_{20}NO_3$ $[M+H]^+$ 214.1438, found 214.1437.

tert-Butyl 3-phenyl-3,4-dihydropyridine-1(2H)-carboxylate (25)

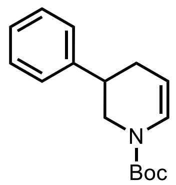

25

Enecarbamate **25** was synthesized according to General Procedure III starting from *tert*-butyl 3-phenylpiperidine-1-carboxylate. The crude was purified by preparative thin layer chromatography (SiO_2, ^{n}hexane:EtOAc 10:1) to furnish the product as a white solid in 24% yield (19 mg).

^{1}H NMR (500 MHz, $CDCl_3$): δ 7.36 – 7.31 (m, 2H), 7.23 – 7.22 (m, 3H), 6.90 (m, 1H), 5.05 – 4.95 (m, 1H), 4.22 – 4.00 (m, 1H), 3.26 – 3.12 (m, 1H), 3.01 – 2.94 (m, 1H), 2.33 – 2.22 (m, 2H), 1.50 – 1.46 (m, 9H). **^{13}C NMR** (126 MHz, $CDCl_3$): δ 152.7, 152.4, 143.6, 143.2, 128.8, 128.7, 127.4, 127.3, 127.0, 126.9, 125.7, 125.4, 105.6, 105.1, 80.9, 48.2, 47.1, 38.9, 38.7, 29.6, 28.5. **HRMS** (ESI) *m/z*: Calculated for $C_{16}H_{21}NNaO_2$ $[M+Na]^+$ 282.1464, found 282.1464.

tert-Butyl 3-((benzoyloxy)methyl)-3,4-dihydropyridine-1(2H)-carboxylate (26)

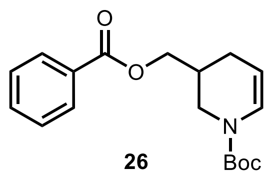

26

Enecarbamate **26** was synthesized according to General Procedure III starting from *tert*-butyl 3-((benzoyloxy)methyl)piperidine-1-carboxylate. The crude was purified by preparative thin layer chromatography (SiO_2, ^{n}hexane:EtOAc 7:1) to furnish the product as a colorless oil in 14% yield (13 mg).

^{1}H NMR (500 MHz, $CDCl_3$): δ 8.05 (d, *J* = 7.6 Hz, 2H), 7.58 – 7.55 (m, 1H), 7.47 – 7.43 (m, 2H), 6.90 – 6.75 (m, 1H), 4.91 – 4.80 (m, 1H), 4.37 – 4.31 (m, 1H), 4.26 – 4.19 (m, 1H), 4.02 – 3.79 (m, 1H), 3.42 – 3.23 (m, 1H), 2.40 – 2.32 (m, 1H), 2.26 – 2.19 (m, 1H), 1.97 – 1.92 (m, 1H), 1.49 – 1.46 (m, 9H). **^{13}C NMR** (126 MHz, $CDCl_3$): δ 166.7, 166.5, 152.8, 152.5, 133.2, 130.2, 130.2, 129.8, 129.7, 128.5, 125.9, 125.6, 103.9, 103.4, 81.0, 80.9, 66.3, 66.0, 44.3, 43.6, 32.00, 31.9, 28.4, 24.7, 24.5. **HRMS** (ESI) *m/z*: Calculated for $C_{18}H_{23}NNaO_4$ $[M+Na]^+$ 340.1519, found 340.1518.

tert-Butyl 2-phenyl-3,4-dihydropyridine-1(2H)-carboxylate (27)

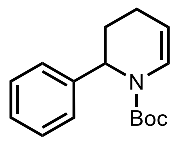

27

Enecarbamate **27** was synthesized according to General Procedure III starting from *tert*-butyl 2-phenylpiperidine-1-carboxylate. The crude was purified by preparative thin layer chromatography (SiO_2, ^{n}hexane:EtOAc 10:1) to furnish the product as a white solid in 14% yield (11 mg).

^{1}H NMR (400 MHz, $CDCl_3$): δ 7.31 – 7.27 (m, 2H), 7.23 – 6.99 (m, 4H), 5.42 – 5.23 (m, 1H), 4.97 – 4.86 (m, 1H), 2.03 – 1.70 (m, 4H), 1.48 – 1.28 (m, 9H). **^{13}C NMR** (126 MHz, $CDCl_3$): δ 153.0, 152.3, 143.0, 141.9, 128.4, 126.8, 125.6, 125.4, 105.7, 105.4, 81.0, 80.7, 55.3, 53.7, 28.5, 28.2, 27.8, 27.7, 17.4, 17.2. **HRMS** (ESI) *m/z*: Calculated for $C_{16}H_{21}NNaO_2$ $[M+Na]^+$ 282.1464, found 282.1472.

The analytical data is consistent with those previously reported.[11]

tert-Butyl 2-((benzoyloxy)methyl)-3,4-dihydropyridine-1(2H)-carboxylate (28)

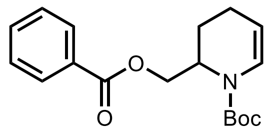

28

Enecarbamate **28** was synthesized according to General Procedure III starting from *tert*-butyl 2-((benzoyloxy)methyl)piperidine-1-carboxylate. The crude was purified by preparative thin layer chromatography (SiO_2, ^{n}hexane:EtOAc 7:1) to furnish the product as a colorless oil in 20% yield (19 mg).

^{1}H NMR (500 MHz, $CDCl_3$): δ 8.06 – 8.04 (m, 2H), 7.58 – 7.52 (m, 1H), 7.47 – 7.40 (m, 2H), 6.89 – 6.72 (m, 1H), 4.97 – 4.62 (m, 2H), 4.36 – 4.28 (m, 2H), 2.16 – 2.08 (m, 1H), 2.05 – 2.01 (m, 2H), 1.88 – 1.80 (m, 1H), 1.51 – 1.39 (m, 9H). **^{13}C NMR** (126 MHz, $CDCl_3$): δ 166.6, 166.5, 152.6, 152.2, 133.3, 133.1, 130.2, 129.9, 128.5, 128.4, 124.5, 124.1, 105.3, 104.5, 81.1, 81.0, 63.1, 49.3, 48.0, 28.4, 28.3, 22.6, 22.4, 18.0, 17.8. **HRMS** (ESI) *m/z*: Calculated for $C_{18}H_{23}NNaO_4$ $[M+Na]^+$ 340.1519, found 340.1513.

tert-Butyl 2,3-dihydro-4H-1,4-oxazine-4-carboxylate (29)

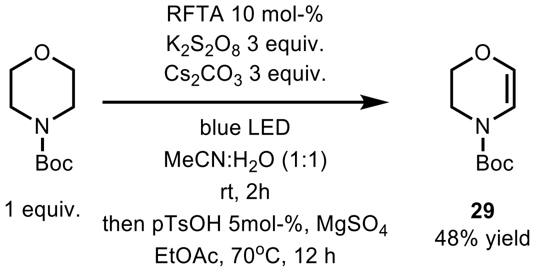

tert-Butyl morpholine-4-carboxylate (1.0 eq., 0.3 mmol), $K_2S_2O_8$ (3.0 eq., 0.9 mmol, 243 mg), Cs_2CO_3 (3.0 eq., 0.9 mmol, 293 mg), MeCN:H_2O (1:1, 15 mL) and RFTA (5 mol%, 8.2 mg) were placed in an 8-dram vial equipped with a stirring bar and the mixture was sonicated until no solid was visible. The solution was irradiated for 1 h under stirring. Afterwards, more RFTA (5 mol%, 8.2 mg) was added, the mixture was briefly sonicated and further irradiated for an hour. Brine was added (5 mL) and the mixture was extracted with EtOAc (3x5 mL). The combined organic layers were dried over Na_2SO_4, filtered, and concentrated under a stream of N_2. The crude product was dissolved in dry EtOAc (20 mM), pTsOH (0.05 equiv., 0.015 mmol) and $MgSO_4$ was added. The suspension was heated at 70 °C under stirring for 12 h, subsequently filtered, and concentrated under a mild N_2 stream. The crude was purified by preparative thin layer chromatography (SiO_2, ^{n}hexane:EtOAc 4:1, extraction from stationary phase: d_3-ACN) to furnish the product as a colorless oil in 48% yield (27 mg).

^{1}H NMR (500 MHz, CD_3CN): δ 6.12 – 6.04 (m, 1H), 5.89 – 5.77 (m, 1H), 3.90 (m, 2H), 3.52 – 3.49 (m, 2H), 1.35 (s, 9H). **^{13}C NMR** (126 MHz, CD_3CN) δ 150.0, 128.4, 127.0, 105.3, 104.9, 79.4, 63.6, 63.3, 41.3, 39.8, 26.5.
The analytical data is consistent with those previously reported.[Error! Unknown switch argument.]

tert-Butyl (R)-2-(((S)-1-methoxy-3-methyl-1-oxobutan-2-yl)carbamoyl)-3,4-dihydropyridine-1(2H)-carboxylate (42)

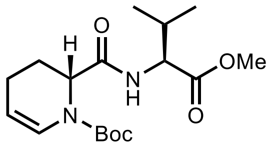

42

Hemiaminal **40** (0.3 mmol, 1 equiv., 107.5 mg) was dissolved in dry EtOAc (15 mL, 0.02 M). $MgSO_4$ was added, and the suspension was stirred for 1 week at 65 °C. The reaction mixture was filtered to remove the $MgSO_4$ and all volatiles were removed under reduced pressure to afford the product as a white solid in quantitative yield without further purification (102 mg).

Note: Application of General procedure III to piperidine **39** led to the formation of **42** in 27% yield, as determined by NMR spectroscopy using $C_2H_2Cl_4$ as internal standard. However, separation of starting material **39** from product **42** by flash column or preparative thin layer chromatography was not possible, which is why the alternative protocol described above was applied.

^{1}H NMR (500 MHz, $CDCl_3$): δ 6.91 (apparent d, 1H), 6.36 (apparent d, 1H), 5.03 (apparent d, 1H), 4.78 (apparent d, 1H), 4.54 (dd, J = 8.9, 4.6 Hz, 1H), 3.71 (s, 3H), 2.39 – 2.32 (m, 1H), 2.23 – 1.95 (m, 3H), 1.77 (br s, 1H), 1.49 (apparent d, 9H), 0.93 (d, J = 6.5 Hz, 3H), 0.84 (d, J = 6.9 Hz, 3H). **^{13}C NMR** (126 MHz, $CDCl_3$): δ 172.4, 172.2, 171.6, 171.1, 152.6, 152.2, 123.7, 123.4, 107.3, 106.8, 82.0, 56.9, 56.3, 54.4, 52.3, 31.2, 28.4, 23.6, 23.0, 19.3, 19.1, 18.44, 18.21, 17.49. **HRMS** (ESI) m/z: Calculated for $C_{18}H_{23}NNaO_4$ $[M+Na]^+$ 340.1519, found 340.1513.

5. Product Derivatization

5.1 Derivatization of Hemiaminals

***tert*-Butyl 2-(1-methoxy-2-methyl-1-oxopropan-2-yl)piperidine-1-carboxylate (30)**

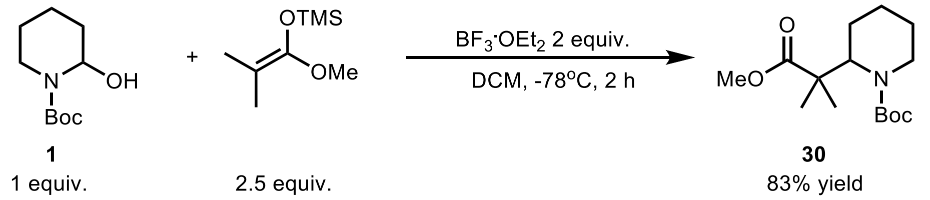

$BF_3·OEt_2$ (2.0 equiv., 61 µL) was added dropwise to a solution of hemiaminal **1** (1.0 equiv., 0.25 mmol, 50 mg) and ((1-methoxy-2-methylprop-1-en-1-yl)oxy)trimethylsilane (2.5 equiv., 126 µL) in CH_2Cl_2 (0.65 mL) at -78°C. The solution was stirred at -78°C for 2 h, then quenched with saturated aqueous NH_4Cl and extracted with CH_2Cl_2 (3x3 mL). The combined organic layers were dried over Na_2SO_4, filtered, and concentrated under a stream of N_2. Purification by preparative thin layer chromatography (SiO_2, ^{n}hexane:EtOAc 6:1) furnished the product as a colorless oil in 83% yield (59 mg).

^{1}H NMR (400 MHz, $CDCl_3$): δ 4.30 (br s, 1H), 4.08 - 3.92 (br d, *J* = 79 Hz, 1H), 3.68 (s, 3H), 2.86 (br s, 1H), 1.72 – 1.65 (m, 1H), 1.60 - 1.47 (m, 5H), 1.45 (s, 9H), 1.22 (s, 3H), 1.20 (s, 3H). **^{13}C NMR** (101 MHz, $CDCl_3$): δ 177.7, 156.4, 79.7, 57.3, 52.0, 47.4 , 40.3, 39.4, 28.5, 24.6, 23.6, 22.4, 19.5.

The analytical data is consistent with those previously reported.[12]

***tert*-Butyl (*Z*)-2-((3,7-dimethylocta-2,6-dien-1-yl)oxy)piperidine-1-carboxylate (31)**

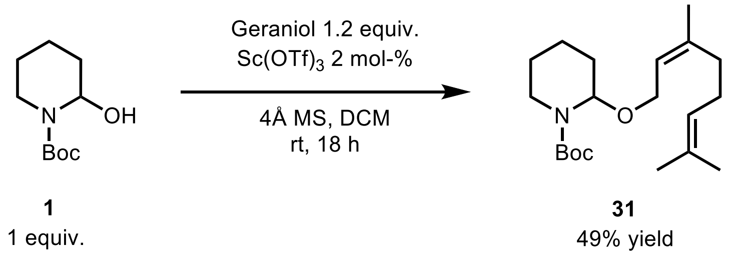

We used a modified literature procedure.[13] $Sc(OTf)_3$ (0.02 equiv., 1.22 mg) was added to a solution of hemiaminal **1** (1.0 equiv., 0.13 mmol, 25 mg) and geraniol (1.2 equiv., 27 µL) in CH_2Cl_2 (0.62 mL) with 4Å MS (1 bead). The resulting mixture was stirred overnight at room temperature. Afterwards, the mixture was diluted with saturated aqueous $NaHCO_3$, extracted three times with CH_2Cl_2, dried over Na_2SO_4, filtered, and the organic layer was concentrated under a stream of N_2. Purification by preparative thin layer chromatography (SiO_2, ^{n}hexane:EtOAc 4:1) furnished the product as a colorless oil 49% yield (17 mg).

^{1}H NMR (500 MHz, $CDCl_3$): δ 5.53-5.40 (br d, *J*=64.8Hz,1H),5.37(tq, *J*=6.7, 1.4 Hz, 1H), 5.12 (tdt, *J* = 7.0, 2.9, 1.4 Hz, 1H), 3.98 - 3.92 (m, 2H), 3.91 - 3.80 (m, 1H), 3.00 - 2.91 (m, 1H), 2.14 - 2.09 (m, 2H), 2.06 - 2.03 (m, 2H), 1.88 - 1.77 (m, 2H), 1.70 (d, *J* = 1.1 Hz, 3H), 1.67 (s, 3H), 1.64 - 1.54 (m, 4H), 1.62 (s, 3H), 1.50 (s, 9H). **^{13}C NMR** (126 MHz, $CDCl_3$): δ 154.7, 139.7, 131.6, 124.1, 120.8, 80.6, 79.8, 63.2 (63.6), 39.6, 38.1 (39.3), 30.5 (30.4), 28.5, 26.4, 25.7, 25.1, 18.6, 17.7, 16.5. Values in brackets relate to the *cis*-isomer. **HRMS** (ESI) *m/z*: Calculated for $C_{20}H_{35}NNaO_3$ $[M+Na]^+$ 360.2515, found 360.2506.

tert-Butyl 2-(Furan-2-yl)piperidine-1-carboxylate (32)

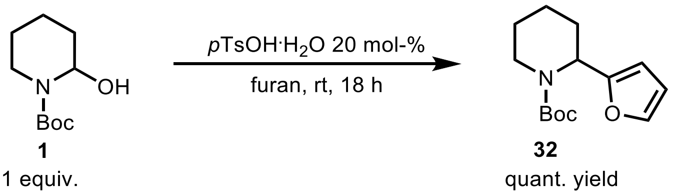

We adapted a known procedure.[14] pTsOH·H_2O (0.20 equiv., 5.7 mg) was added to a solution of hemiaminal **1** (1.0 equiv., 0.15 mmol, 30 mg) in furan (0.32 mL) and the solution stirred at room temperature overnight. Afterwards, water (2 mL) was added, and the mixture was extracted with furan (3x1 mL) and EtOAc (1x1 mL). The combined organic layers were dried over Na_2SO_4, filtered, and concentrated under a stream of N_2, which furnished the product as an amorphous solid in quantitative yield (38 mg).

^{1}H NMR (400 MHz, $CDCl_3$): δ 7.32 – 7.31 (m, 1H), 6.30 (dd, *J* = 3.2, 1.8 Hz, 1H), 6.06 (dt, *J* = 3.2, 1.1 Hz, 1H), 5.37 (s, 1H), 3.97 (d, *J* = 13.7 Hz, 1H), 2.76 (td, *J* = 12.9, 3.0 Hz, 1H), 2.19 – 2.09 (m, 1H), 1.81 – 1.71 (m, 1H), 1.69 – 1.50 (m, 4H), 1.45 (s, 9H). **^{13}C NMR** (101 MHz, $CDCl_3$): δ 155.4, 154.3, 141.5, 110.2, 106.6, 79.8, 49.3, 40.4, 28.5, 27.4, 25.4, 20.1.

The analytical data is consistent with those previously reported.[15]

tert-Butyl 3,4-Dihydropyridine-1(2*H*)-carboxylate (2)

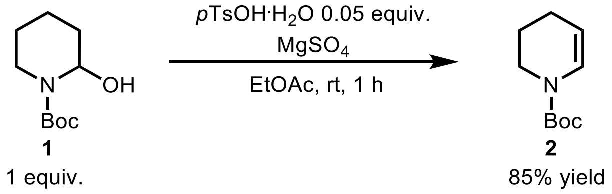

$MgSO_4$ (100 mg) and TsOH·H_2O (0.05 equiv., 1.4 mg) were added to a solution of hemiaminal **1** (1.0 equiv., 0.15 mmol, 30.0 mg) in EtOAc (15 mL). The resulting suspension was stirred at room temperature for 2.5 h, was subsequently filtered and concentrated under a stream of N_2 to furnish the product as a colorless oil in 85% yield (23.3 mg).

^{1}H NMR (400 MHz, $CDCl_3$): δ 6.89 – 6.63 (m, 1H), 4.94 – 4.71 (m, 1H), 3.57-3.49 (m, 2H), 2.01 (tdd, *J* = 6.1, 3.9, 2.0 Hz, 2H), 1.83-1.75 (m, 2H), 1.47 (s, 9H). **^{13}C NMR** (101 MHz, $CDCl_3$): δ 152.5 (152.9), 125.8 (125.4), 105.3 (105.8), 80.6 (80.4), 41.6 (42.7), 28.5 (28.6), 21.9, 21.6. Values in brackets relate to the *cis*-isomer.

The analytical data is consistent with those previously reported.[Error! Unknown switch argument.]
Note: At higher concentration (0.15 M), the dimerization product **44** was the main product.

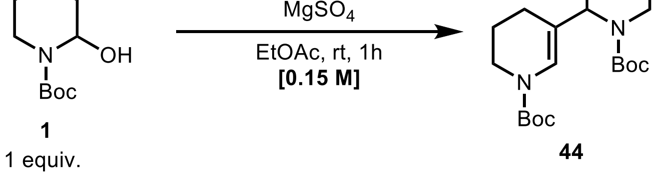

^{1}H NMR (400 MHz, $CDCl_3$): δ 6.81 – 6.53 (m, 1H), 4.71 (d, *J* = 17.0 Hz, 1H), 3.96 – 3.87 (m, 1H), 3.64 – 3.34 (m, 2H), 2.78 – 2.59 (m, 1H), 1.96 – 1.71 (m, 5H), 1.66 – 1.49 (m, 5H), 1.47 (s, 9H).

***tert*-Butyl 2-cyanopiperidine-1-carboxylate (33)**

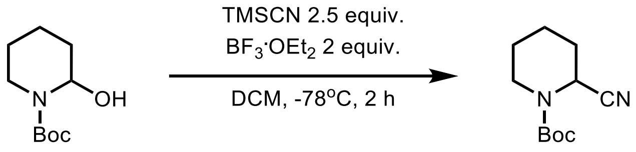

1 1 equiv. **33** 72% yield

$BF_3·OEt_2$ (2.0 equiv., 61 µL) was added dropwise to a solution of hemiaminal **1** (1.0 equiv., 0.25 mmol, 50 mg) and TMSCN (2.5 equiv., 77 µL) in CH_2Cl_2 (0.65 mL) at -78°C. The solution was stirred at -78°C for 2 h. The reaction was then quenched with saturated aqueous NH_4Cl and the aqueous layer was extracted with CH_2Cl_2 (3x3 mL). The combined organic layers were dried over Na_2SO_4, filtered, and concentrated under a stream of N_2. Purification by preparative thin layer chromatography (SiO_2, ^{n}hexane:EtOAc 5:1) furnished the product in 72% yield (37 mg).

^{1}H NMR (400 MHz, $CDCl_3$): δ 5.23 (br s, 1H), 4.04 (br s, 1H), 2.94 (br s, 1H), 1.97 - 1.91 (m, 1H), 1.86 – 1.78 (m, 1H), 1.71 (m, 3H), 1.48 (s, 9H), 1.45 - 1.37 (m, 1H). **^{13}C NMR** (101 MHz, $CDCl_3$): δ 117.9, 81.6, 44.1, 41.8, 28.6, 28.4, 24.7, 20.5 (*Note*: The signal corresponding to the Boc carbonyl C is not visible.).

The analytical data is consistent with those previously reported.[16]

3-((Trimethylsilyl)methyl)hexahydro-1*H*,3*H*-pyrido[1,2-*c*][1,3]oxazin-1-one (34)

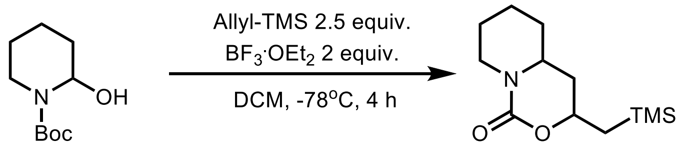

1 1 equiv. **34** 78% yield

$BF_3·OEt_2$ (2.0 equiv., 61 µL) was added dropwise to a solution of hemiaminal **1** (1.0 equiv., 0.25 mmol, 50 mg) and allyl-TMS (2.5 equiv., 100 µL) in CH_2Cl_2 (0.65 mL) at -78°C. The solution was stirred at -78°C for 4h, after which the reaction was quenched with saturated aqueous NH_4Cl and extracted with CH_2Cl_2 (3x3 mL). The combined organic layers were dried over Na_2SO_4, filtered, and concentrated under a stream of N_2. The crude was purified by preparative thin layer chromatography (SiO_2, ^{n}hexane:EtOAc 6:1) to furnish a mixture of diastereoisomers as a white solid with a d.r. = 3:1 and a 78% overall yield (47 mg).

^{1}H NMR (500 MHz, $CDCl_3$): δ 4.50 – 4.33 (m, 2H), 4.25 (dddd, *J* = 11.4, 8.2, 6.4, 1.9 Hz, 1H), 4.12 (q, *J* = 7.2 Hz, 1H), 3.26 (tdd, *J* = 11.2, 5.6, 2.7 Hz, 2H), 2.71 – 2.59 (m, 2H), 2.06 (ddd, *J* = 13.8, 5.6, 1.9 Hz, 1H), 2.04 (s, 1H), 1.99 – 1.87 (m, 1H), 1.86 – 1.64 (m, 4H), 1.64 – 1.31 (m, 6H), 1.26 (t, *J* = 7.2 Hz, 1H), 1.22 – 1.09 (m, 3H), 0.90 (ddd, *J* = 14.5, 8.1, 4.2 Hz, 2H), 0.07 (d, *J* = 1.6 Hz, 9H). **^{13}C NMR** (126 MHz, $CDCl_3$): 154.0 (*major*), 153.9 (*minor*), 73.8 (*major*), 72.4 (*minor*), 54.2 (*major*), 53.1 (*minor*), 45.6 (*minor*), 44.7 (*major*), 38.9 (*major*), 36.0 (*minor*), 33.7 (*major*), 33.3 (*minor*), 25.6 (*minor*), 25.1 (*major*), 24.6 (*minor*), 24.2 (*major*), 23.8 (*major*), 23.6 (*minor*), -0.74 (*major*), -0.81 (*minor*). **HRMS** (ESI) *m/z*: Calculated for $C_{12}H_{24}NO_2Si$ $[M+H]^+$ 242.1571, found 242.1571. **R_f** (^{n}hexane:EtOAc 6:1) = 0.11.

***tert*-Butyl-(6*R*)-2-(1-methoxy-2-methyl-1-oxopropan-2-yl)-6-(((*S*)-1-methoxy-3-methyl-1-oxobutan-2-yl)carbamoyl)piperidine-1-carboxylate (41)**

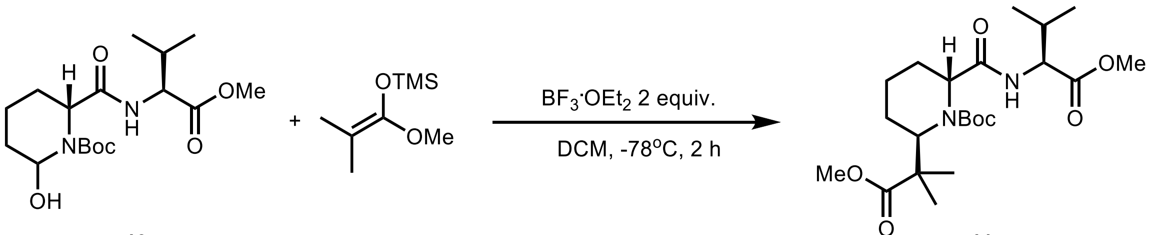

40 1 equiv. | 2.5 equiv. | **41** 80% yield

BF_3·OEt_2 (2.0 equiv., 61 µL) was added dropwise to a solution of hemiaminal **40** (1.0 equiv., 0.25 mmol, 50 mg) and ((1-methoxy-2-methylprop-1-en-1-yl)oxy)trimethylsilane (2.5 equiv., 126 µL) in CH_2Cl_2 (0.65 mL) at -78°C. The solution was stirred at -78°C for 2h, after which the reaction was quenched with saturated aqueous NH_4Cl. The phases were separated and the aqueous layer was extracted with CH_2Cl_2 (3x3 mL). The combined organic layers were dried over Na_2SO_4, filtered, and concentrated under a stream of N_2. Purification by preparative thin layer chromatography (SiO_2, ^{n}hexane:EtOAc 6:1) furnished the product as a white solid in 80% yield (89 mg).

^{1}H NMR (500 MHz, $CDCl_3$): δ 7.23 (br s, 1H), 4.55 (dd, *J* = 8.8, 5.4 Hz, 1H), 4.02 (dd, *J* = 11.0, 6.0 Hz, 1H), 3.80 (br s, 1H), 3.72 (s, 3H), 3.70 (s, 3H), 2.25 – 2.14 (m, 2H), 1.80 – 1.62 (m, 3H), 1.45 – 1.39 (m, 1H), 1.37 (s, 9H), 1.31 (apparent d, 6H), 1.24 – 1.13 (m, 1H), 0.96 (apparent d, 6H). **^{13}C NMR** (126 MHz, $CDCl_3$): δ 177.3, 172.6, 171.1, 156.5, 81.2, 63.1, 57.4, 55.6, 52.2, 52.0, 46.3, 31.8, 28.2, 24.7, 22.8, 22.7, 21.9, 19.4, 19.2, 18.0. **HRMS** (ESI) *m/z*: Calculated for $C_{22}H_{38}N_2NaO_7$ $[M+Na]^+$ 465.2571, found 465.2571.

5.2 Derivatization of Enecarbamates

***tert*-Butyl 3-fluoropiperidine-1-carboxylate (35)**

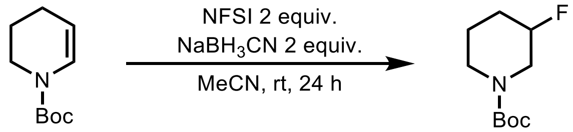

2 1 equiv. | **35** 52% yield

We followed a literature procedure.[17] A reaction vial was charged with a solution of encarbamate **2** (0.3 mmol, 1 equiv., 55.0 mg) in dry MeCN (50mM, 6 mL) under a N_2 atmosphere. 1.0 M $NaBH_3CN$ in THF (0.6 mmol, 2 equiv., 600 µL) and NFSI (0.6 mmol, 2 equiv., 105 mg) were added. The reaction mixture was stirred at room temperature for 24 h. Complete consumption of the starting material was corroborated by TLC. The solvent was evaporated under reduced pressure and the crude was purified by preparative thin layer chromatography (^{n}hexane:EtOAc 3:1) to furnish the product as a white solid in 52% yield (32 mg).

^{1}H NMR (400 MHz, $CDCl_3$): δ 4.57 (dp, *J* = 47.0, 4.45 Hz, 1H), 3.57 – 3.45 (m, 3H), 3.29 – 3.24 (m, 1H), 1.91 – 1.74 (m, 3H), 1.47 – 1.44 (m, 10H). **^{13}C NMR** (101 MHz, $CDCl_3$): δ 155.1, 86.6 (d, *J* = 174.7 Hz), 79.9, 48.3, 43.3, 3 (d, *J* = 20.4 Hz), 28.5, 21.4. **^{19}F NMR** (376 MHz, $CDCl_3$): δ -184.41.

The analytical data is consistent with those previously reported.[18]

tert-Butyl 5-(2,2,2-trifluoroacetyl)-3,4-dihydropyridine-1(2*H*)-carboxylate (36)

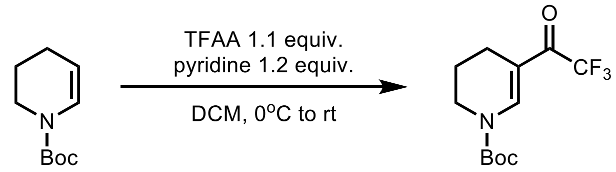

2 1 equiv. **36** 93% yield

Following a procedure from literature,[Error! Unknown switch argument.] tert-butyl 3,4-dihydropyridine-1(2H)-carboxylate (1 equiv., 100 mg) and dry pyridine (1.2 equiv., 52.8 µL) were dissolved in dry CH_2Cl_2 (1.8 mL) and the mixture cooled to 0°C. Trifluoroacetic anhydride (1.1 equiv., 84.6 µL) was added dropwise, and the solution was stirred from 30 min at 0 °C. Afterwards, the mixture was warmed to rt and stirred for 2 h. The mixture was cooled to 0 °C, the reaction was quenched with sat. aq. $NaHCO_3$ and the mixture was stirred for 15 min. The pH was adjusted to pH 2 with 3N HCl and extracted with CH_2Cl_2. The combined organic layers were dried over Na_2SO_4, filtered and concentrated under a stream of N_2 to yield the title compound as a pale yellow oil in 93% yield (142 mg).

^{1}H NMR (400 MHz, $CDCl_3$): δ 8.30 (s, 1H), 3.67 – 3.59 (m, 2H), 2.40 – 2.32 (t, *J* = 6.3 Hz, 2H), 1.93 – 1.83 (m, 2H), 1.54 (s, 9H). **^{13}C NMR** (101 MHz, $CDCl_3$): δ 179.1, 151.4, 143.1 (q, *J* = 5.0 Hz), 117.2 (q, *J* = 291.8 Hz), 111.2, 84.0, 42.8, 28.1, 20.2, 19.7. **^{19}F NMR** (376 MHz, $CDCl_3$): δ -69.2.

The analytical data is consistent with those previously reported.[Error! Unknown switch argument.]

tert-Butyl 5-formyl-3,4-dihydropyridine-1(2*H*)-carboxylate (37)

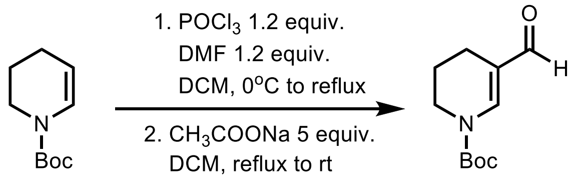

2 1 equiv. **37** 15% yield

According to a known procedure,[19] phosphorus oxychloride (0.66 mmol) was added dropwise to DMF (0.66 mmol) at 10–20 °C during 3 min, and the mixture was stirred for 20 min. Once cooled to 0 °C, CH_2Cl_2 (0.78 mL) was added. Next, over a 30-min period, a solution of the enecarbamate **2** (0.55 mmol) in CH_2Cl_2 (0.39 mL) was added dropwise. The reaction mixture was stirred at 0 °C for 1 h and was then refluxed for 15 min. Subsequently, a solution of sodium acetate trihydrate (412 mg, 3.03 mmol) in water (2.33 mL) was added to the cooled mixture. After refluxing for an additional 15 min, the reaction was allowed to warm to room temperature. The organic phase was separated, and the aqueous layer was extracted with CH_2Cl_2 (6 x 2 mL). The combined organic layers were dried over anhydrous sodium sulfate. The crude reaction mixture was purified by column chromatography on silica gel (EtOAc:^{n}hexane 5:1 to 1:1) to furnish the product in 15% yield (17 mg).

^{1}H NMR (400 MHz, $CDCl_3$): δ 9.23 (s, 1H), 7.69 (br s, 1H), 3.62 (t, *J* = 5.8 Hz, 2H), 2.26 (t, *J* = 6.2 Hz, 2H), 1.82 (p, *J* = 6.1 Hz, 2H), 1.53 (s, 9H). **^{13}C NMR** (101 MHz, $CDCl_3$): δ 190.6, 151.3, 145.6, 120.2, 83.3, 43.2, 28.2, 20.2, 18.3.

The spectroscopic data is consistent with those previously reported.[Error! Unknown switch argument.]

tert-Butyl-3-(pyridin-3-yl)piperidine-1-carboxylate (38)

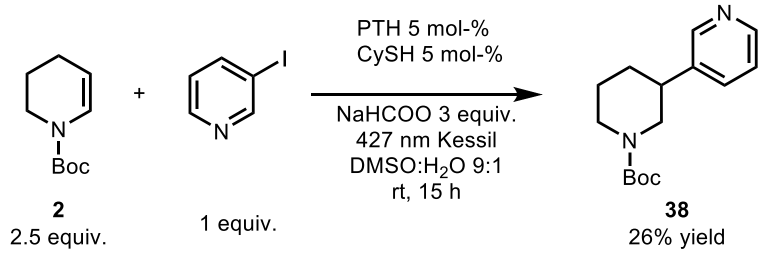

According to a literature procedure,[20] 3-iodopyridine (1 equiv., 0.5 mmol, 102.5 mg), sodium formate (3 equiv., 102.0 mg) and 10-phenylphenothiazine (5 mol-%, 6.9 mg) were placed in a 2-dram vial equipped with a stirring bar. Under an Ar-atmosphere, DMSO:H_2O (20:1 *v*/*v*, 5 mL, sparged for 40 min with Ar), enecarbamate **2** (2.5 equiv., 232 µL) and cyclohexyl thiol (5 mol-%, 3 µL) were added. The mixture was irradiated at 427 nm for 15 h with cooling from a household fan (*Note:* This reaction was not performed in the *ETHos* photosetup**Error! Unknown switch argument.**) and then quenched which sat. aq. $NaHCO_3$ (15 mL). The mixture extracted with EtOAc (4x15 mL) and the combined organic layers were dried over Na_2SO_4, filtered and concentrated under reduced pressure. Flash-column chromatography (SiO_2, 4:1 → 1:1 → 1:3 → 0:100 ^{n}hex:EtOAc) yielded the title compound as a pale yellow oil in 26% yield (35 mg).

^{1}H NMR (400 MHz, $CDCl_3$): δ 8.54 – 8.45 (m, 2H), 7.54 (dt, *J* = 7.9, 2.0 Hz, 1H), 7.25 (dd, *J* = 7.9, 4.8 Hz, 1H), 4.30 – 4.06 (m, 2H), 2.91 – 2.61 (m, 3H), 2.08 – 1.96 (m, 1H), 1.82 – 1.74 (m, 1H), 1.71 – 1.56 (m, 2H), 1.47 (s, 9H). **^{13}C NMR** (101 MHz, $CDCl_3$): δ 154.8, 149.1, 148.2, 138.8, 134.6, 123.5, 79.8, 50.0, 44.3, 40.2, 31.6, 28.6, 28.4 (2C), 25.3.

The analytical data is consistent with those previously reported.[21]

tert-Butyl (2*R*)-5-fluoro-2-(((*S*)-1-methoxy-3-methyl-1-oxobutan-2-yl)carbamoyl)piperidine-1-carboxylate (43)

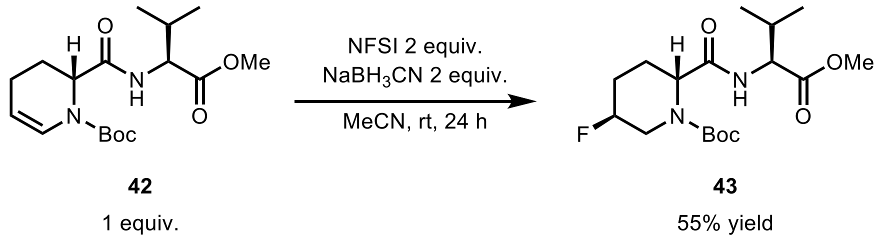

According to a literature-known procedure,**Error! Unknown switch argument.** a 1-dram reaction vial was charged with a solution of enecarbamate **42** (28.5 µmol, 1 equiv., 9.70 mg) in dry MeCN (50 mM, 0.57 mL) under a N_2 atmosphere. 1.0 M $NaBH_3CN$ in THF (57.0 µmol, 2 equiv., 57.0 µL) and NFSI (57.0 µmol, 2 equiv., 10 mg) were added. The reaction mixture was stirred at room temperature for 24 h, and complete consumption of the starting material was corroborated by TLC. The solvent was evaporated under reduced pressure and the crude was directly purified by preparative thin layer chromatography (^{n}hexane:EtOAc 1:1) to furnish the product as a white solid in 55% yield (5.3 mg).

^{1}H NMR (600 MHz, $CDCl_3$): δ 6.68-6.36 (m, 1H), 4.92 – 4.65 (m, 2H), 4.54 – 4.49 (m, 1H), 4.33 – 4.28 (m, 1H), 3.73 (s, 3H), 3.10 (dd, *J* = 41.3, 14.7 Hz, 1H), 2.25 – 2.11 (m, 2H), 1.97 – 1.81 (m, 3H), 1.50 (s, 9H), 0.95 (d, *J* = 6.9 Hz, 3H), 0.88 (d, *J* = 7.0 Hz, 3H). **^{13}C NMR** (151 MHz, $CDCl_3$):

δ 172.6, 172.4, 171.3, 170.9, 156.8, 155.0, 85.6 (d, *J* = 174.3 Hz), 85.4 (d, *J* = 176.37), 81.4, 57.2, 55.0, 52.8, 52.3, 45.7 (d, *J* = 20.8 Hz), 44.4 (d, *J* = 22.9 Hz), 30.9, 30.7, 28.4, 25.2 (d, *J* = 21.3 Hz), 19.5, 19.3, 18.9, 17.61. **^{19}F NMR** (471 MHz, $CDCl_3$): δ -188.47 – -188.99 (m), -189.36 (apparent q, *J* = 42.3 Hz). **HRMS** (ESI) *m/z*: Calculated for $C_{17}H_{29}FN_2NaO_5$ $[M+Na]^+$ 383.1953, found 383. 1952.

Crystal data and structure

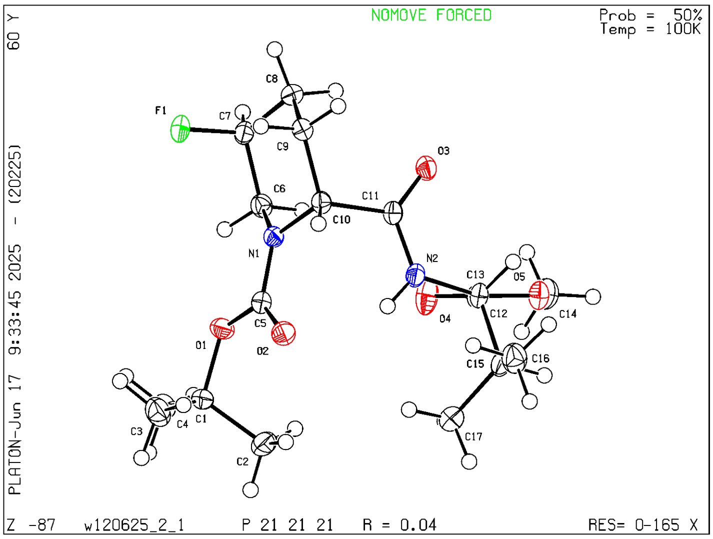

Crystals were obtained by slow evaporation of a solution in $CHCl_3$. A clear colorless, needle-shaped crystal was mounted on the goniometer. Data for **43** were collected from a shock-cooled single crystal at 100.0(1) K on a XtaLAB Synergy R, HyPix-Arc 150 four-circle diffractometer with a Rotating-anode X-ray tube using a mirror as monochromator and a HyPix-Arc 150 detector. The diffractometer used Cu K_α radiation (λ = 1.54184 Å). All data were integrated with CrysAlisPro and a gaussian absorption correction using SCALE3 ABSPACK was applied.[22] The structure was solved by dual methods with SHELXT 2018/2 and refined by full-matrix least-squares methods against F^2 using SHELXL 2019/3.[23,24] All non-hydrogen atoms were refined with anisotropic displacement parameters. All hydrogen atoms were refined with isotropic displacement parameters. Some of their coordinates were refined freely and some on calculated positions using a riding model with their U_{iso} values constrained to 1.5 times the U_{eq} of their pivot atoms for terminal sp^3 carbon atoms and 1.2 times for all other carbon atoms. Crystallographic data for the structures reported in this paper have been deposited with the Cambridge Crystallographic Data Centre.[25] CCDC 2464910 contain the supplementary crystallographic data for this paper. These data can be obtained free of charge from The Cambridge Crystallographic Data Centre via www.ccdc.cam.ac.uk/structures. This report and the CIF file were generated using FinalCif.[26]

Table ***S8:*** *Crystal data and structure refinement for* **43**.

CCDC number	2464910	Index ranges	−6 ≤ h ≤ 7<br>−11 ≤ k ≤ 11<br>−38 ≤ l ≤ 38
Empirical formula	$C_{17}H_{29}FN_2O_5$	Reflections collected	32295
Formula weight	360.42	Independent reflections	3756<br>R_{int} = 0.0671<br>R_{sigma} = 0.0384
Temperature [K]	100.0(1)	Completeness to θ = 67.684°	99.9 %
Crystal system	orthorhombic	Data / Restraints / Parameters	3756 / 1 / 235
Space group (number)	$P2_12_12_1$ (19)	Absorption correction T_{min}/T_{max} (method)	0.8930 / 1.0000 (gaussian)
a [Å]	6.43950(10)	Goodness-of-fit on F^2	1.107
b [Å]	9.5019(2)	Final *R* indexes [$I≥2σ(I)$]	R_1 = 0.0396<br>wR_2 = 0.0953
c [Å]	30.8275(8)	Final *R* indexes [all data]	R_1 = 0.0509<br>wR_2 = 0.1040
α [°]	90	Largest peak/hole [eÅ^{-3}]	0.21/−0.30
β [°]	90	Flack X parameter	0.00(9)
γ [°]	90		
Volume [Å^3]	1886.26(7)		
Z	4		
ρ_{calc} [gcm^{-3}]	1.269		
μ [mm^{-1}]	0.829		
F(000)	776		
Crystal size [mm^3]	0.016×0.033×0.128		
Crystal colour	clear colourless		
Crystal shape	needle		
Radiation	Cu K_α (λ=1.54184 Å)		
2θ range [°]	5.73 to 149.59 (0.80 Å)		

Table *S9:* Atomic coordinates and U_{eq} [$Å^2$] for **43**.

**Atom**	***x***	***y***	***z***	U_{eq}
F1	0.6022(3)	−0.04148(15)	0.43675(5)	0.0294(4)
O1	0.2714(3)	0.1584(2)	0.34117(6)	0.0244(4)
O2	0.5266(3)	0.31085(19)	0.32059(6)	0.0249(4)
O3	0.6181(3)	0.48145(19)	0.45833(6)	0.0245(4)
O4	0.1371(3)	0.5294(2)	0.42340(8)	0.0380(6)
O5	0.1738(3)	0.75623(19)	0.44303(6)	0.0256(4)
N1	0.5178(3)	0.2178(2)	0.38882(7)	0.0186(5)
N2	0.5383(4)	0.5322(2)	0.38859(8)	0.0228(5)
H2	0.532(5)	0.499(3)	0.3614(8)	0.027
C1	0.1626(5)	0.1608(3)	0.29921(9)	0.0244(6)
C2	0.0971(5)	0.3104(3)	0.28780(11)	0.0345(7)
H2A	0.031381	0.354339	0.313081	0.052
H2B	−0.001865	0.307981	0.263659	0.052
H2C	0.219551	0.365109	0.279333	0.052
C3	0.2998(5)	0.0953(3)	0.26461(10)	0.0342(8)
H3A	0.423510	0.153802	0.260397	0.051
H3B	0.222579	0.088974	0.237289	0.051
H3C	0.341948	0.000781	0.273816	0.051
C4	−0.0259(5)	0.0703(3)	0.30826(10)	0.0269(6)
H4A	0.019055	−0.024580	0.316474	0.040
H4B	−0.112392	0.065022	0.282149	0.040
H4C	−0.106448	0.111775	0.332005	0.040
C5	0.4446(4)	0.2350(3)	0.34779(9)	0.0209(6)
C6	0.3910(4)	0.1571(3)	0.42352(9)	0.0219(6)
H6A	0.289768	0.090477	0.410755	0.026
H6B	0.311943	0.233095	0.438009	0.026
C7	0.5212(4)	0.0809(3)	0.45679(9)	0.0222(6)
H7	0.431794	0.053041	0.481882	0.027
C8	0.7017(4)	0.1670(3)	0.47293(9)	0.0229(6)
H8A	0.792269	0.107376	0.491237	0.027
H8B	0.649207	0.245279	0.491087	0.027
C9	0.8281(4)	0.2268(3)	0.43539(9)	0.0221(6)
H9A	0.934862	0.291560	0.447037	0.026
H9B	0.900592	0.148867	0.420413	0.026
C10	0.6942(4)	0.3057(3)	0.40273(9)	0.0190(6)
H10	0.781735	0.324868	0.376598	0.023
C11	0.6141(4)	0.4483(3)	0.41974(9)	0.0193(5)
C12	0.4366(4)	0.6642(3)	0.39946(9)	0.0223(6)
H12	0.529618	0.716392	0.419860	0.027
C13	0.2335(5)	0.6382(3)	0.42259(10)	0.0244(6)
C14	−0.0156(5)	0.7491(3)	0.46785(10)	0.0325(7)
H14A	−0.129359	0.717779	0.449078	0.049
H14B	−0.047984	0.842443	0.479566	0.049
H14C	0.001837	0.682274	0.491788	0.049
C15	0.4072(5)	0.7565(3)	0.35879(9)	0.0261(6)

H15	0.333350	0.844021	0.368066	0.031
C16	0.6178(5)	0.8006(3)	0.34057(11)	0.0330(7)
H16A	0.695620	0.851906	0.362845	0.049
H16B	0.596955	0.861493	0.315290	0.049
H16C	0.695934	0.716698	0.331892	0.049
C17	0.2728(5)	0.6849(3)	0.32449(10)	0.0309(7)
H17A	0.343340	0.600190	0.313918	0.046
H17B	0.249780	0.749795	0.300257	0.046
H17C	0.138888	0.658717	0.337274	0.046
H16C	0.695934	0.716698	0.331892	0.049
C17	0.2728(5)	0.6849(3)	0.32449(10)	0.0309(7)
H17A	0.343340	0.600190	0.313918	0.046
H17B	0.249780	0.749795	0.300257	0.046
H17C	0.138888	0.658717	0.337274	0.046

U_{eq} is defined as 1/3 of the trace of the orthogonalized U_{ij} tensor.

Table S10: Anisotropic displacement parameters [Å^2] for **43**. The anisotropic displacement factor exponent takes the form: $-2\pi^2[h^2(a^*)^2U_{11} + k^2(b^*)^2U_{22} + ... + 2hka^*b^*U_{12}]$

**Atom**	U_{11}	U_{22}	U_{33}	U_{23}	U_{13}	U_{12}
F1	0.0415(9)	0.0150(7)	0.0316(9)	−0.0030(7)	−0.0031(8)	0.0017(7)
O1	0.0299(11)	0.0237(10)	0.0196(10)	0.0028(8)	−0.0058(8)	−0.0059(8)
O2	0.0326(10)	0.0222(9)	0.0199(10)	0.0031(8)	0.0013(8)	−0.0034(8)
O3	0.0341(11)	0.0189(9)	0.0204(10)	−0.0029(8)	0.0001(9)	0.0005(8)
O4	0.0376(12)	0.0194(10)	0.0571(15)	−0.0036(10)	0.0096(11)	−0.0064(9)
O5	0.0297(10)	0.0178(9)	0.0293(10)	−0.0039(9)	0.0038(9)	0.0002(8)
N1	0.0224(11)	0.0165(10)	0.0169(11)	0.0003(8)	−0.0003(9)	−0.0009(9)
N2	0.0334(13)	0.0148(10)	0.0203(12)	−0.0011(9)	−0.0001(10)	0.0019(10)
C1	0.0334(15)	0.0202(13)	0.0196(14)	0.0012(11)	−0.0074(12)	−0.0013(11)
C2	0.0399(17)	0.0218(14)	0.0420(19)	0.0063(13)	−0.0161(15)	−0.0025(13)
C3	0.048(2)	0.0300(16)	0.0245(16)	−0.0043(13)	0.0013(15)	−0.0056(14)
C4	0.0313(16)	0.0231(13)	0.0263(15)	−0.0034(12)	−0.0050(13)	−0.0028(12)
C5	0.0247(14)	0.0166(11)	0.0213(14)	−0.0010(11)	−0.0012(11)	0.0010(10)
C6	0.0233(13)	0.0200(12)	0.0223(14)	−0.0006(11)	0.0002(12)	−0.0029(11)
C7	0.0314(15)	0.0153(11)	0.0197(13)	−0.0003(10)	0.0005(12)	0.0010(11)
C8	0.0280(15)	0.0203(13)	0.0204(14)	0.0010(11)	−0.0031(12)	0.0023(11)
C9	0.0247(13)	0.0198(12)	0.0217(14)	−0.0024(11)	−0.0032(12)	0.0008(11)
C10	0.0209(14)	0.0166(12)	0.0195(13)	−0.0011(10)	0.0010(11)	−0.0014(10)
C11	0.0200(13)	0.0161(11)	0.0219(14)	−0.0011(10)	0.0016(11)	−0.0017(10)
C12	0.0269(15)	0.0137(11)	0.0263(15)	−0.0020(11)	−0.0005(12)	0.0005(10)
C13	0.0316(15)	0.0147(12)	0.0270(15)	−0.0013(11)	−0.0042(13)	0.0009(11)
C14	0.0313(16)	0.0290(14)	0.0372(17)	−0.0026(14)	0.0055(14)	0.0023(13)
C15	0.0364(16)	0.0151(12)	0.0270(15)	0.0015(11)	0.0001(13)	0.0004(12)
C16	0.0424(18)	0.0264(14)	0.0300(16)	−0.0003(12)	0.0047(15)	−0.0058(13)
C17	0.0382(17)	0.0238(14)	0.0306(17)	0.0023(13)	−0.0063(14)	0.0012(12)

Table S11: Bond lengths for **43**.

Atom–Atom	Length [Å]
F1–C7	1.416(3)
O1–C1	1.471(3)
O1–C5	1.347(3)
O2–C5	1.225(3)
O3–C11	1.231(3)
O4–C13	1.206(3)
O5–C13	1.343(3)
O5–C14	1.441(3)
N1–C5	1.360(3)
N1–C6	1.464(3)
N1–C10	1.474(3)
N2–H2	0.90(2)
N2–C11	1.341(3)
N2–C12	1.454(3)
C1–C2	1.524(4)
C1–C3	1.519(4)
C1–C4	1.514(4)
C2–H2A	0.9800
C2–H2B	0.9800
C2–H2C	0.9800
C3–H3A	0.9800
C3–H3B	0.9800
C3–H3C	0.9800
C4–H4A	0.9800
C4–H4B	0.9800
C4–H4C	0.9800
C6–H6A	0.9900
C6–H6B	0.9900
C6–C7	1.510(4)
C7–H7	1.0000
C7–C8	1.506(4)
C8–H8A	0.9900
C8–H8B	0.9900
C8–C9	1.525(4)
C9–H9A	0.9900
C9–H9B	0.9900
C9–C10	1.523(4)
C10–H10	1.0000
C10–C11	1.541(3)
C12–H12	1.0000
C12–C13	1.510(4)
C12–C15	1.541(4)
C14–H14A	0.9800
C14–H14B	0.9800
C14–H14C	0.9800
C15–H15	1.0000
C15–C16	1.526(4)
C15–C17	1.526(4)
C16–H16A	0.9800
C16–H16B	0.9800
C16–H16C	0.9800
C17–H17A	0.9800
C17–H17B	0.9800
C17–H17C	0.9800

Table S12: Bond angles for **43**.

Atom–Atom–Atom	Angle [°]
C5–O1–C1	121.3(2)
C13–O5–C14	116.9(2)
C5–N1–C6	122.2(2)
C5–N1–C10	118.0(2)
C6–N1–C10	116.1(2)
C11–N2–H2	118(2)
C11–N2–C12	120.8(2)
C12–N2–H2	120(2)
O1–C1–C2	110.4(2)
O1–C1–C3	109.5(2)
O1–C1–C4	102.2(2)
C3–C1–C2	112.4(3)
C4–C1–C2	110.5(3)
C4–C1–C3	111.3(2)
C1–C2–H2A	109.5
C1–C2–H2B	109.5
C1–C2–H2C	109.5
H2A–C2–H2B	109.5
H2A–C2–H2C	109.5
H2B–C2–H2C	109.5
C1–C3–H3A	109.5

C1–C3–H3B	109.5
C1–C3–H3C	109.5
H3A–C3–H3B	109.5
H3A–C3–H3C	109.5
H3B–C3–H3C	109.5
C1–C4–H4A	109.5
C1–C4–H4B	109.5
C1–C4–H4C	109.5
H4A–C4–H4B	109.5
H4A–C4–H4C	109.5
H4B–C4–H4C	109.5
O1–C5–N1	111.3(2)
O2–C5–O1	124.8(2)
O2–C5–N1	123.9(2)
N1–C6–H6A	109.2
N1–C6–H6B	109.2
N1–C6–C7	112.0(2)
H6A–C6–H6B	107.9
C7–C6–H6A	109.2
C7–C6–H6B	109.2
F1–C7–C6	107.6(2)
F1–C7–H7	109.4
F1–C7–C8	107.8(2)
C6–C7–H7	109.4
C8–C7–C6	113.1(2)
C8–C7–H7	109.4
C7–C8–H8A	109.4
C7–C8–H8B	109.4
C7–C8–C9	111.3(2)
H8A–C8–H8B	108.0
C9–C8–H8A	109.4
C9–C8–H8B	109.4
C8–C9–H9A	109.1
C8–C9–H9B	109.1
H9A–C9–H9B	107.8
C10–C9–C8	112.5(2)
C10–C9–H9A	109.1
C10–C9–H9B	109.1
N1–C10–C9	110.5(2)
N1–C10–H10	107.6

N1–C10–C11	109.8(2)
C9–C10–H10	107.6
C9–C10–C11	113.4(2)
C11–C10–H10	107.6
O3–C11–N2	123.2(2)
O3–C11–C10	123.2(2)
N2–C11–C10	113.6(2)
N2–C12–H12	107.6
N2–C12–C13	111.0(2)
N2–C12–C15	111.0(2)
C13–C12–H12	107.6
C13–C12–C15	111.8(2)
C15–C12–H12	107.6
O4–C13–O5	124.0(3)
O4–C13–C12	126.6(3)
O5–C13–C12	109.4(2)
O5–C14–H14A	109.5
O5–C14–H14B	109.5
O5–C14–H14C	109.5
H14A–C14–H14B	109.5
H14A–C14–H14C	109.5
H14B–C14–H14C	109.5
C12–C15–H15	107.4
C16–C15–C12	110.3(3)
C16–C15–H15	107.4
C16–C15–C17	111.8(2)
C17–C15–C12	112.3(2)
C17–C15–H15	107.4
C15–C16–H16A	109.5
C15–C16–H16B	109.5
C15–C16–H16C	109.5
H16A–C16–H16B	109.5
H16A–C16–H16C	109.5
H16B–C16–H16C	109.5
C15–C17–H17A	109.5
C15–C17–H17B	109.5
C15–C17–H17C	109.5
H17A–C17–H17B	109.5
H17A–C17–H17C	109.5
H17B–C17–H17C	109.5

Table S13: Torsion angles for **43**.

**Atom–Atom–Atom–Atom**	**Torsion Angle [°]**
F1–C7–C8–C9	67.9(3)
N1–C6–C7–F1	−69.2(3)
N1–C6–C7–C8	49.8(3)
N1–C10–C11–O3	109.0(3)
N1–C10–C11–N2	−70.5(3)
N2–C12–C13–O4	−17.3(4)
N2–C12–C13–O5	162.3(2)
N2–C12–C15–C16	−64.9(3)
N2–C12–C15–C17	60.5(3)
C1–O1–C5–O2	−0.4(4)
C1–O1–C5–N1	179.5(2)
C5–O1–C1–C2	−58.3(3)
C5–O1–C1–C3	66.0(3)
C5–O1–C1–C4	−175.9(2)
C5–N1–C6–C7	150.4(2)
C5–N1–C10–C9	−148.6(2)
C5–N1–C10–C11	85.5(3)
C6–N1–C5–O1	−16.0(3)
C6–N1–C5–O2	163.9(2)
C6–N1–C10–C9	52.2(3)
C6–N1–C10–C11	−73.6(3)
C6–C7–C8–C9	−50.9(3)
C7–C8–C9–C10	52.2(3)
C8–C9–C10–N1	−51.8(3)
C8–C9–C10–C11	72.0(3)
C9–C10–C11–O3	−15.1(4)
C9–C10–C11–N2	165.4(2)
C10–N1–C5–O1	−173.8(2)
C10–N1–C5–O2	6.1(4)
C10–N1–C6–C7	−51.4(3)
C11–N2–C12–C13	−67.8(3)
C11–N2–C12–C15	167.3(2)
C12–N2–C11–O3	−6.0(4)
C12–N2–C11–C10	173.5(2)
C13–C12–C15–C16	170.7(2)
C13–C12–C15–C17	−63.9(3)
C14–O5–C13–O4	1.2(4)
C14–O5–C13–C12	−178.4(2)
C15–C12–C13–O4	107.1(3)
C15–C12–C13–O5	−73.3(3)

6. NMR Spectra of Non-Commercial Starting Materials

^{1}H NMR (500 MHz, $CDCl_3$) of **SI-1** (Mixture of *cis*/*trans* isomers arising from the carbamate.):

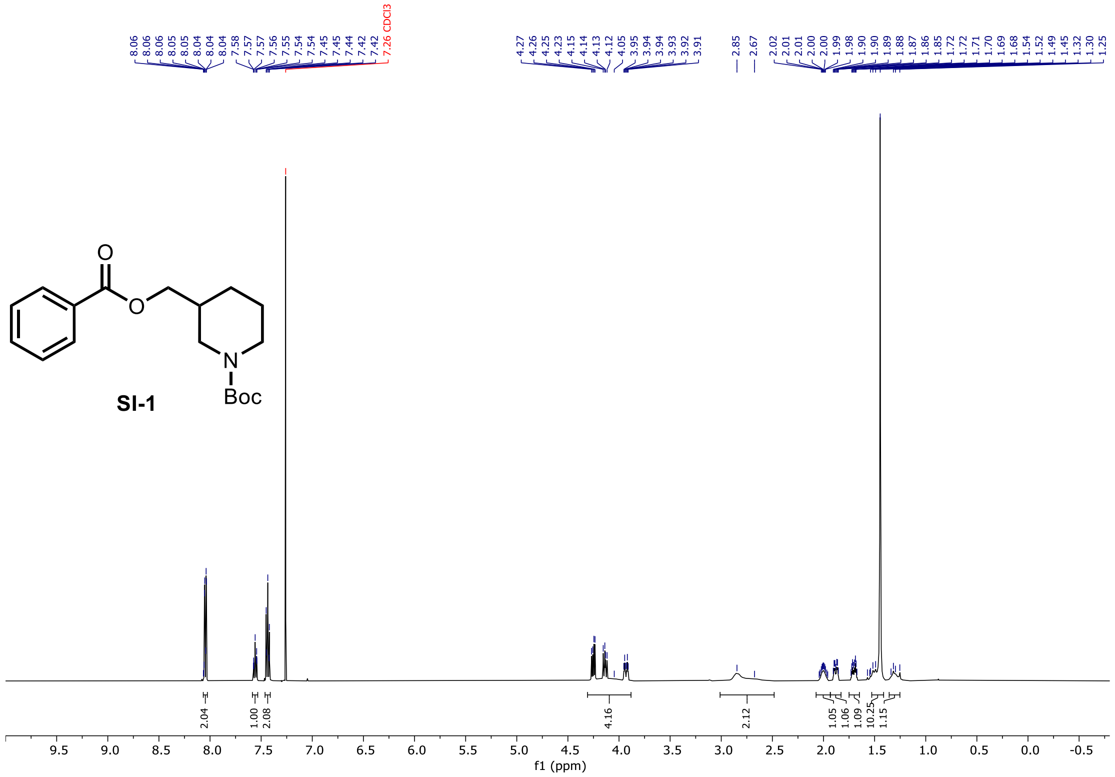

^{13}C NMR (126 MHz, $CDCl_3$) of **SI-1**:

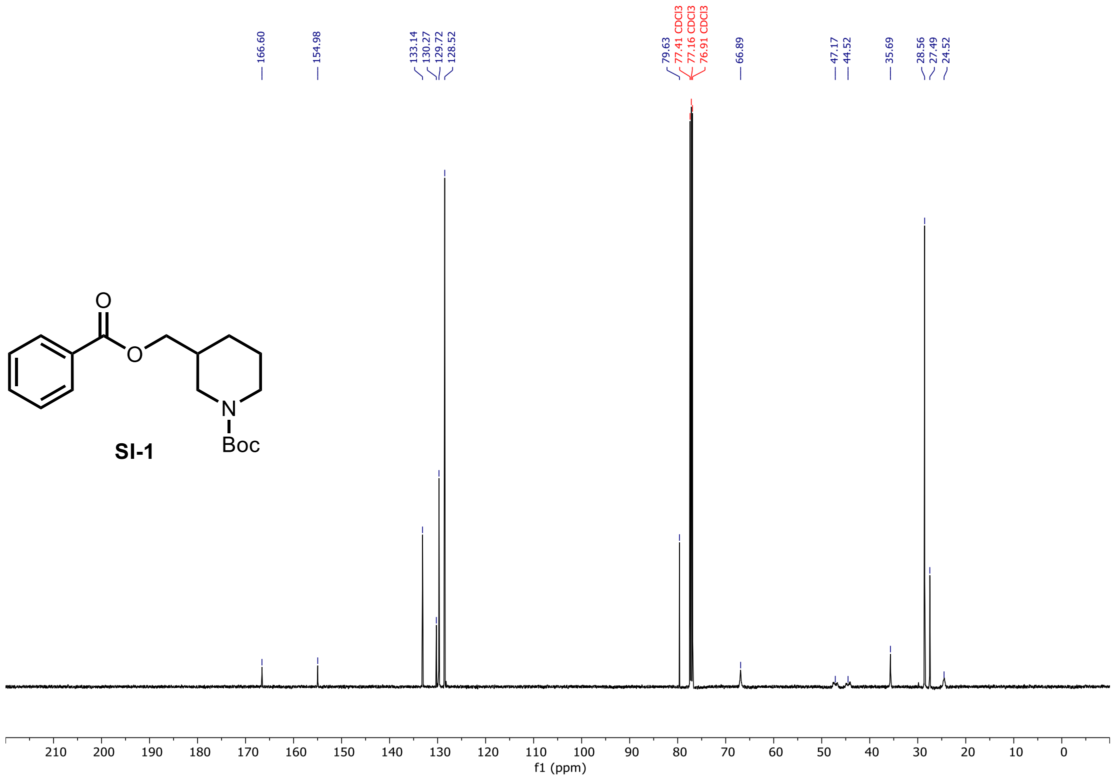

^{1}**H NMR** (500 MHz, $CDCl_3$) of **39** (Mixture of *cis/trans* isomers arising from the carbamate.):

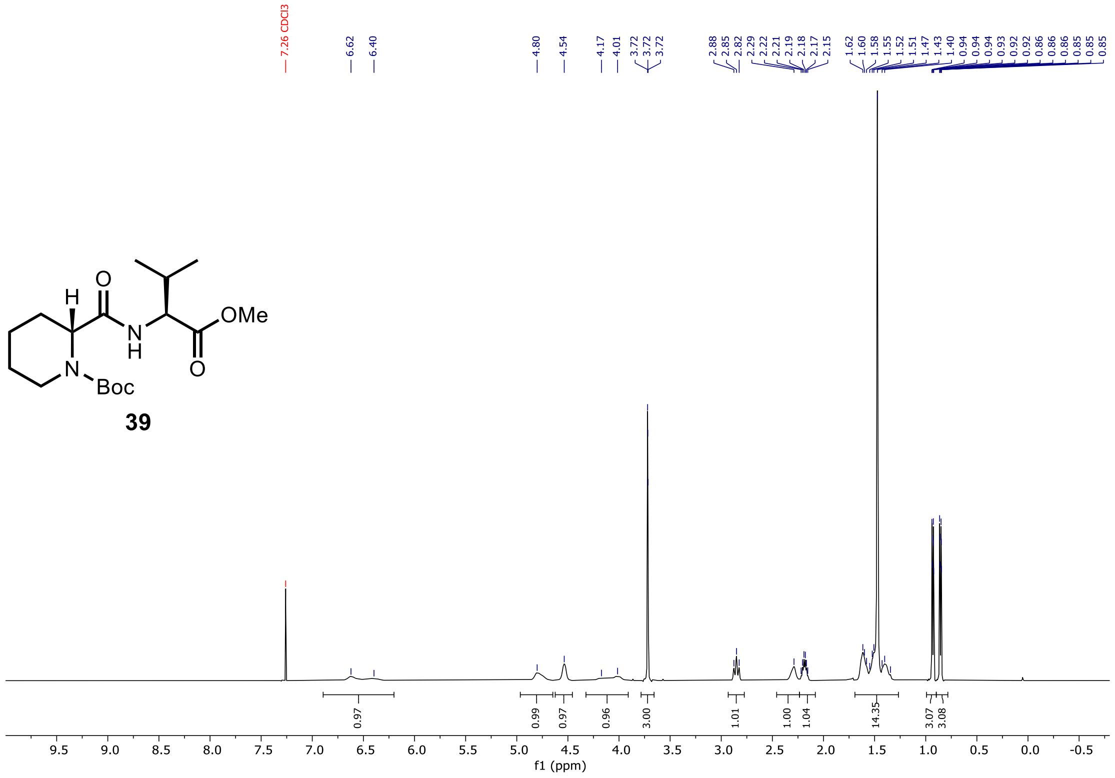

^{13}**C NMR** (126 MHz, $CDCl_3$) of **39**:

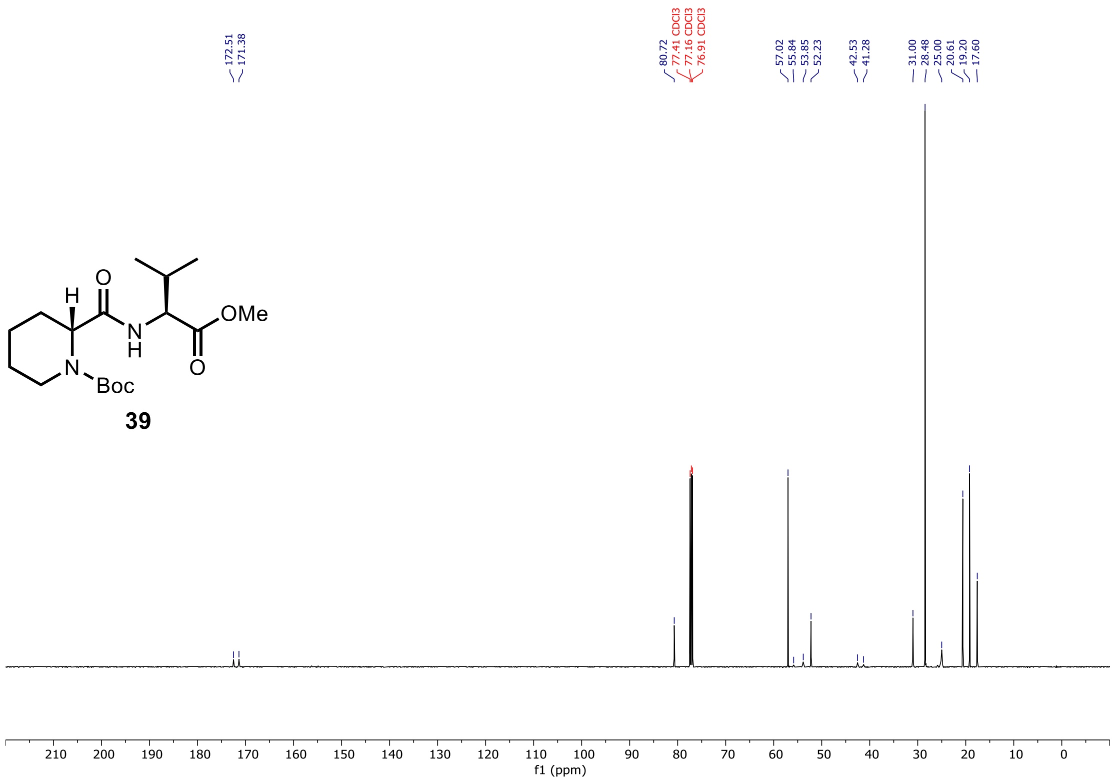

7. NMR Spectra of Products

7.1 Hemiaminals

^{1}H NMR (500 MHz, $CDCl_3$) of **3** (relative configuration, *trans*):

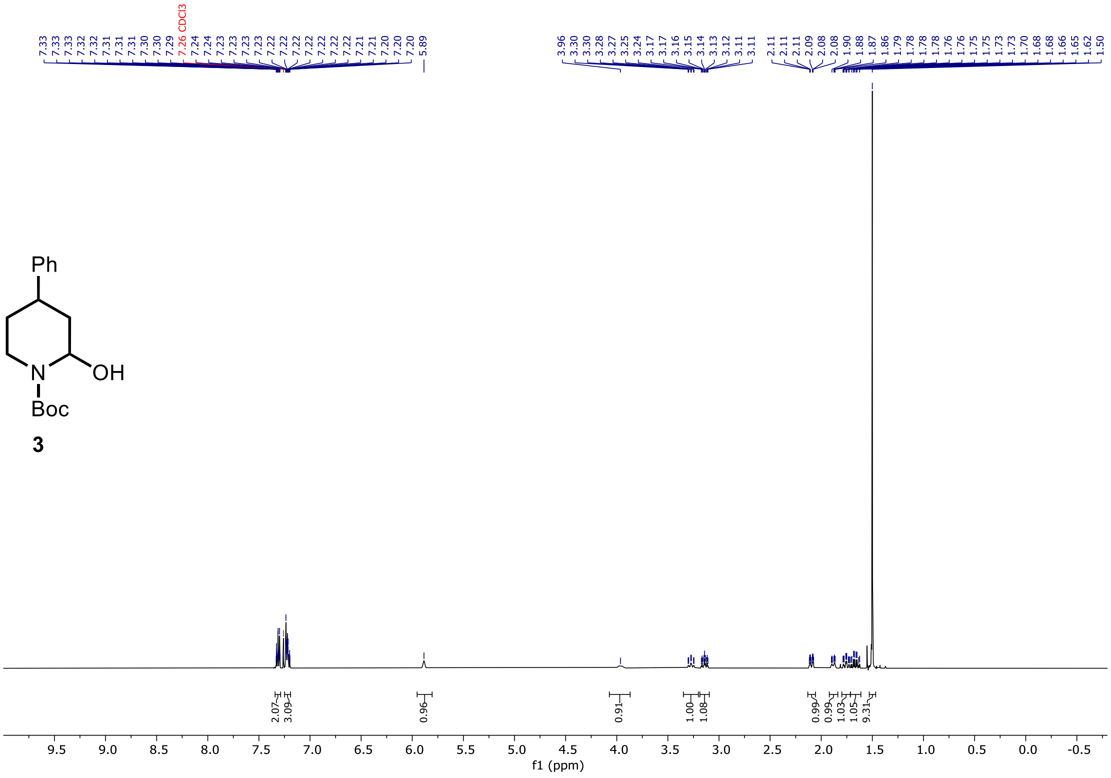

^{13}C NMR (126 MHz, $CDCl_3$) of **3**:

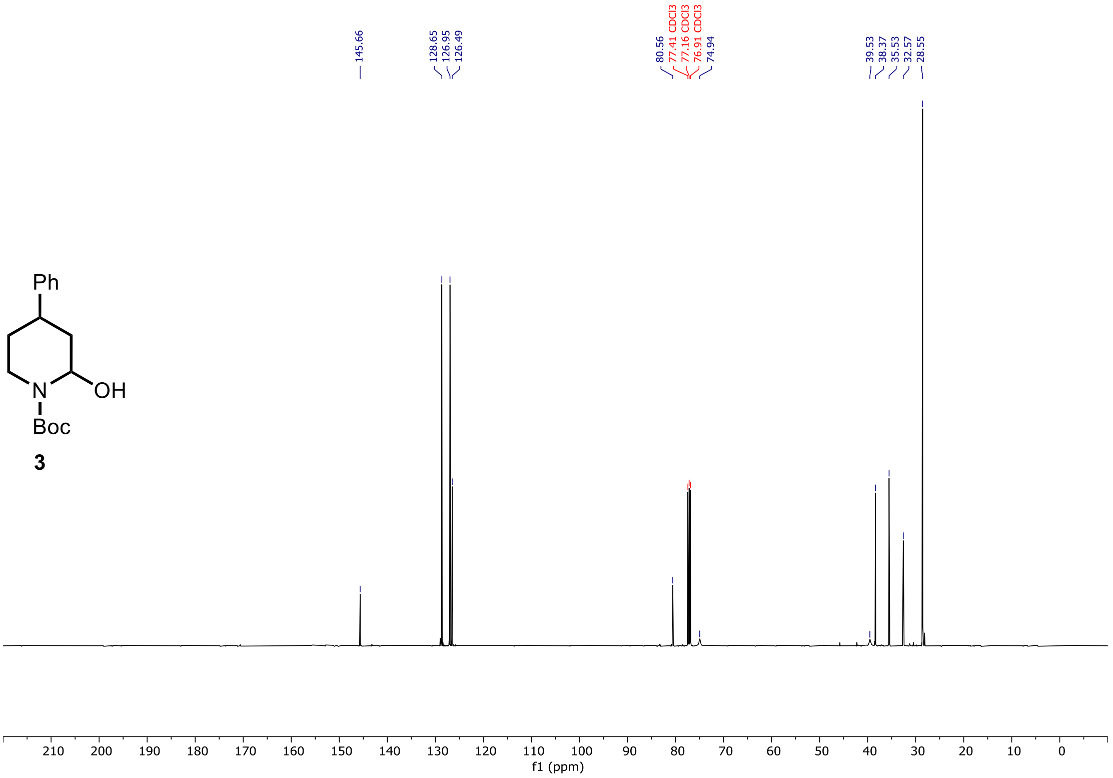

[1]**H NMR** (500 MHz, $CDCl_3$) of **4** (relative configuration, *trans*):

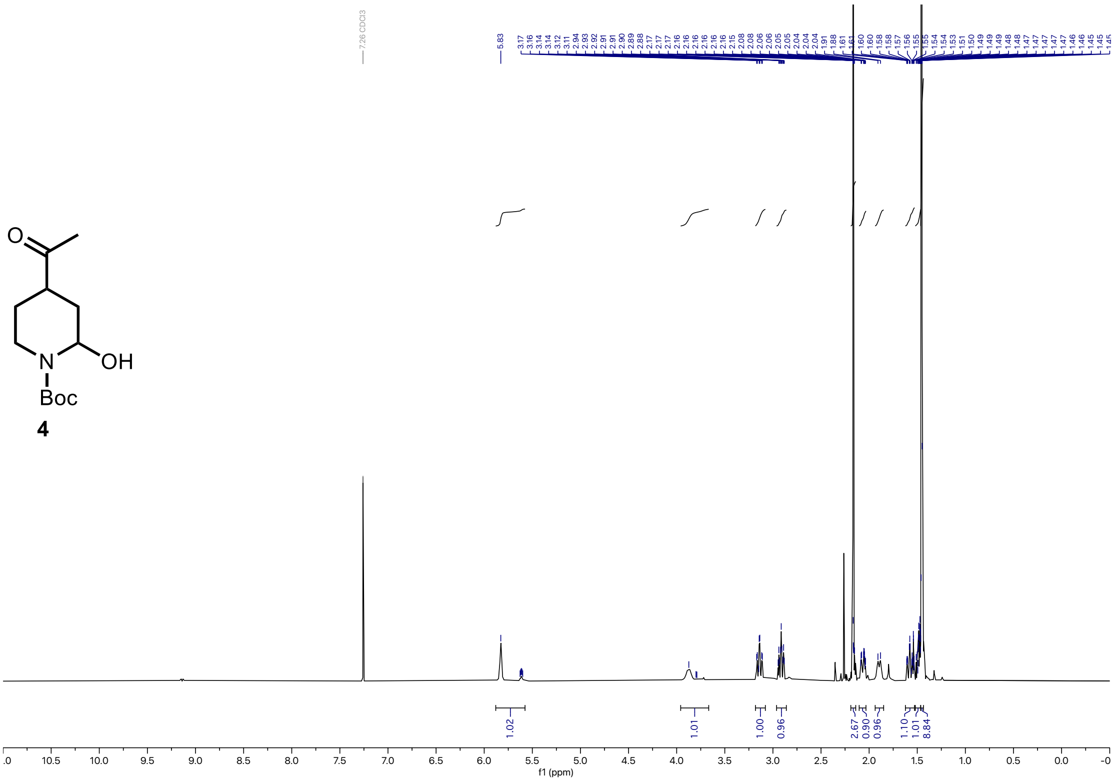

[13]**C NMR** (126 MHz, $CDCl_3$) of **4**:

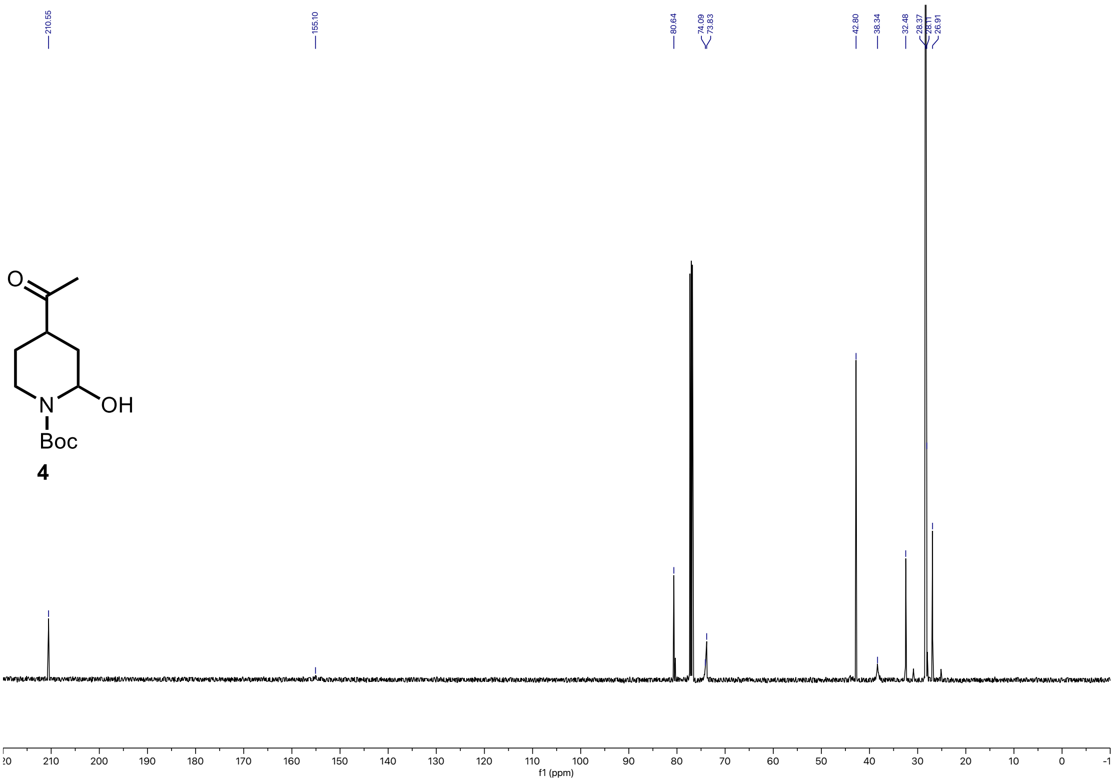

^{1}H NMR (500 MHz, $CDCl_3$) of **5** (relative configuration, *trans*):

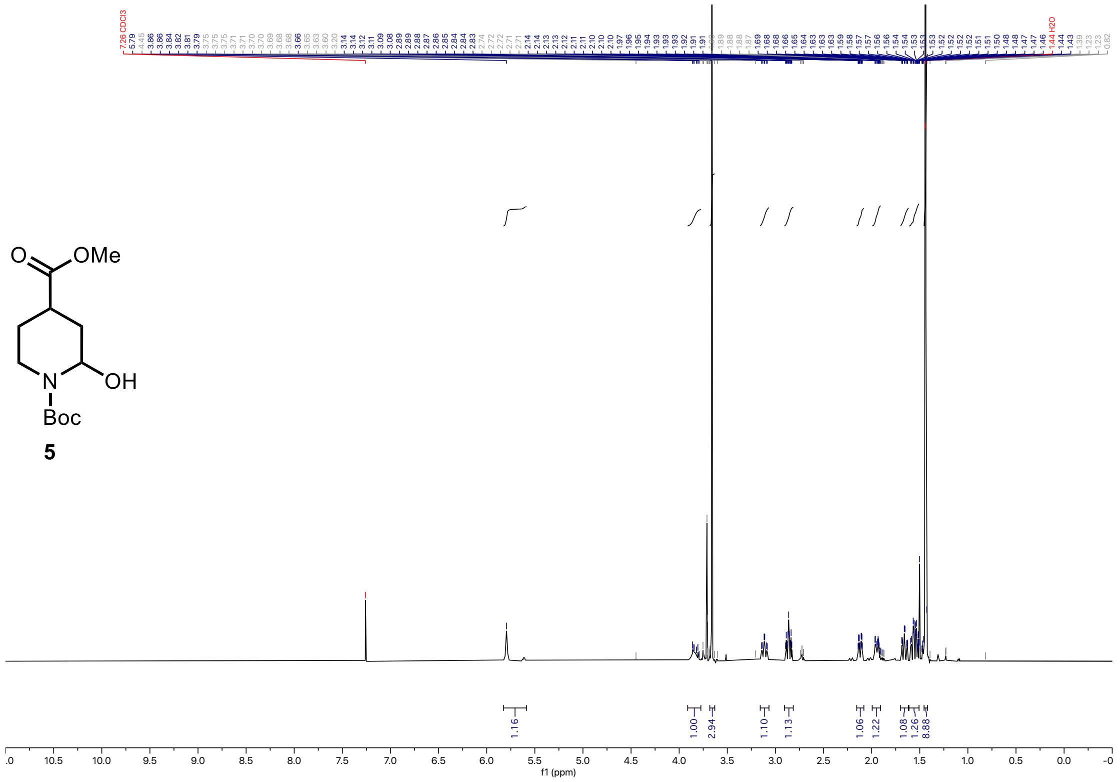

^{13}C NMR (126 MHz, $CDCl_3$) of **5**:

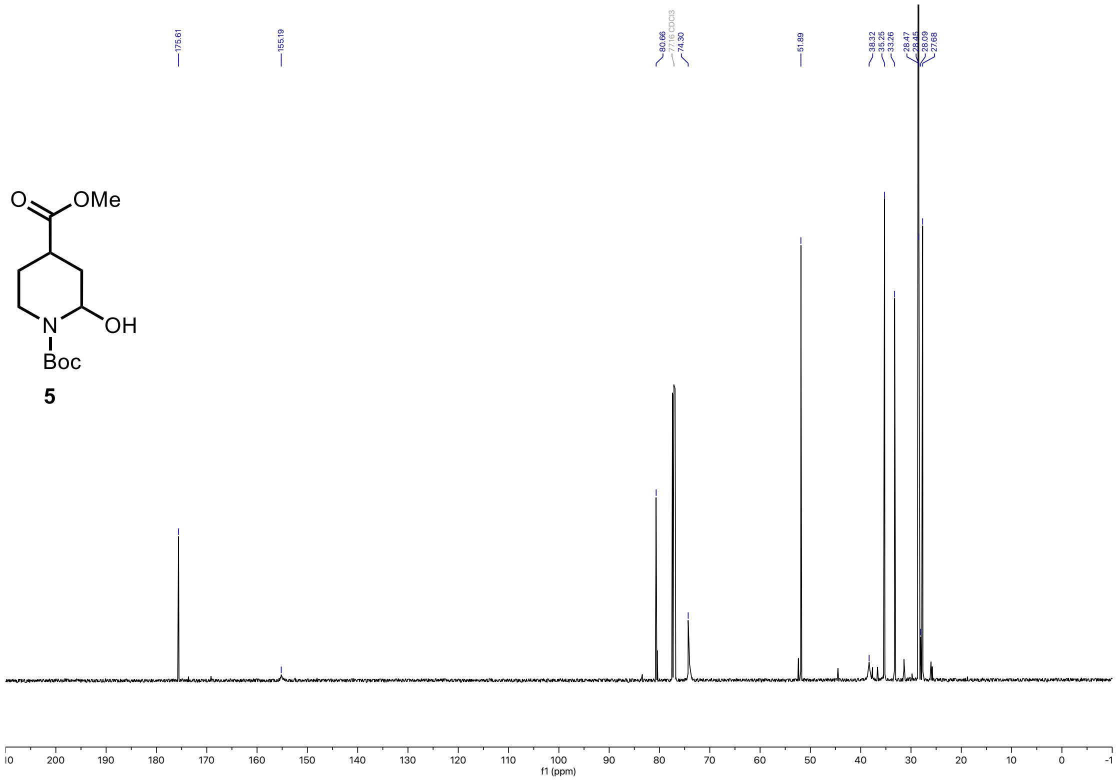

^{1}**H NMR** (500 MHz, $CDCl_3$) of **6** (relative configuration, *trans*):

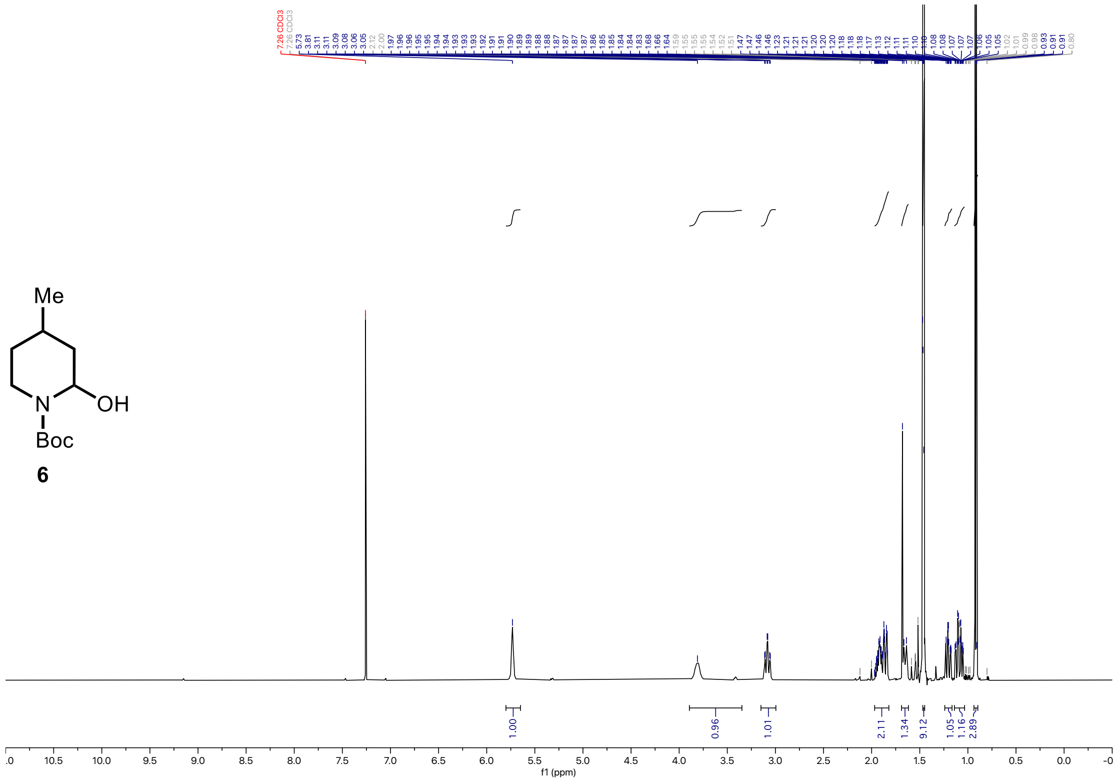

^{13}**C NMR** (126 MHz, $CDCl_3$) of **6**:

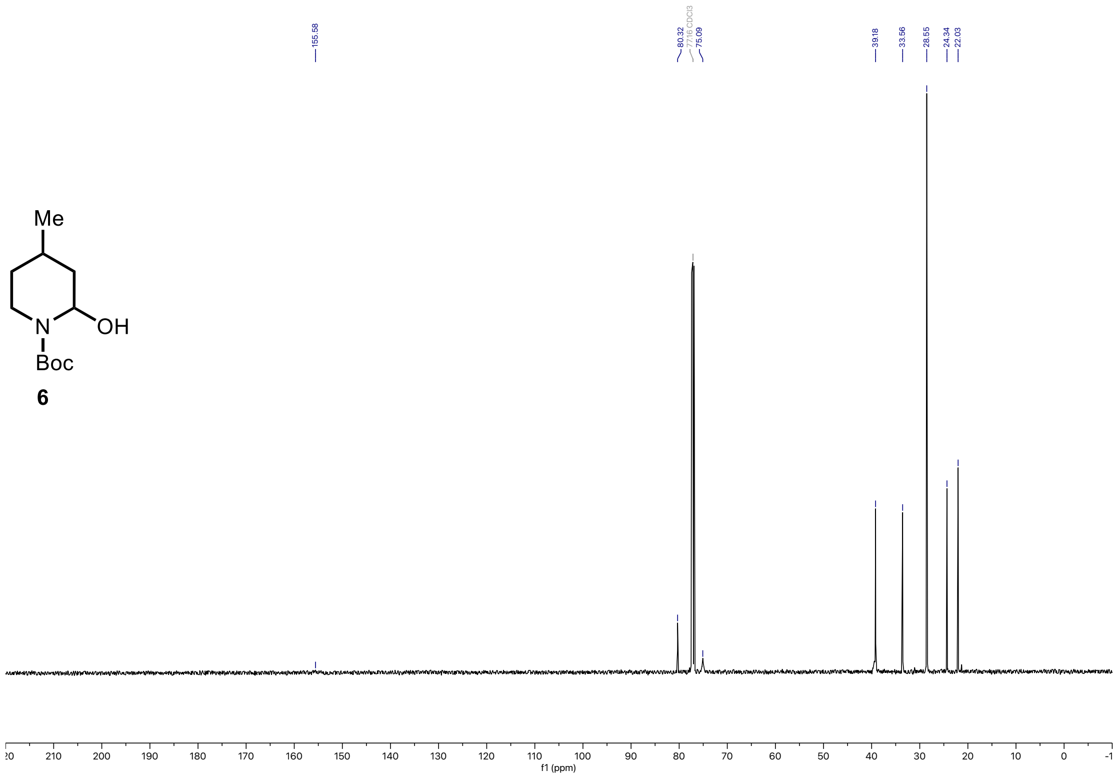

^{1}H NMR (400 MHz, $CDCl_3$) of **7**:

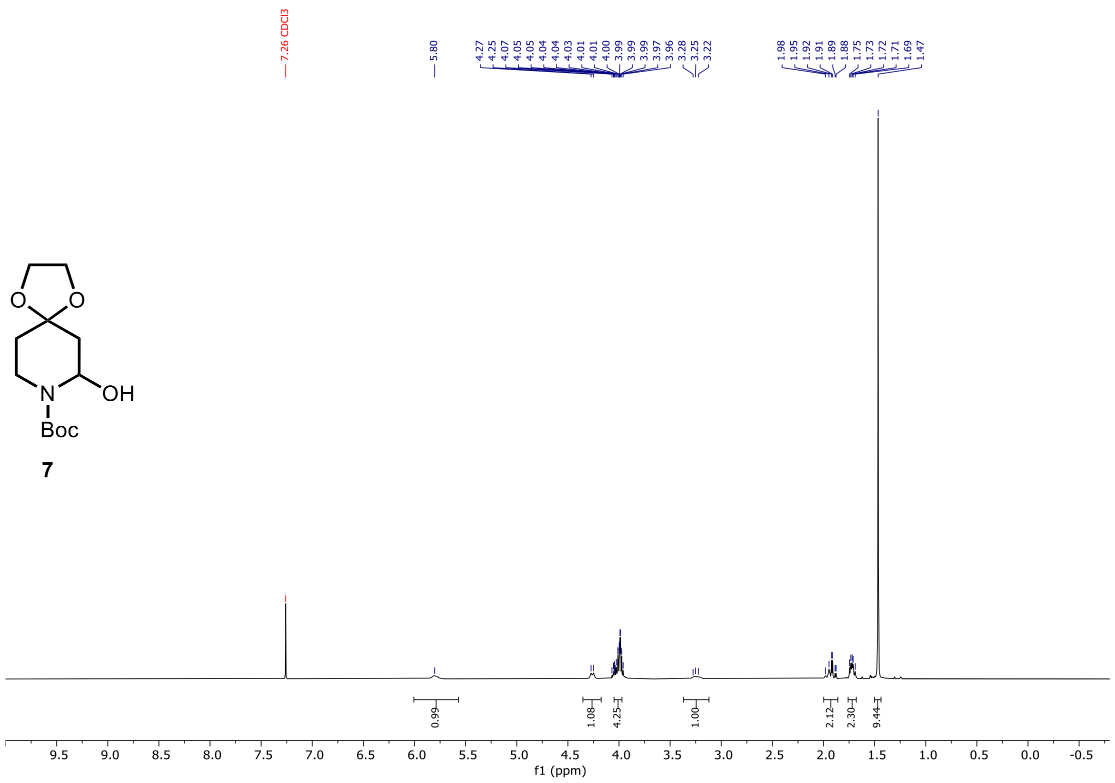

^{13}C NMR (101 MHz, $CDCl_3$) of **7**:

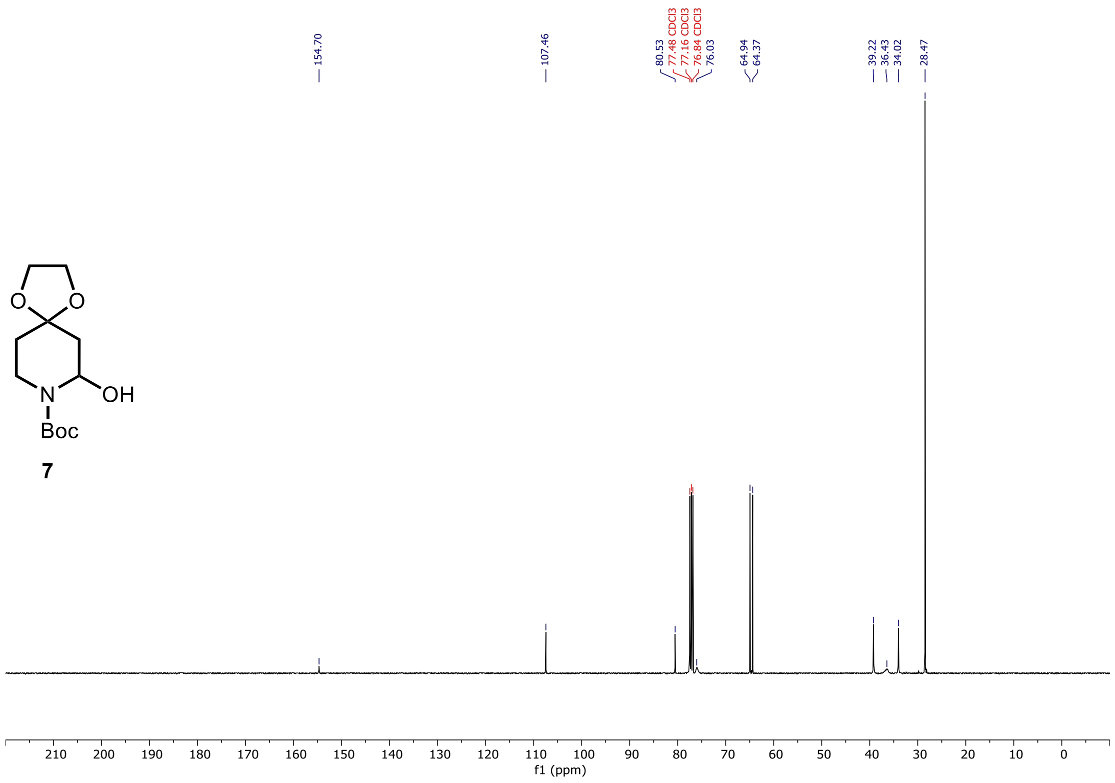

^{1}**H NMR** (500 MHz, $CDCl_3$) of **9a** and **9b**:

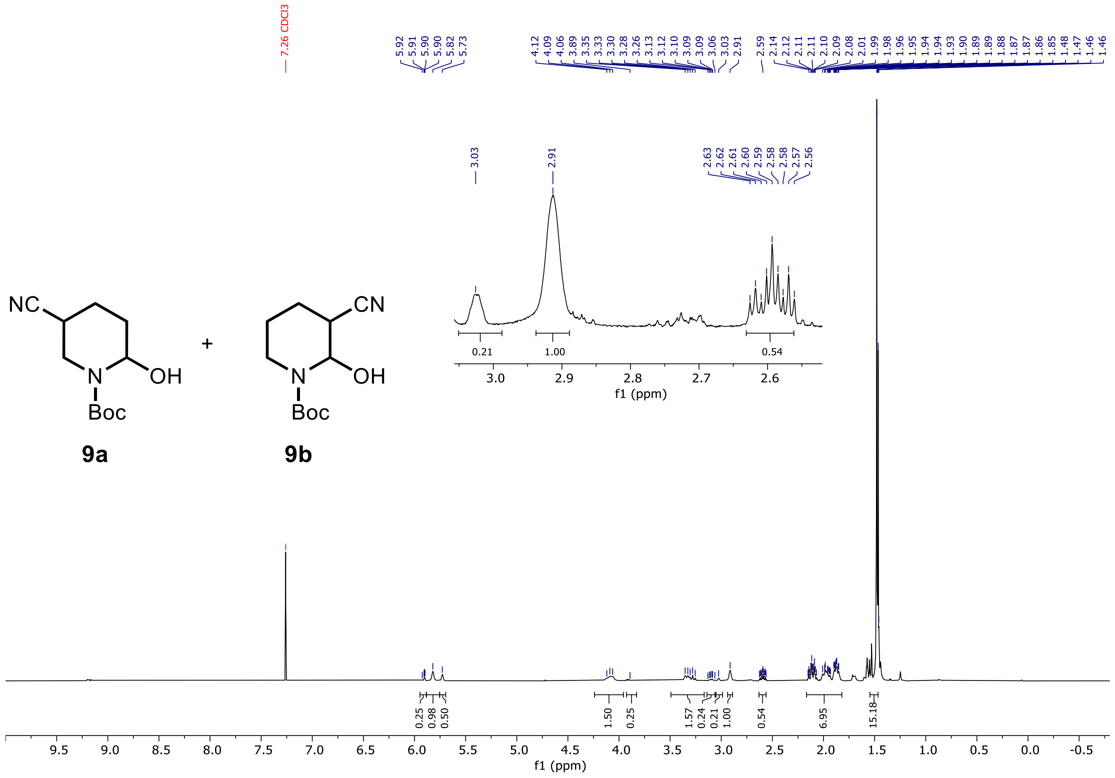

^{13}**C NMR** (126 MHz, $CDCl_3$) of **9a** and **9b**:

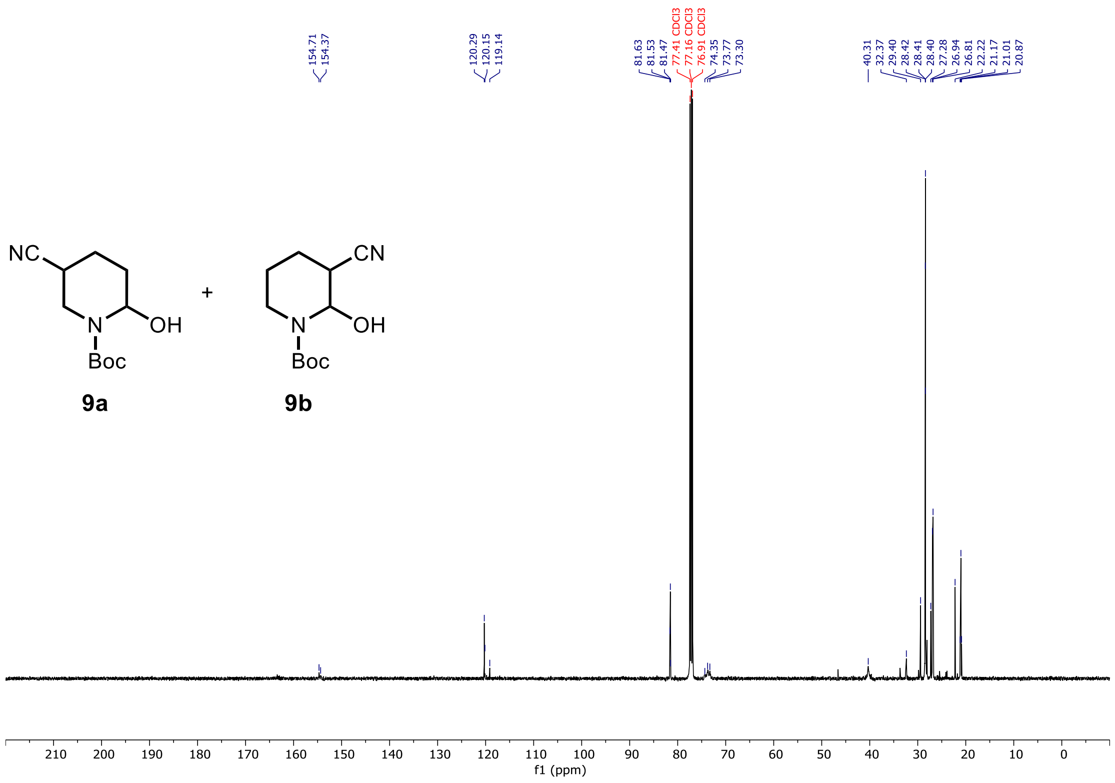

^{1}H-^{13}H **COSY** (CDCl$_3$) of **9a** and **9b**:

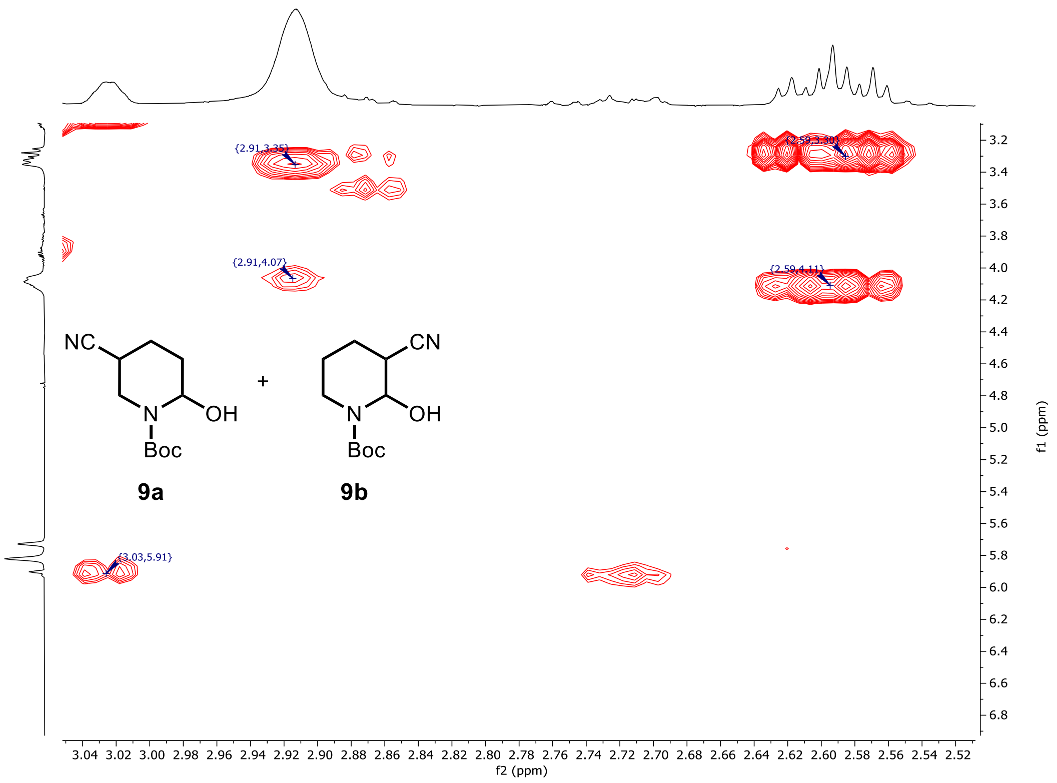

^{1}H-^{13}C **HSQC** (CDCl$_3$) of **9a** and **9b**:

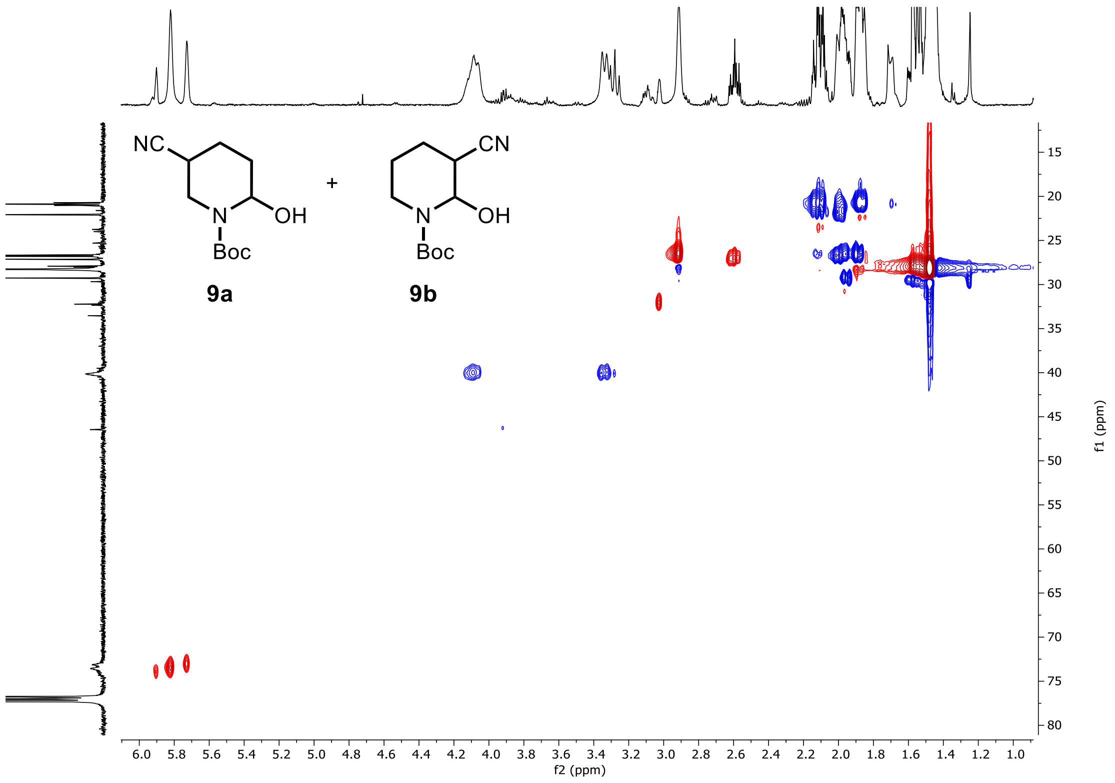

^{1}H NMR (500 MHz, $CDCl_3$) of **10a** and **10b**:

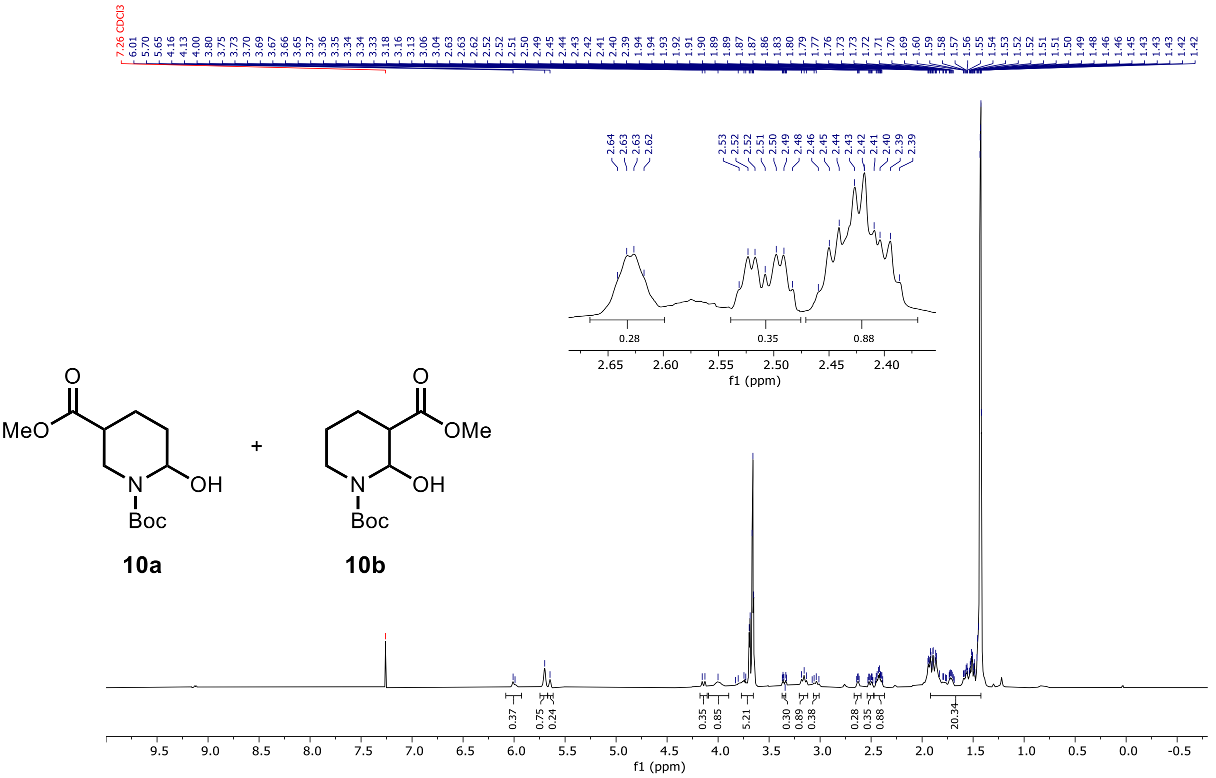

^{13}C NMR (126 MHz, $CDCl_3$) of **10a** and **10b:**

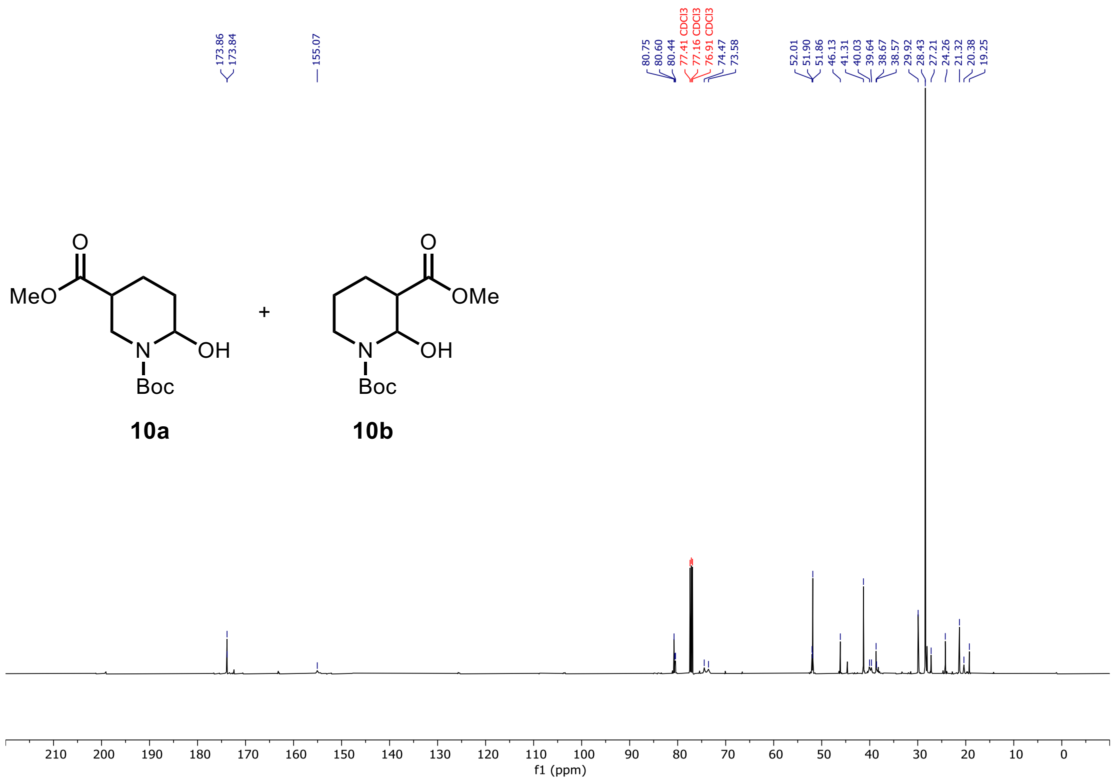

^{1}H-^{13}H COSY (CDCl$_3$) of **10a** and **10b**:

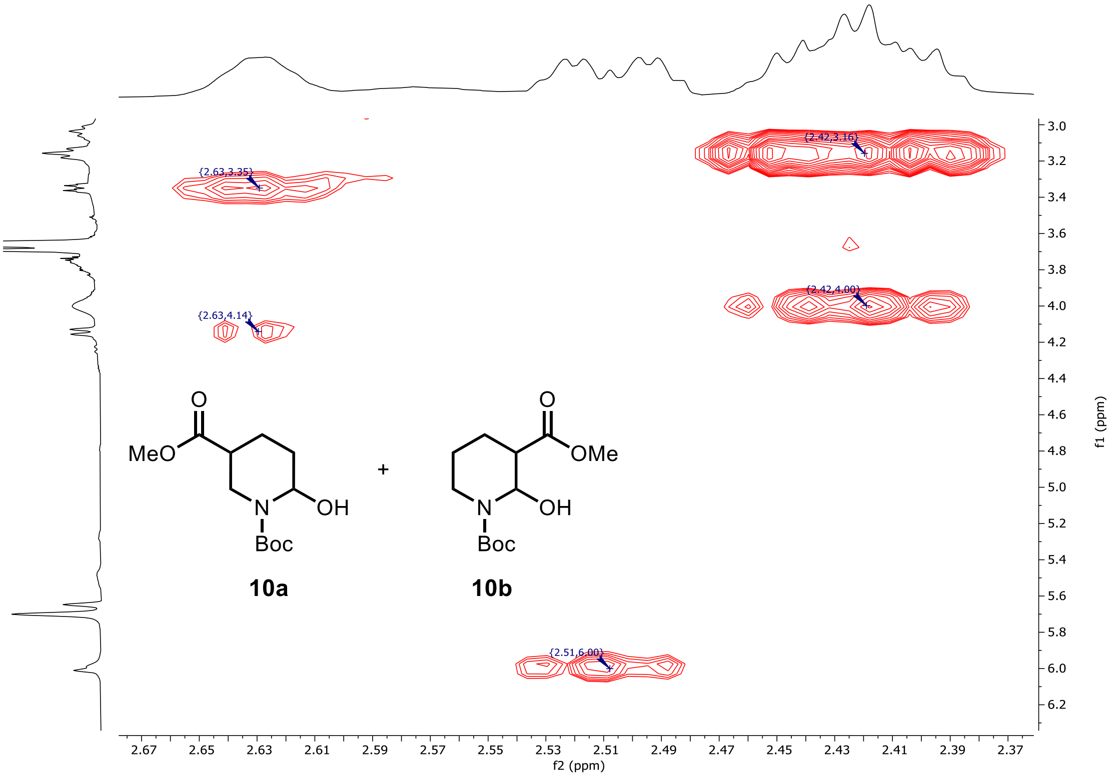

^{1}H-^{13}C HSQC (CDCl$_3$) of **10a** and **10b**:

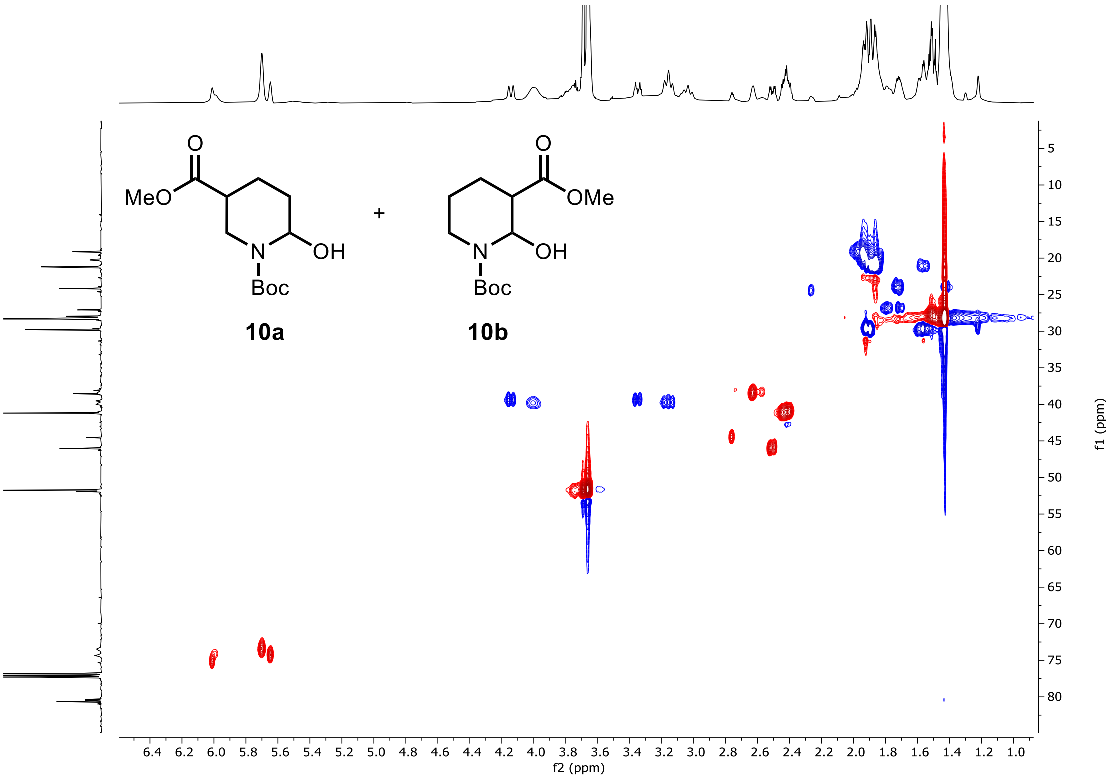

^{1}H NMR (500 MHz, $CDCl_3$) of **11** (relative configuration, *trans*):

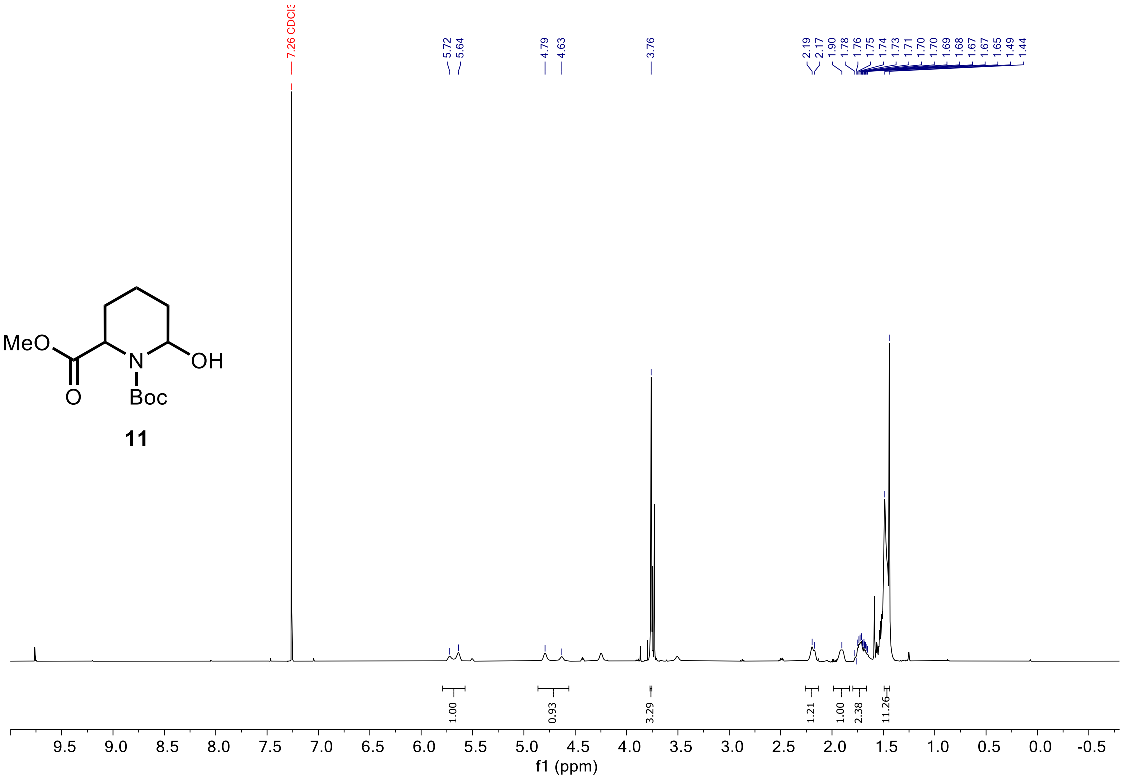

^{13}C NMR (126 MHz, $CDCl_3$) of **11**:

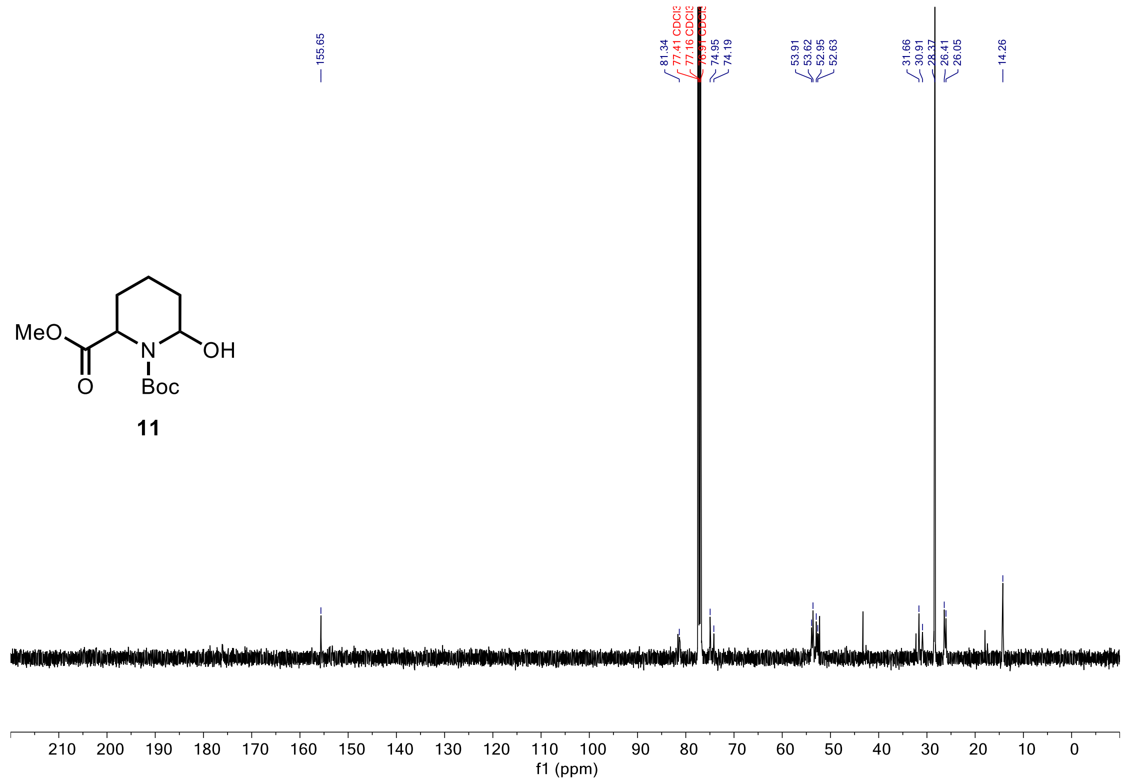

^{1}H NMR (500 MHz, $CDCl_3$) of **12** (relative configuration, *trans*):

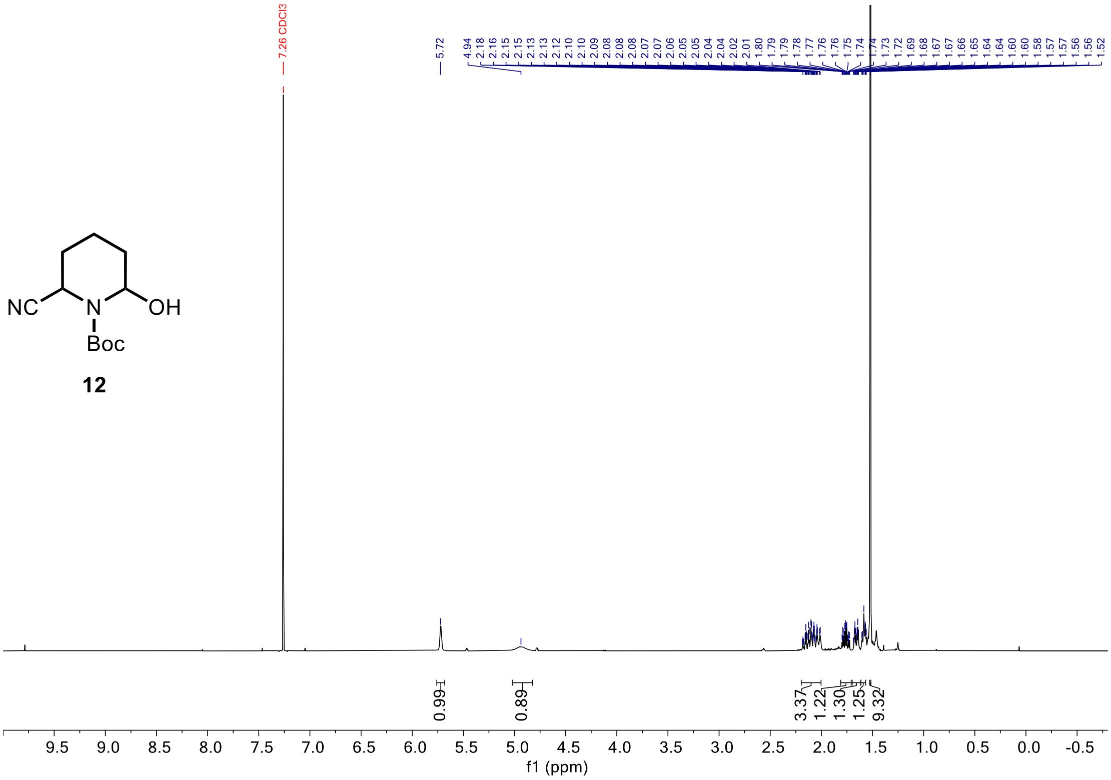

^{13}C NMR (126 MHz, $CDCl_3$) of **12**:

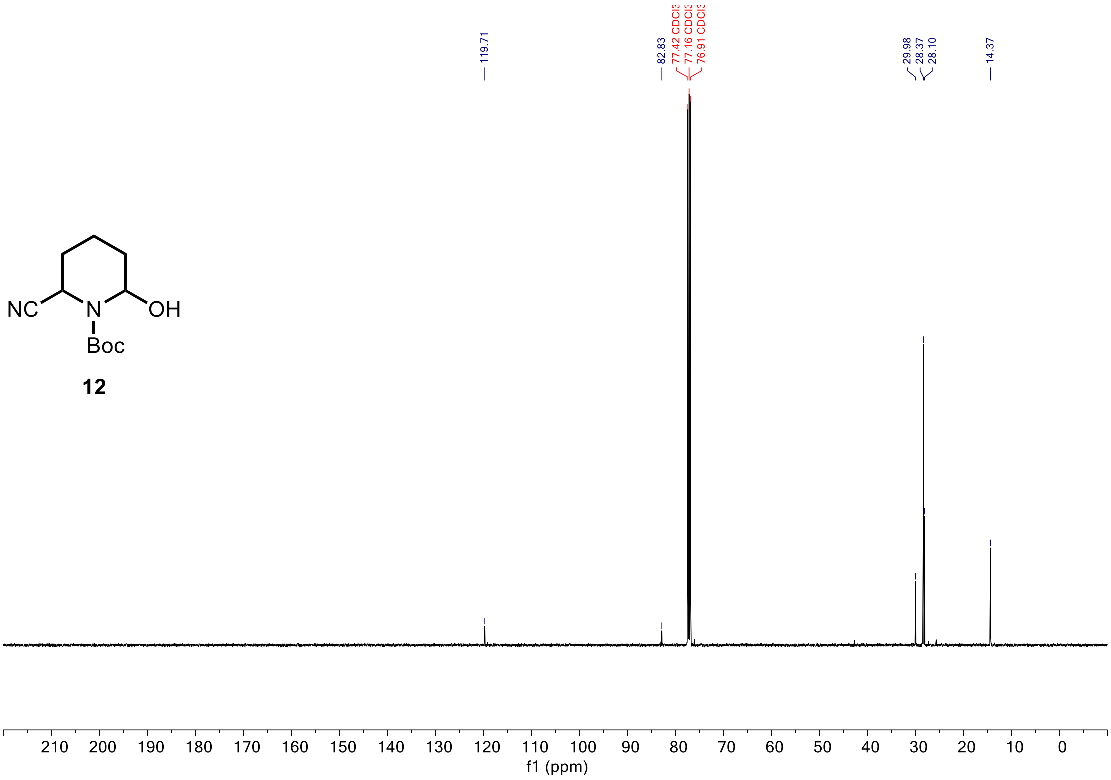

^{1}H-^{13}C HSQC (CDCl$_3$) of **12**:

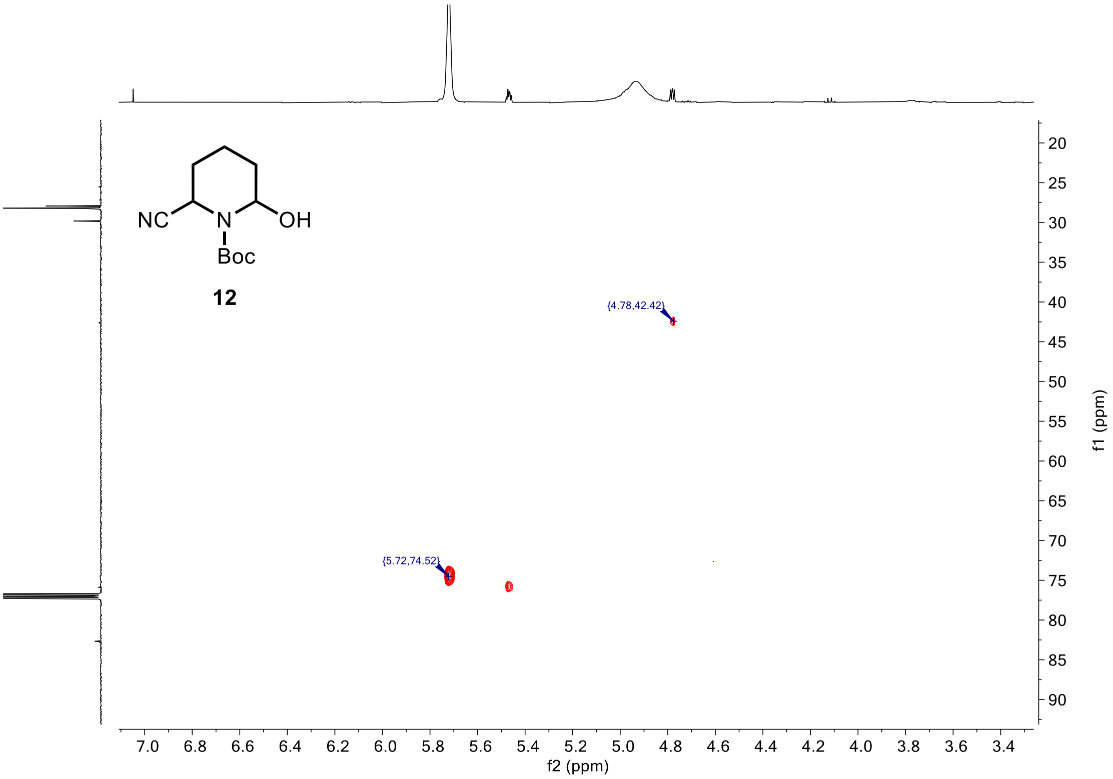

^{1}H NMR (500 MHz, CDCl$_3$) of **14** (Mixture of *cis/trans* isomers arising from the carbamate.):

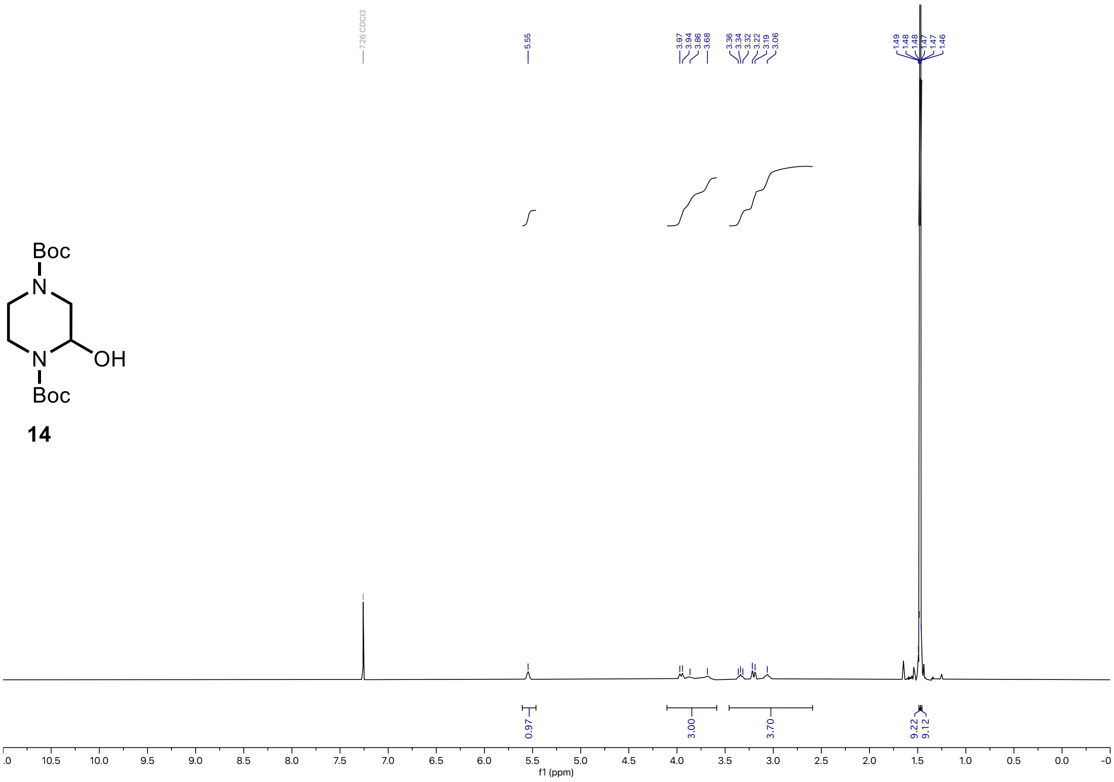

^{13}C NMR (126 MHz, $CDCl_3$) of **14**:

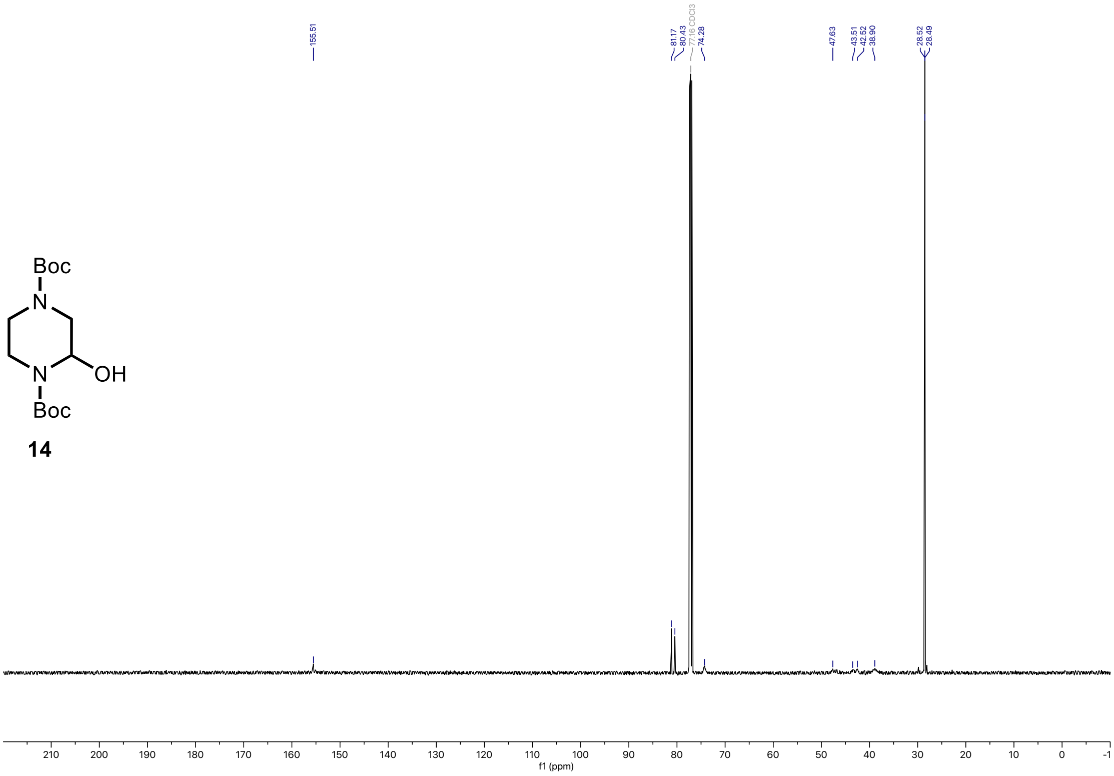

14

^{1}H NMR (500 MHz, $CDCl_3$) of **15**:

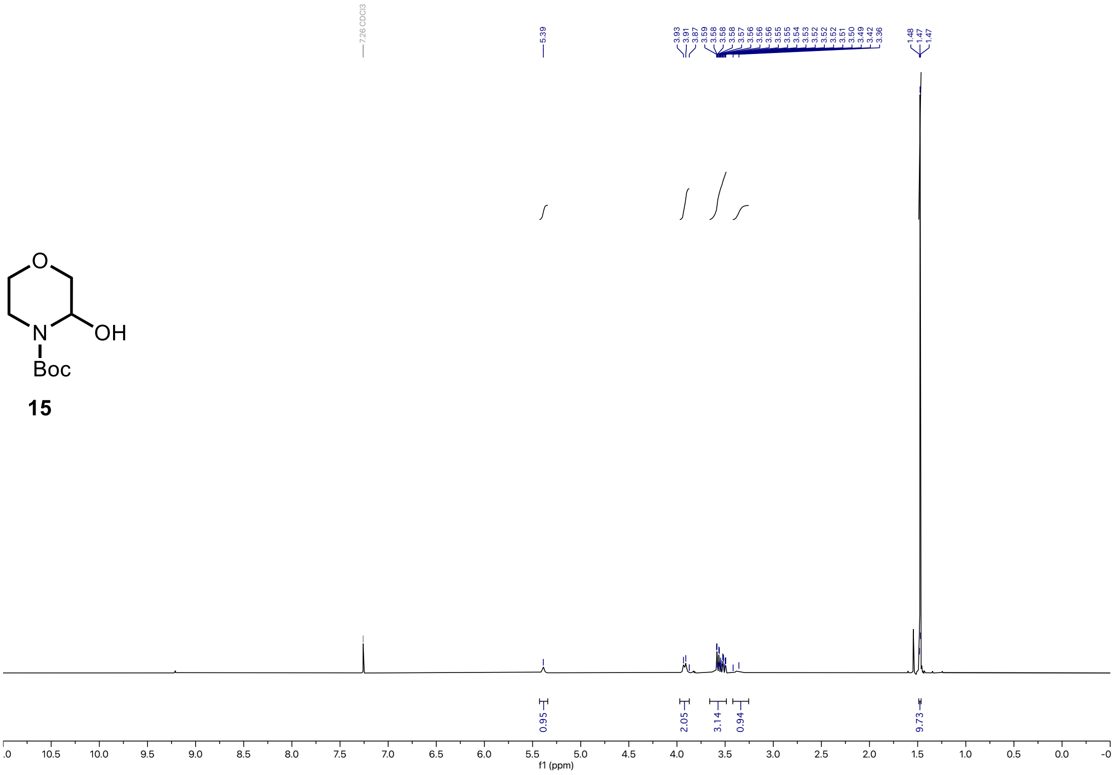

15

^{13}C NMR (126 MHz, $CDCl_3$) of **15**:

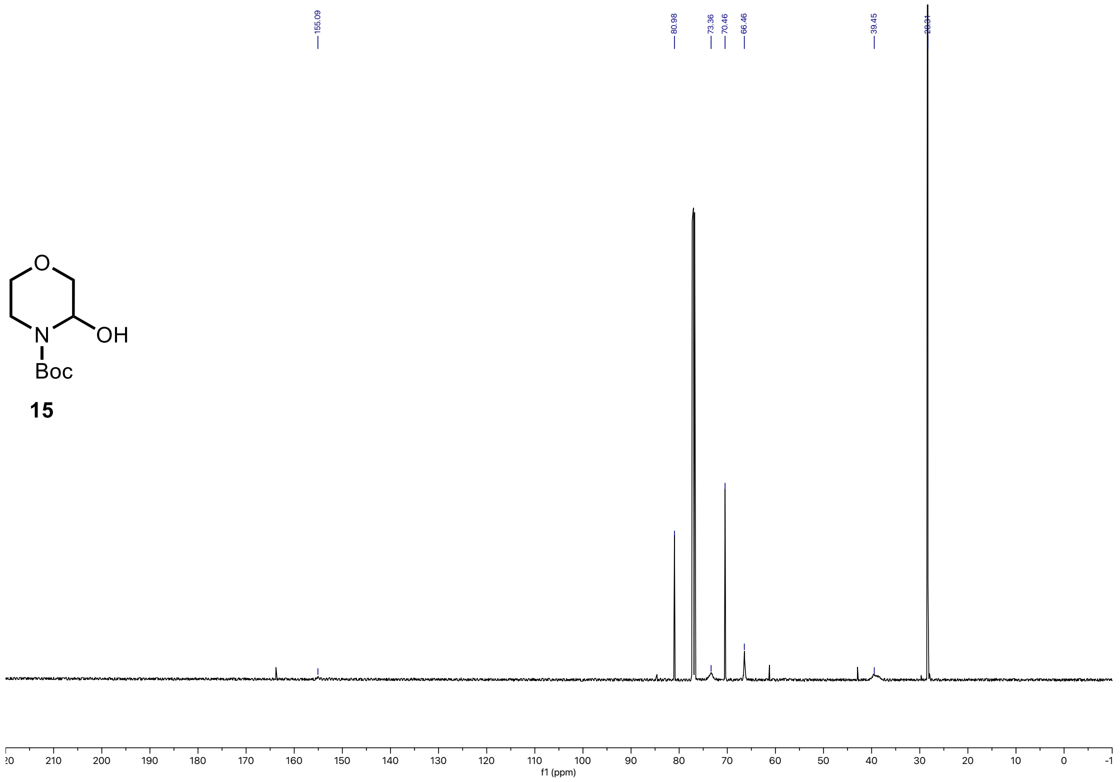

^{1}H NMR (500 MHz, $CDCl_3$) of **18**:

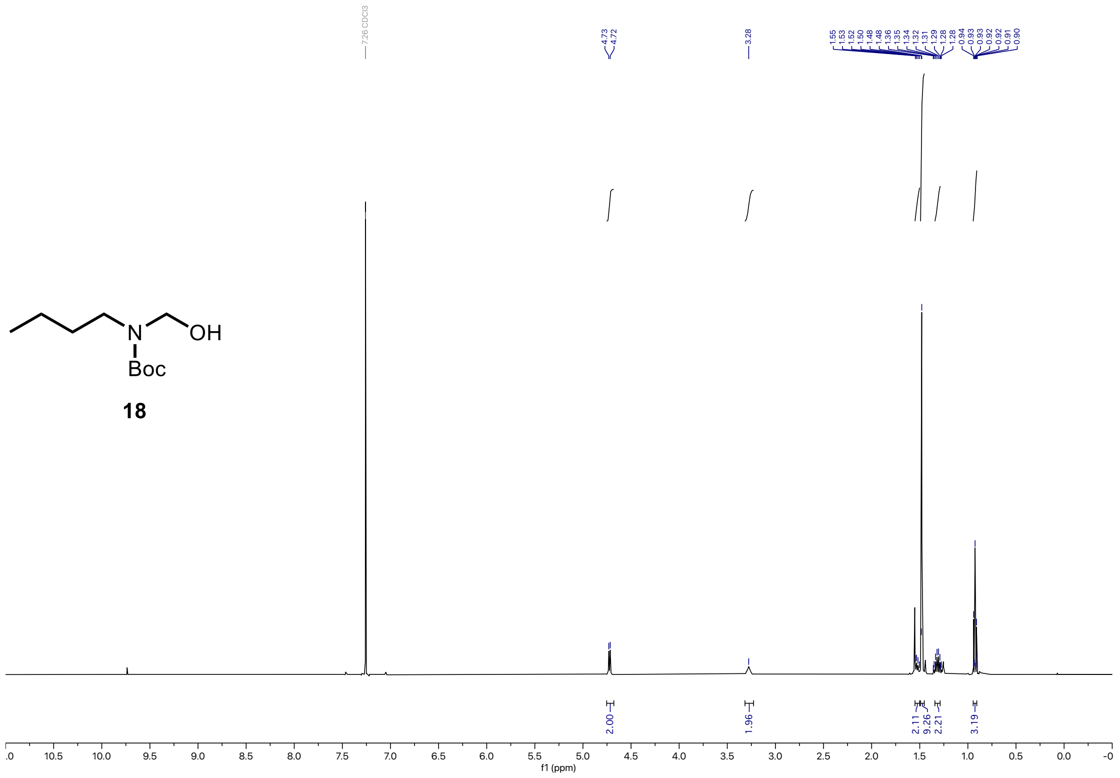

^{13}C NMR (126 MHz, $CDCl_3$) of **18**:

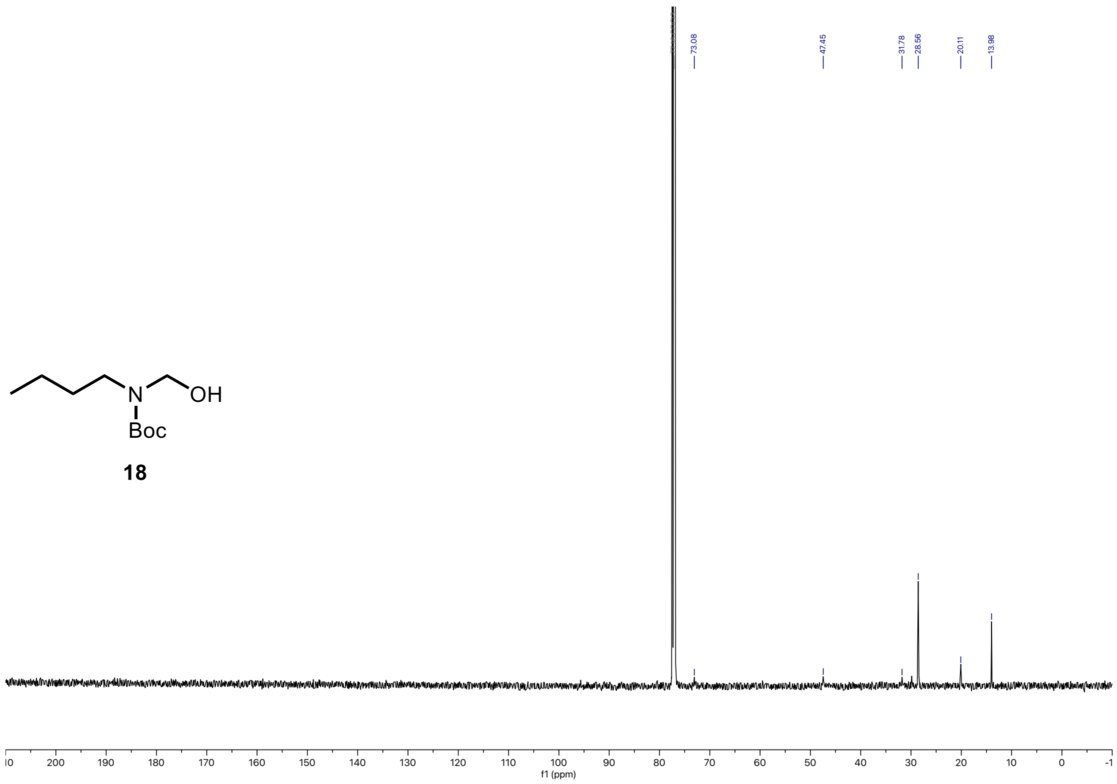

^{1}H-^{13}C HSQC ($CDCl_3$) of **18**:

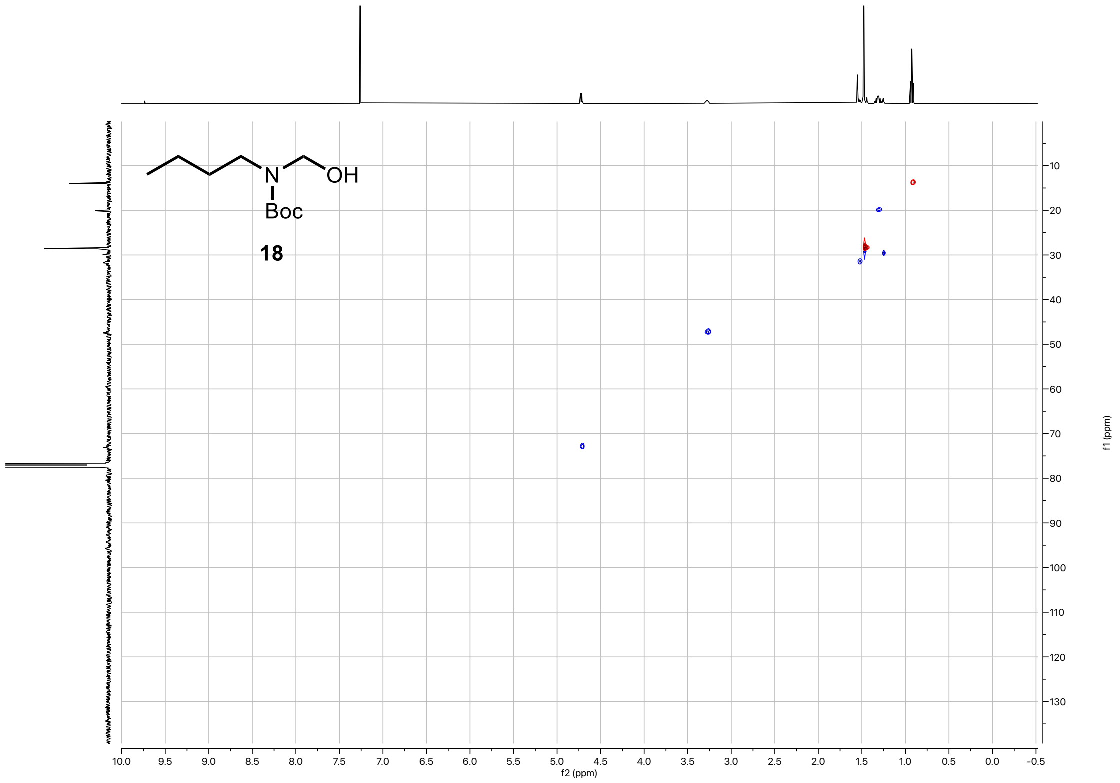

^{1}H-^{13}C HMBC (CDCl$_3$) of **18**:

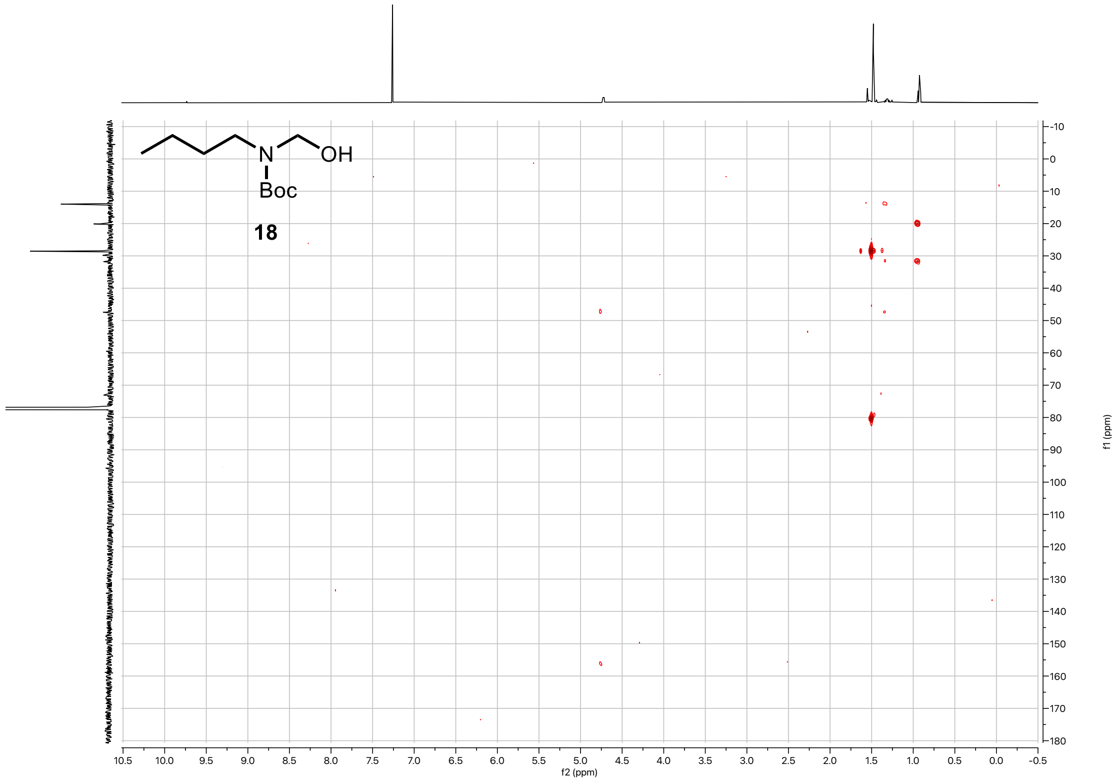

^{1}H NMR (500 MHz, CDCl$_3$) of **40** (relative configuration at piperidine *trans*):

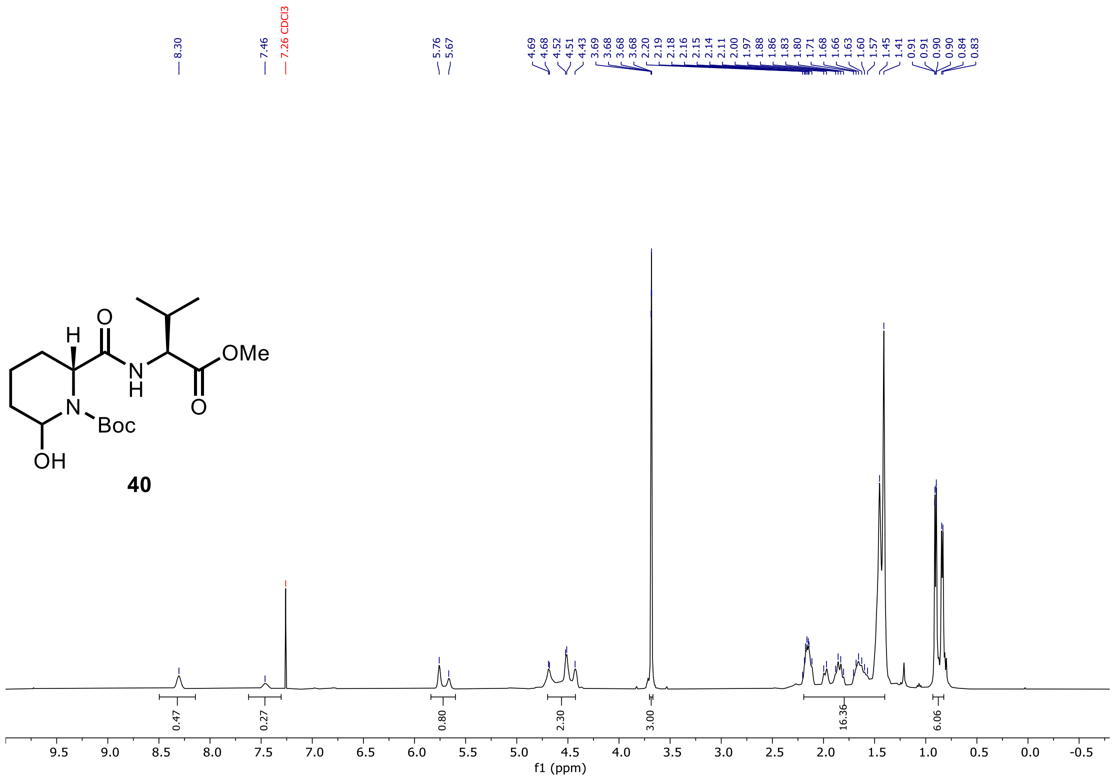

^{13}**C NMR** (126 MHz, $CDCl_3$) of **40**:

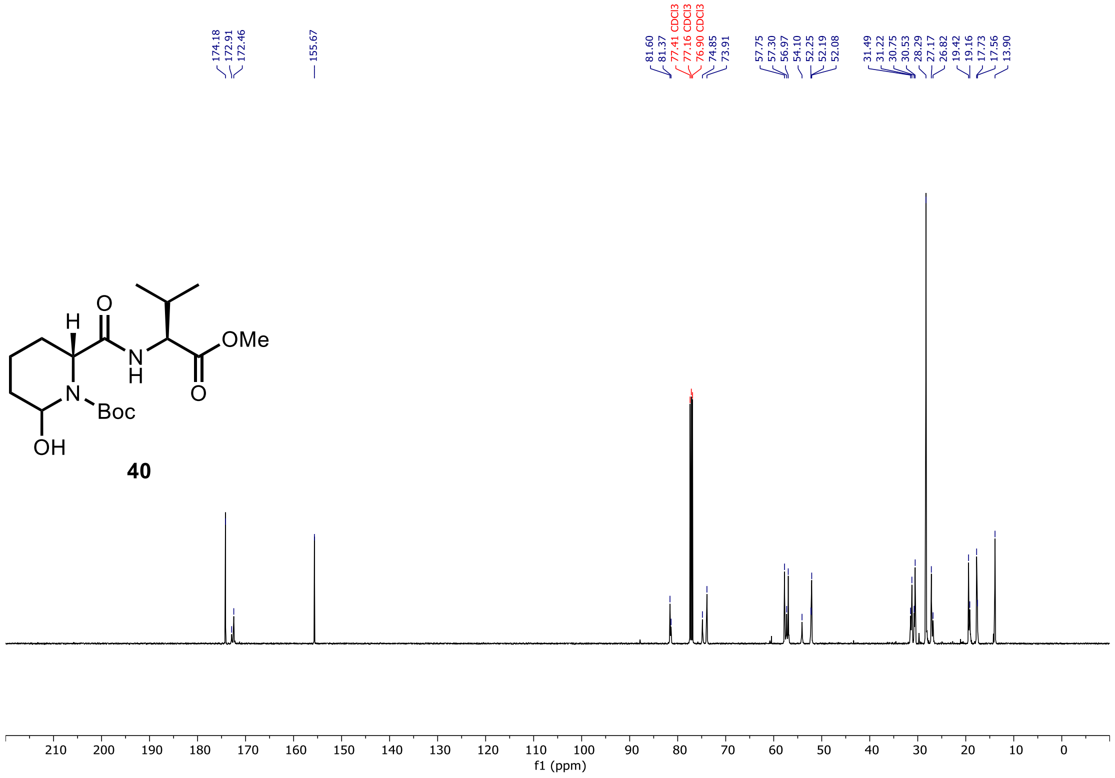

^{1}**H NMR** (500 MHz, DMSO) of **40**:

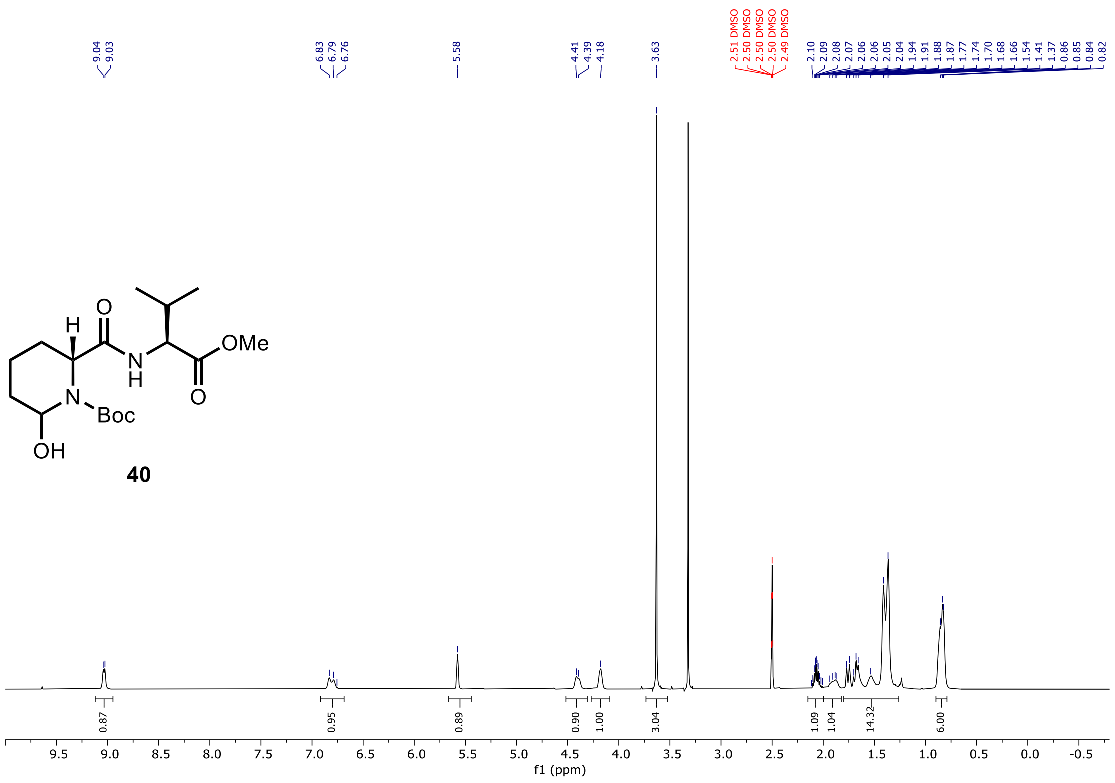

NOESY (500 MHz, $CDCl_3$) of **40**:

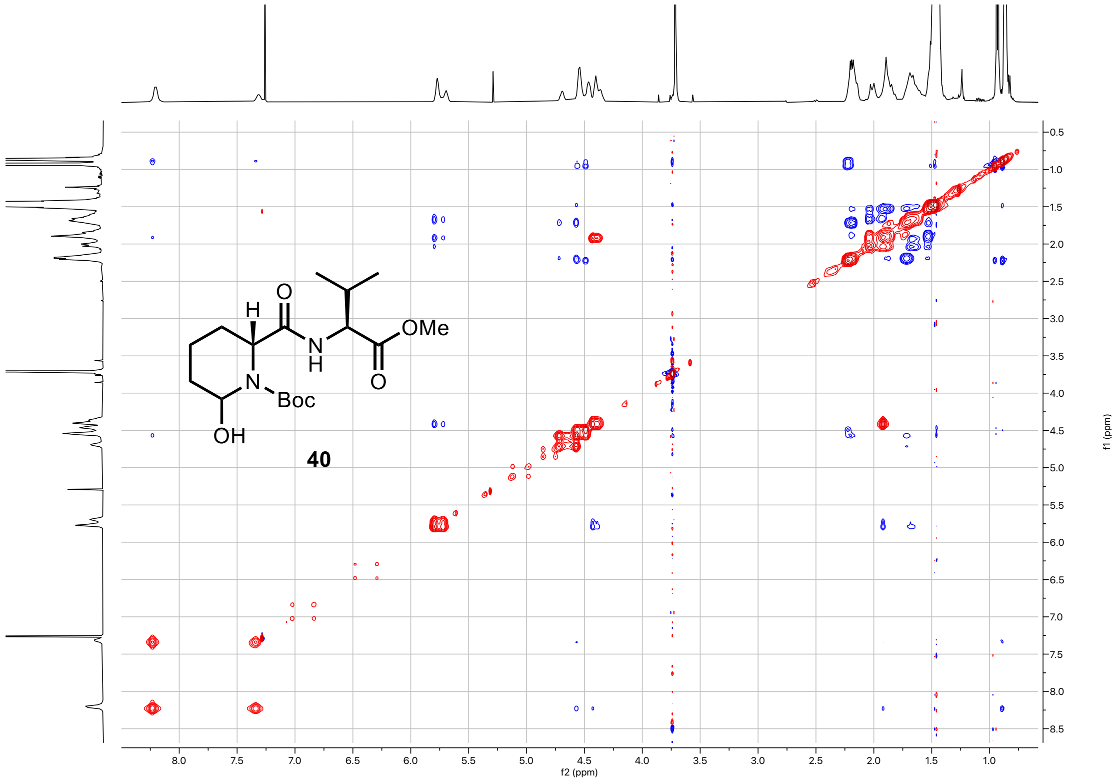

COSY (500 MHz, $CDCl_3$) of **40:**

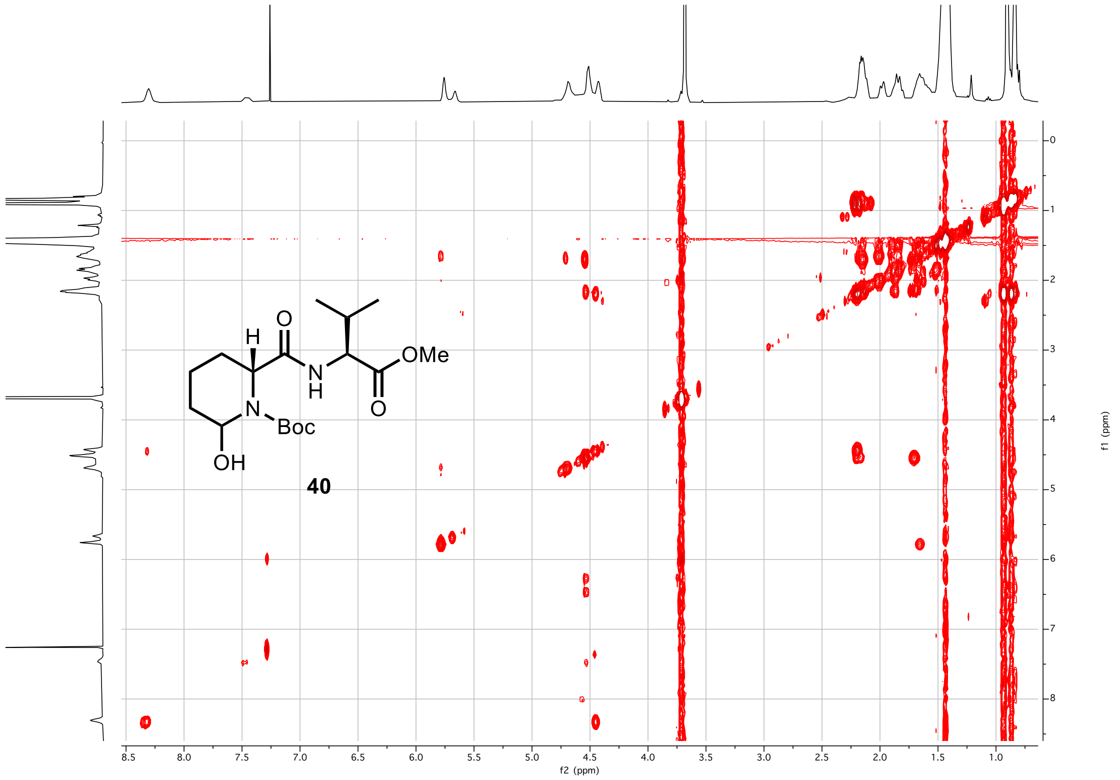

7.2 Enecarbamates

^{1}H NMR (500 MHz, $CDCl_3$) of **19**:

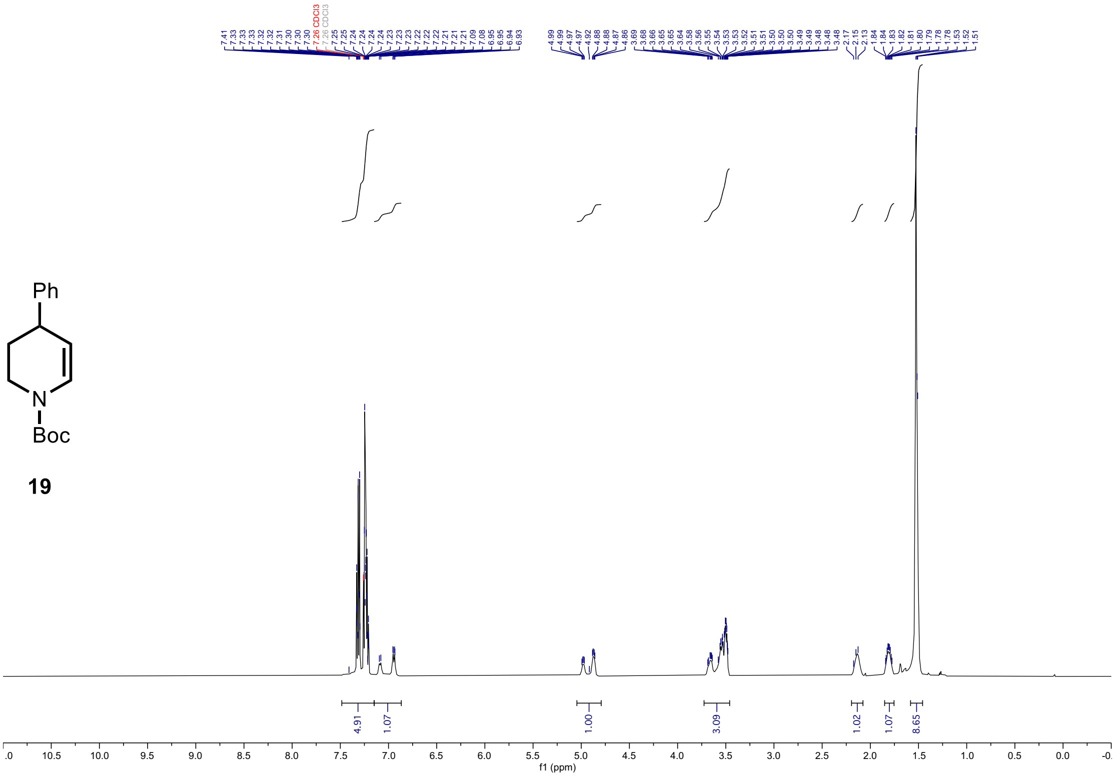

19

^{13}C NMR (126 MHz, $CDCl_3$) of **19**:

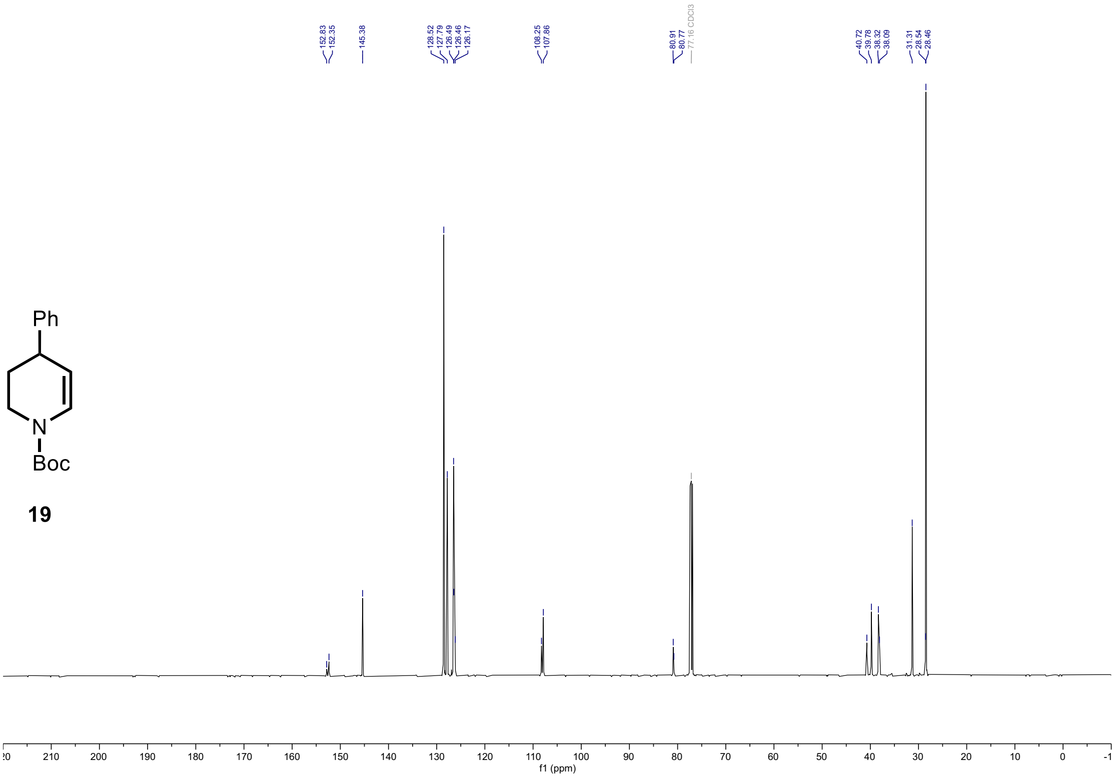

19

^{1}H NMR (500 MHz, $CDCl_3$) of **24**:

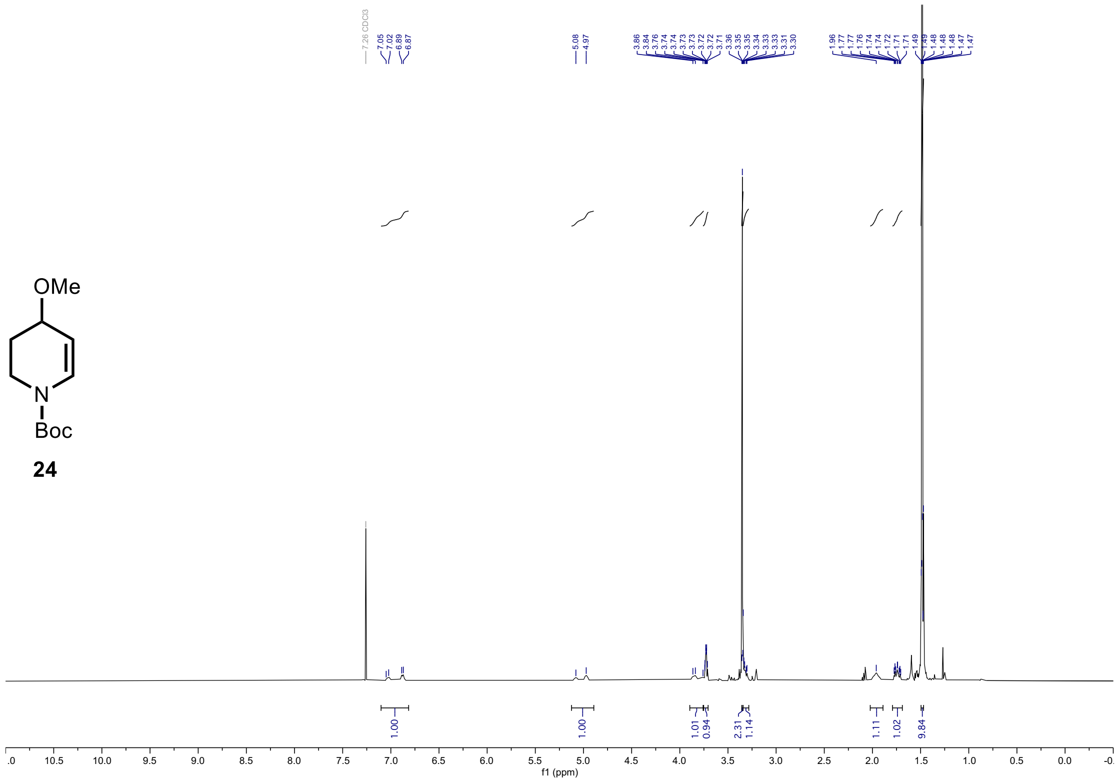

^{13}C NMR (126 MHz, $CDCl_3$) of **24**:

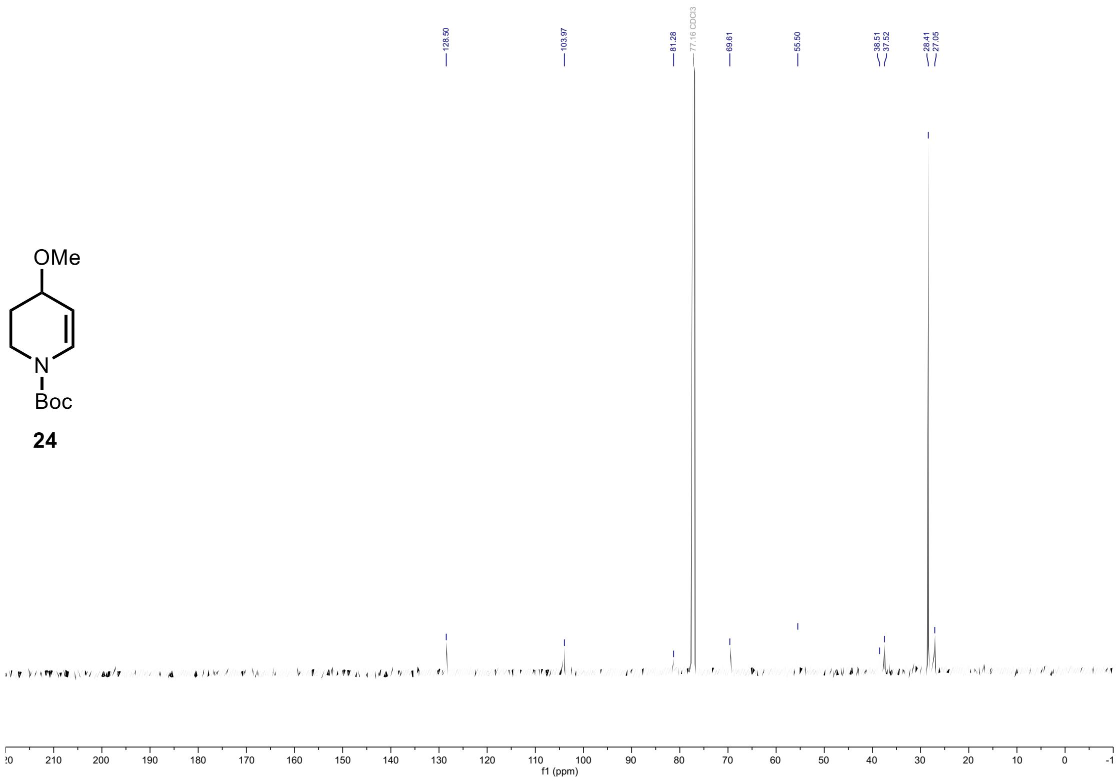

^{1}H NMR (500 MHz, $CDCl_3$) of **25**:

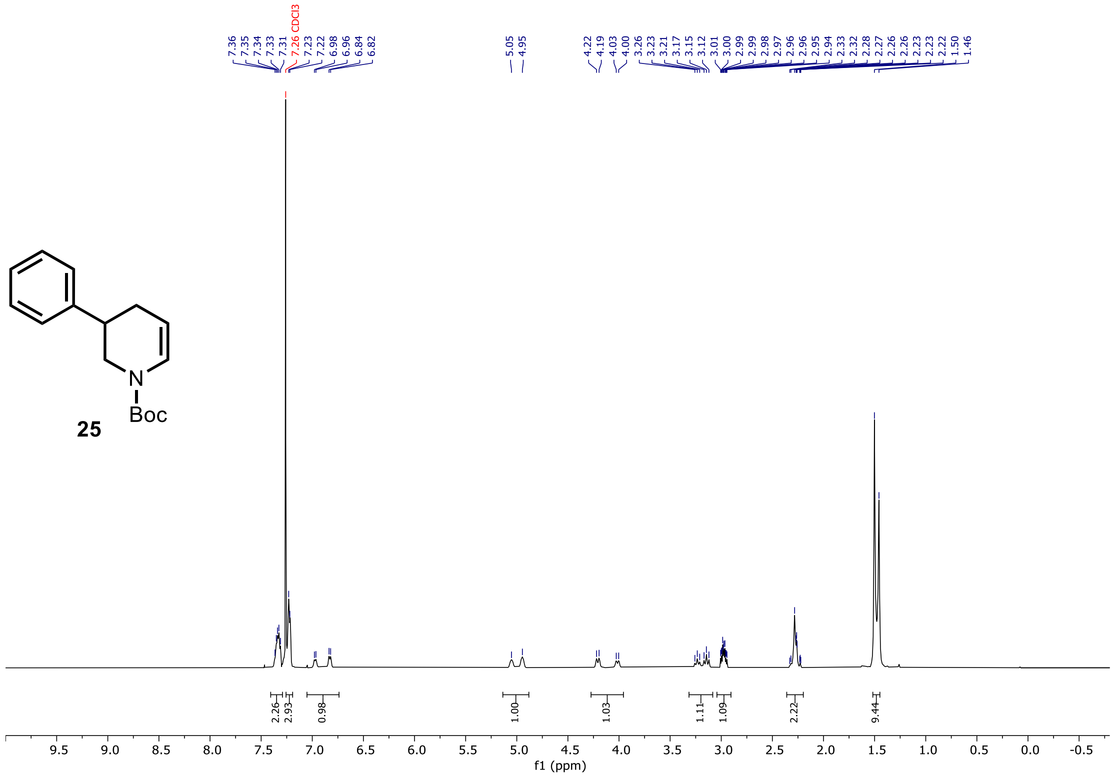

^{13}C NMR (126 MHz, $CDCl_3$) of **25**:

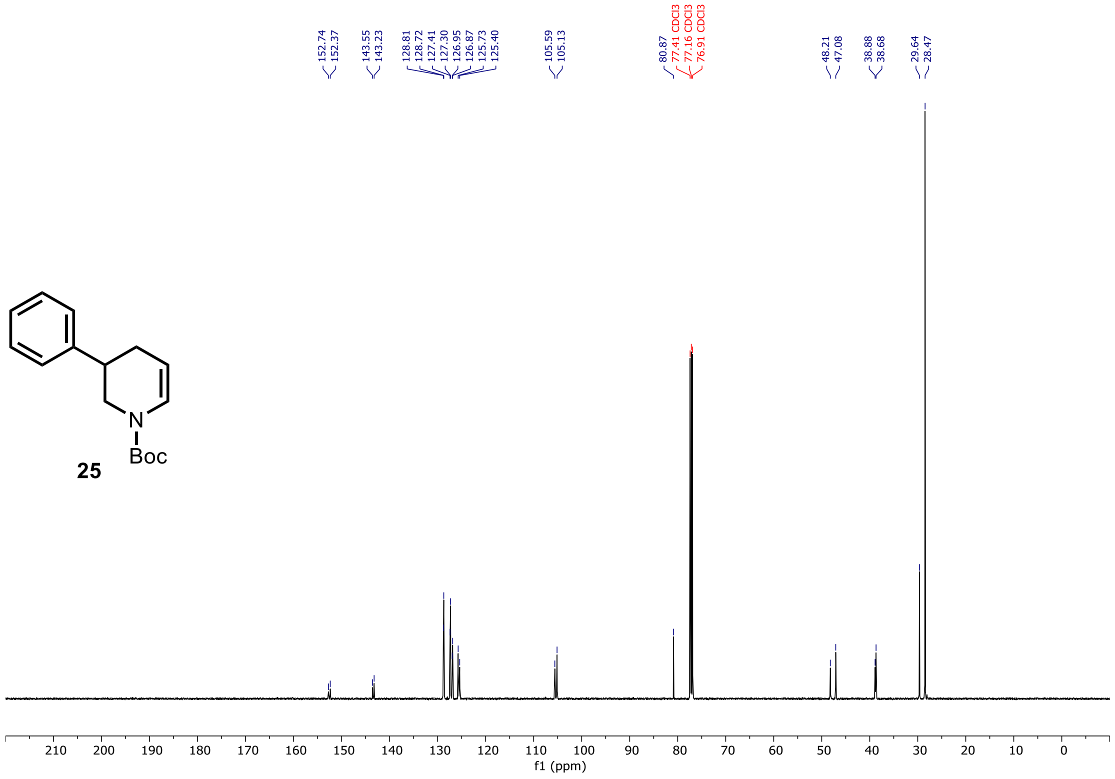

^{1}H NMR (500 MHz, $CDCl_3$) of **26**:

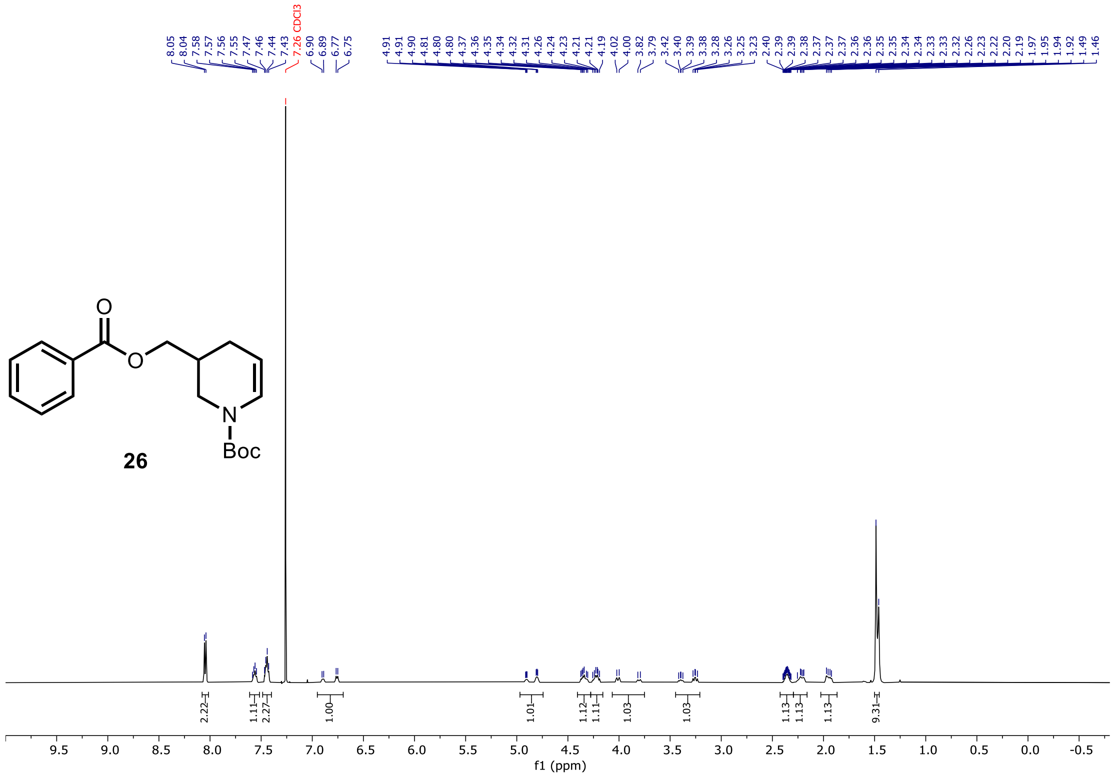

^{13}C NMR (126 MHz, $CDCl_3$) of **26**:

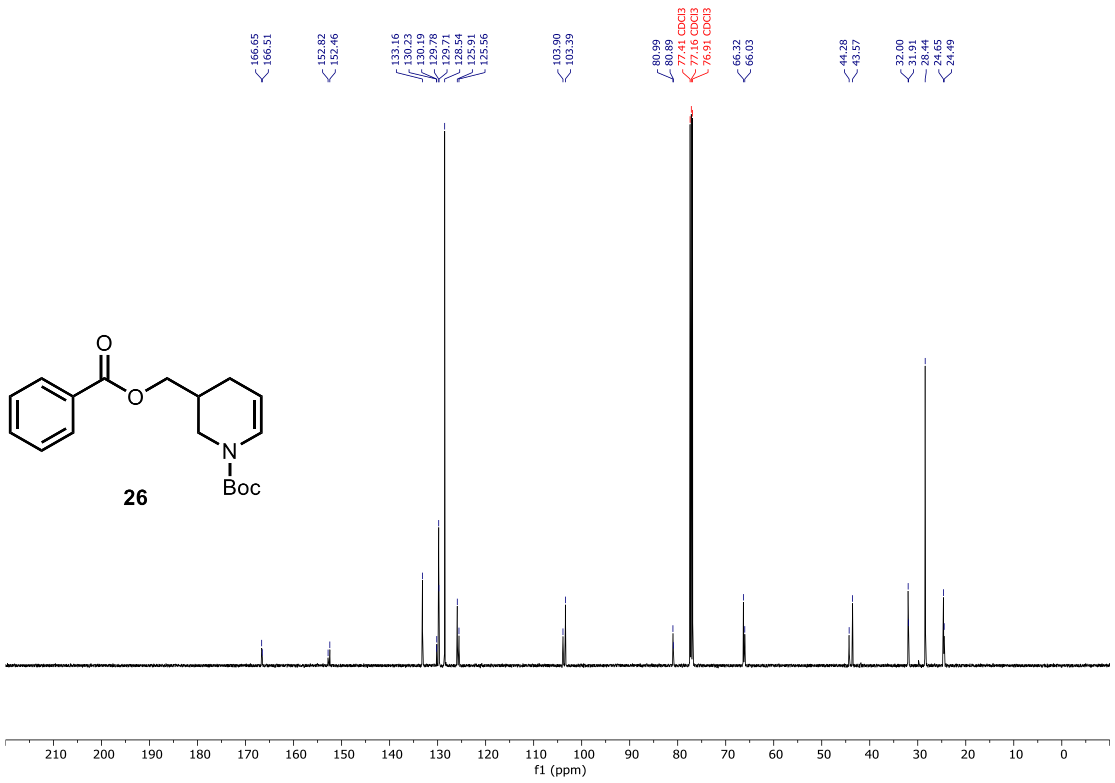

^{1}H NMR (500 MHz, $CDCl_3$) of **28**:

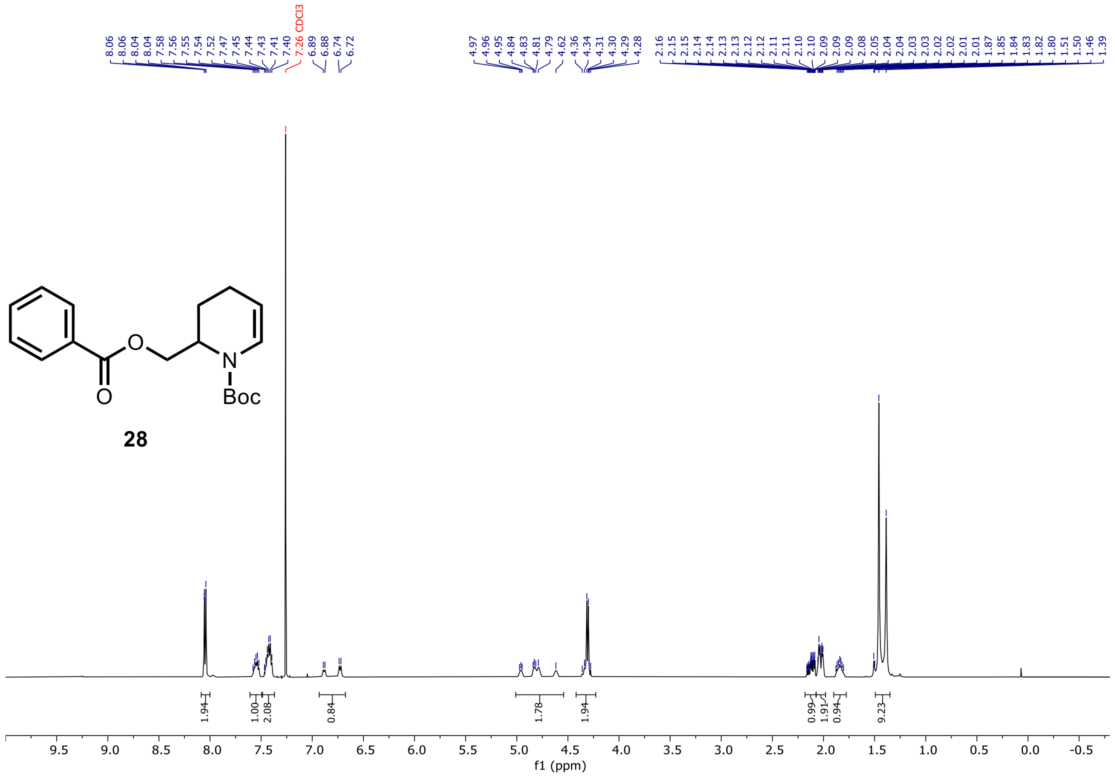

^{13}C NMR (126 MHz, $CDCl_3$) of **28**:

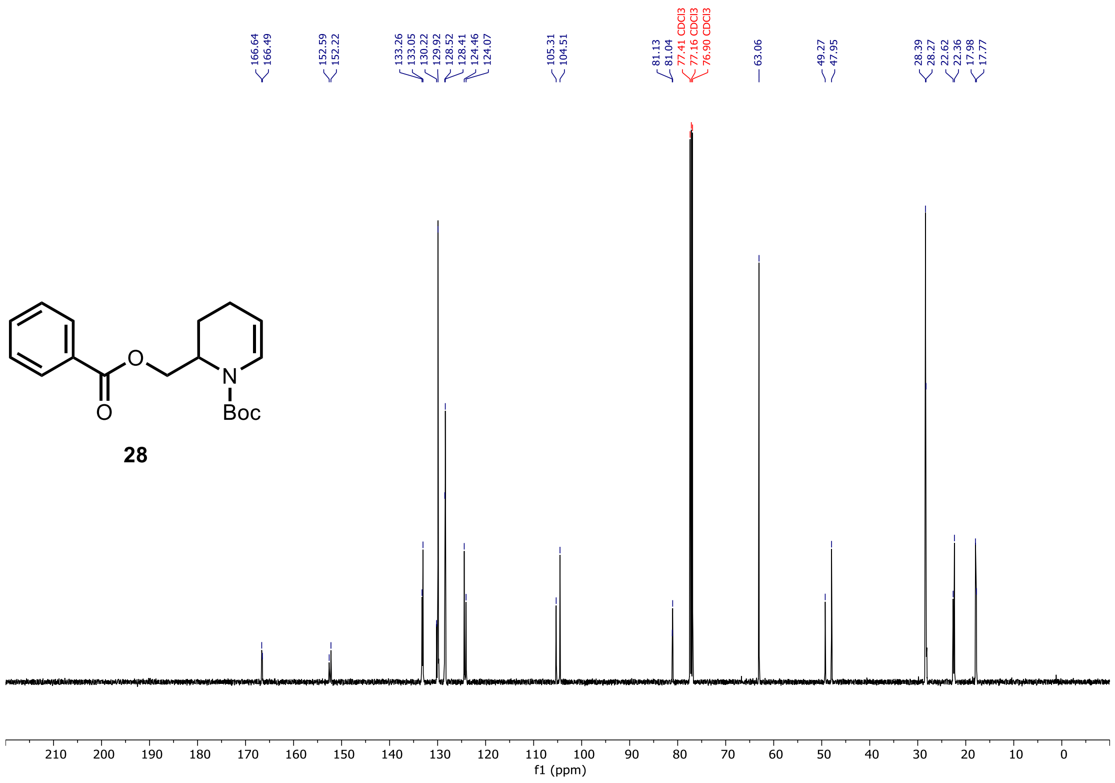

^{1}H NMR (500 MHz, $CDCl_3$) of **42**:

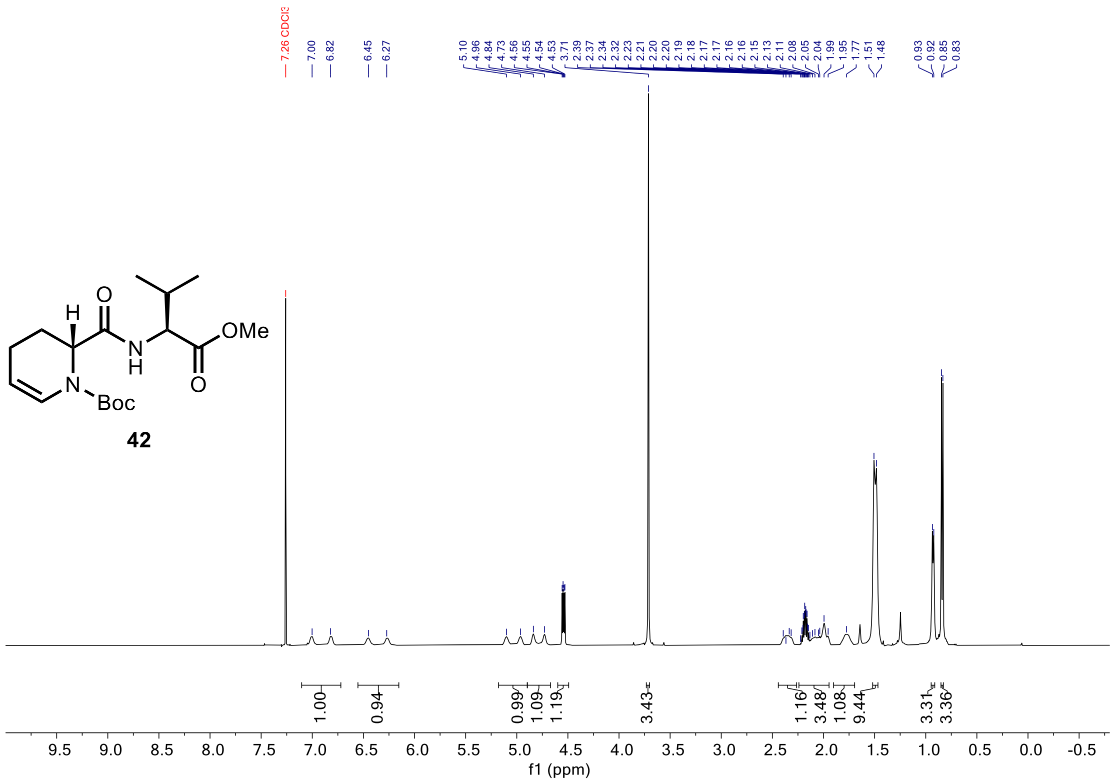

^{13}C NMR (126 MHz, $CDCl_3$) of **42**:

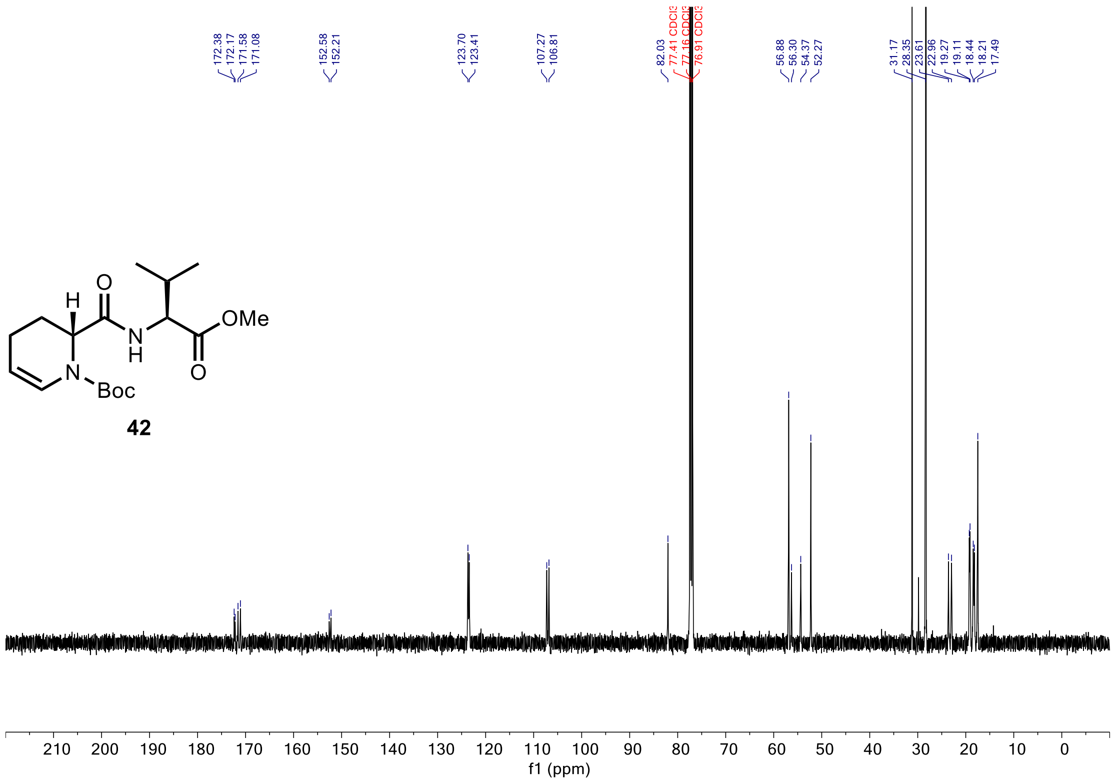

8. NMR Spectra of Derivatives of 1 and 2

8.1 Hemiaminal Derivatives

^{1}H NMR (400 MHz, $CDCl_3$) of **30**:

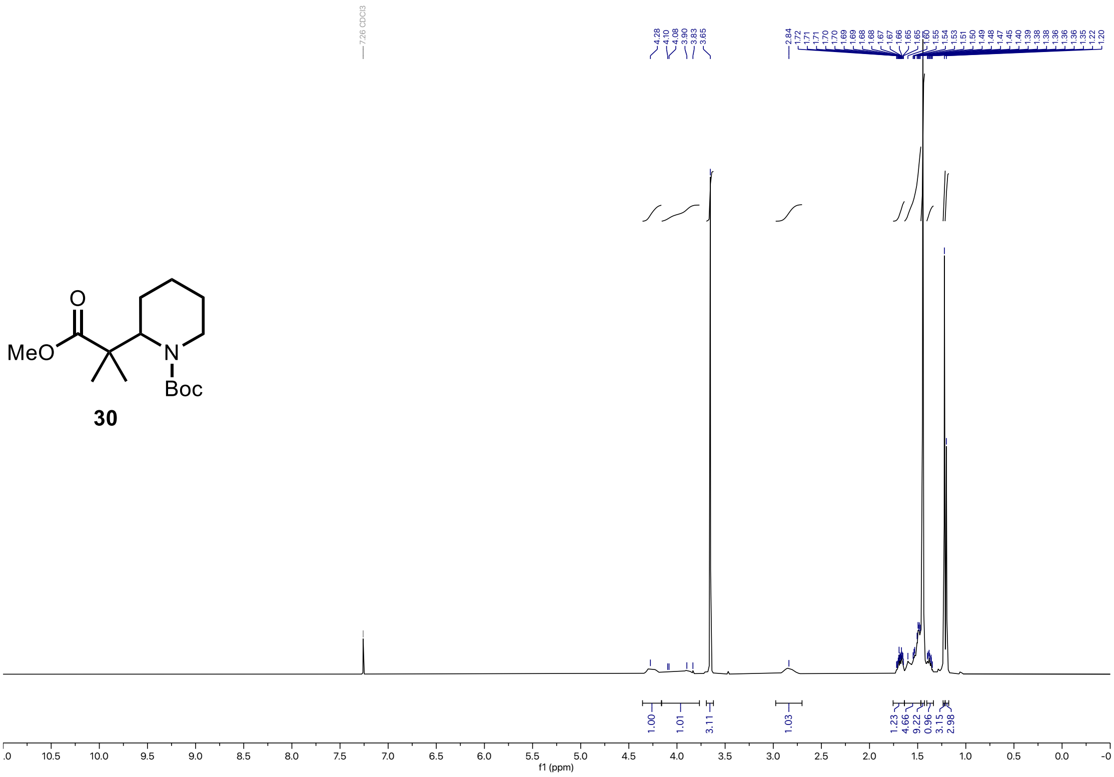

^{13}C NMR (101 MHz, $CDCl_3$) of **30**:

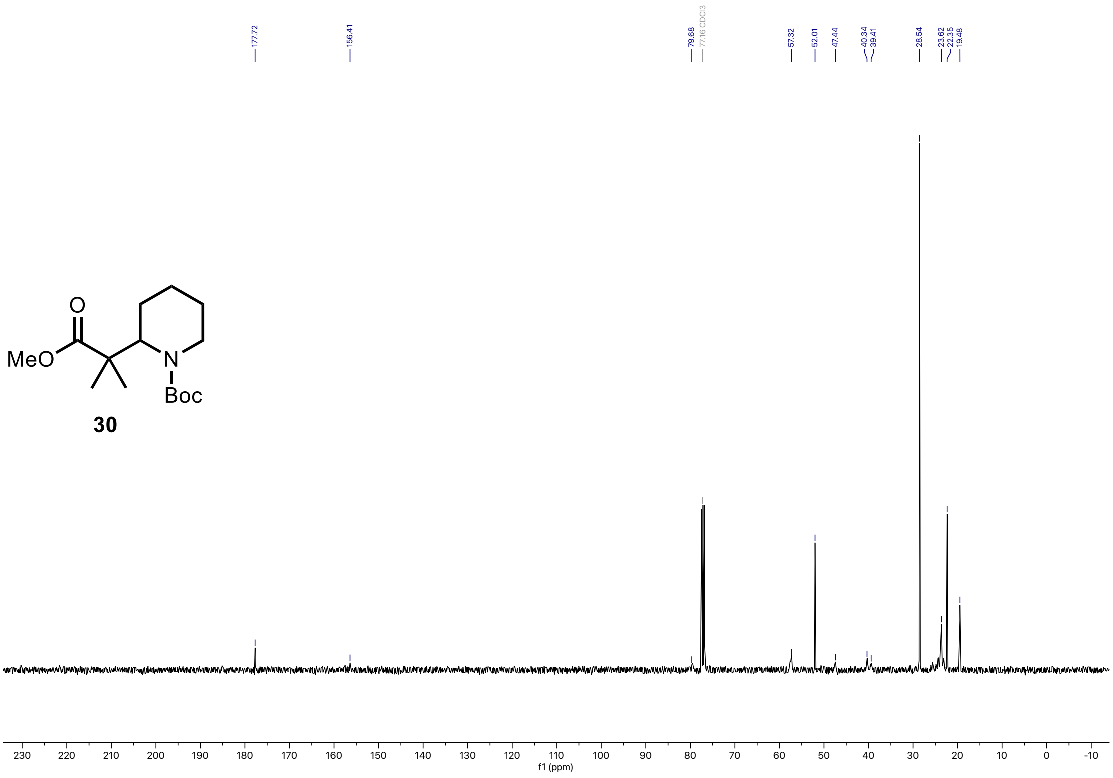

^{1}H NMR (500 MHz, $CDCl_3$) of **31**:

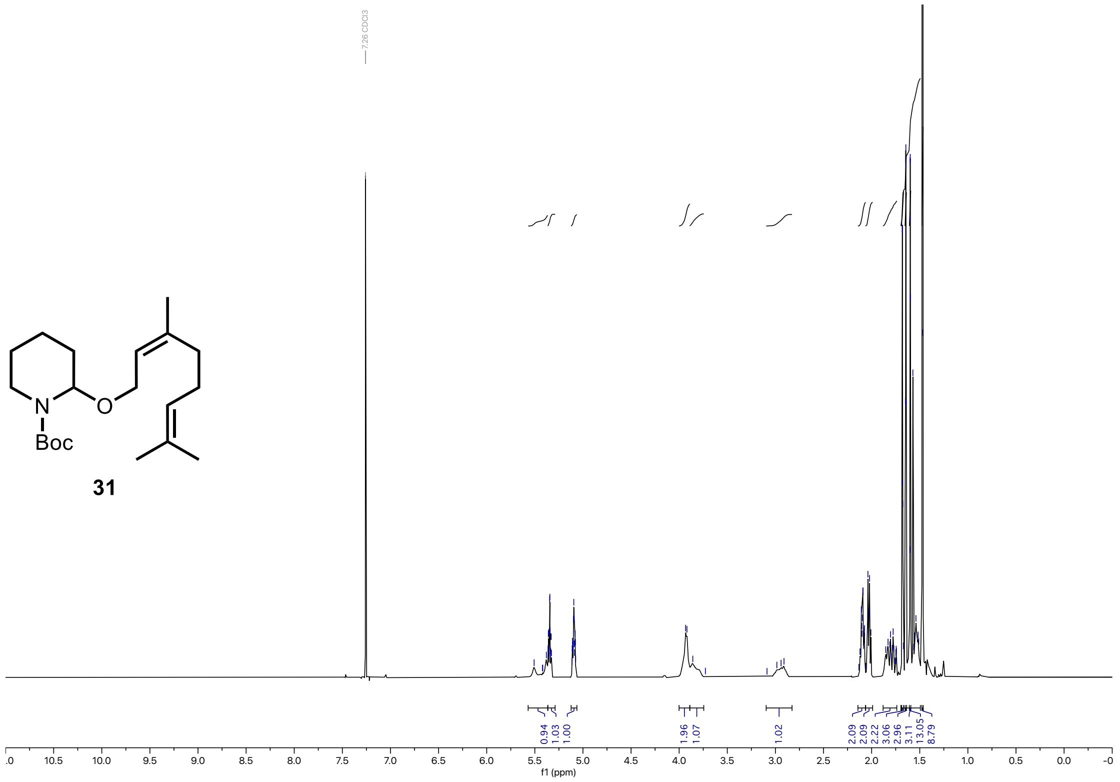

^{13}C NMR (126 MHz, $CDCl_3$) of **31**:

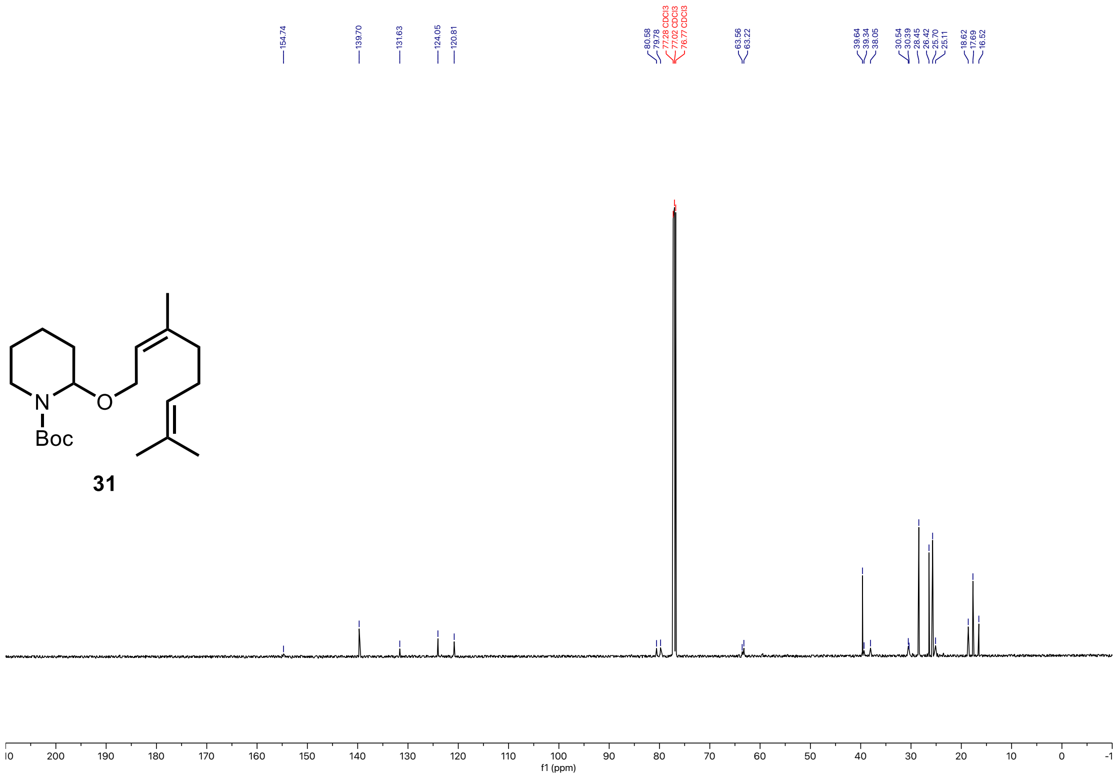

^{1}H NMR (500 MHz, $CDCl_3$) of **34** (mixture of diastereoisomers):

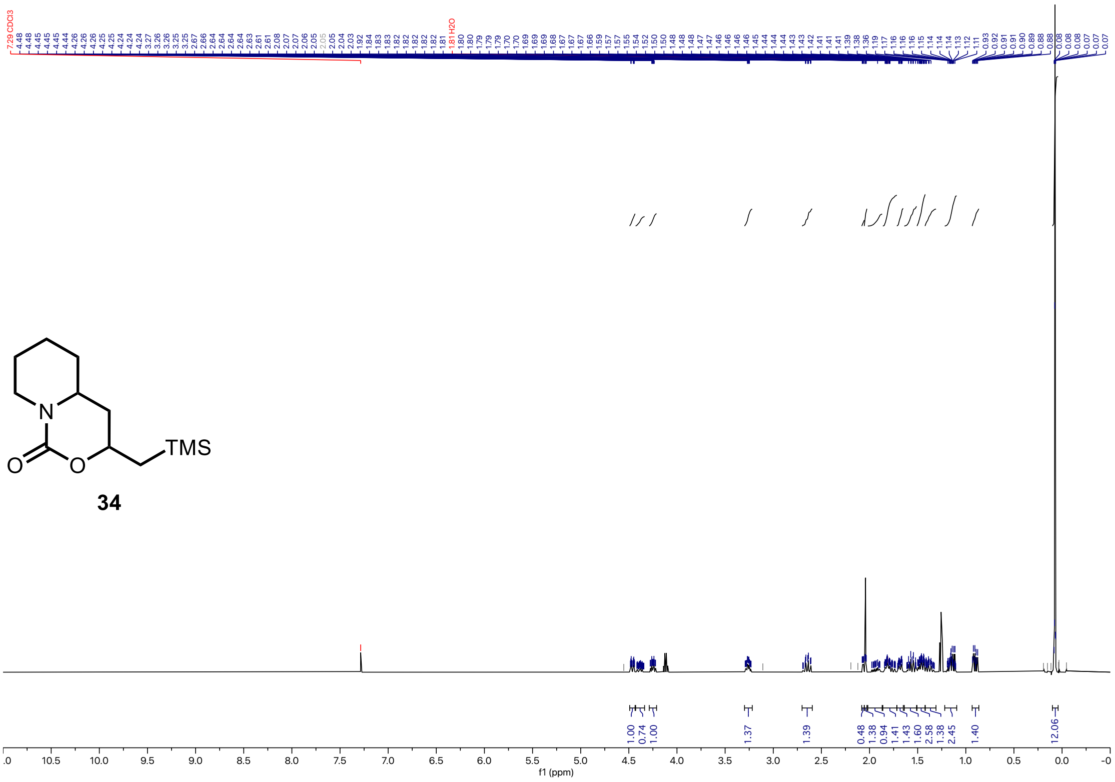

^{13}C NMR (126 MHz, $CDCl_3$) of **34**:

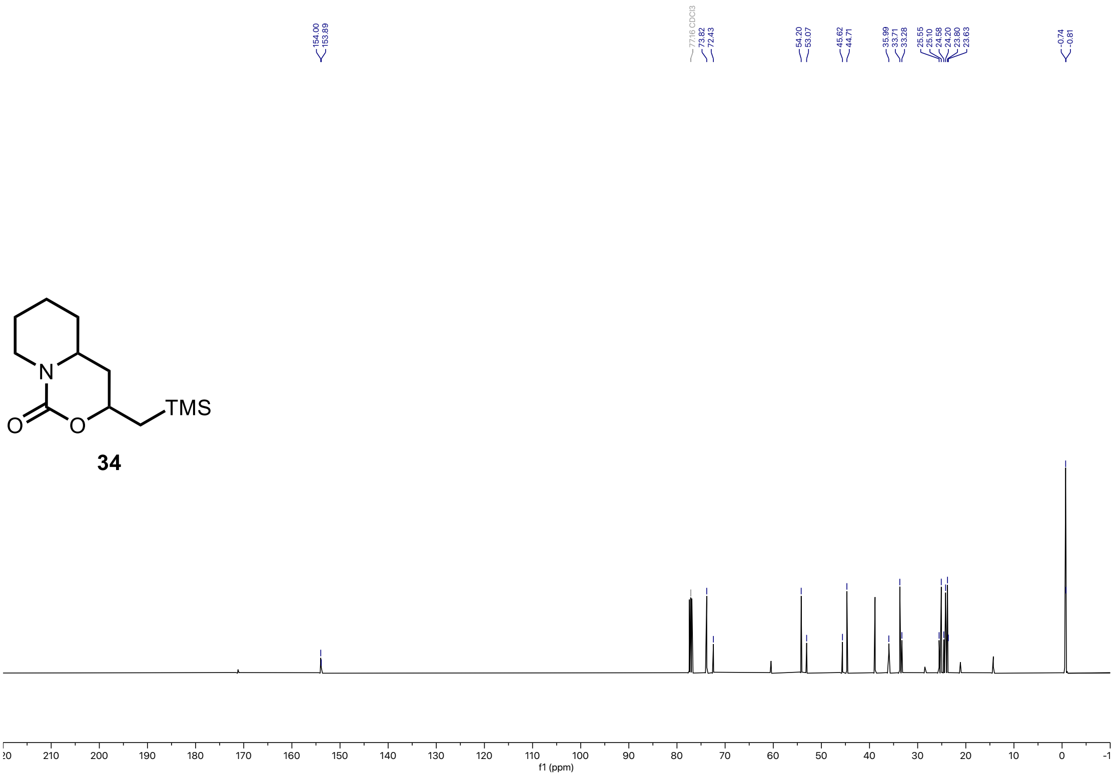

^{1}H NMR (500 MHz, $CDCl_3$) of **41** (relative configuration at piperidine *trans*):

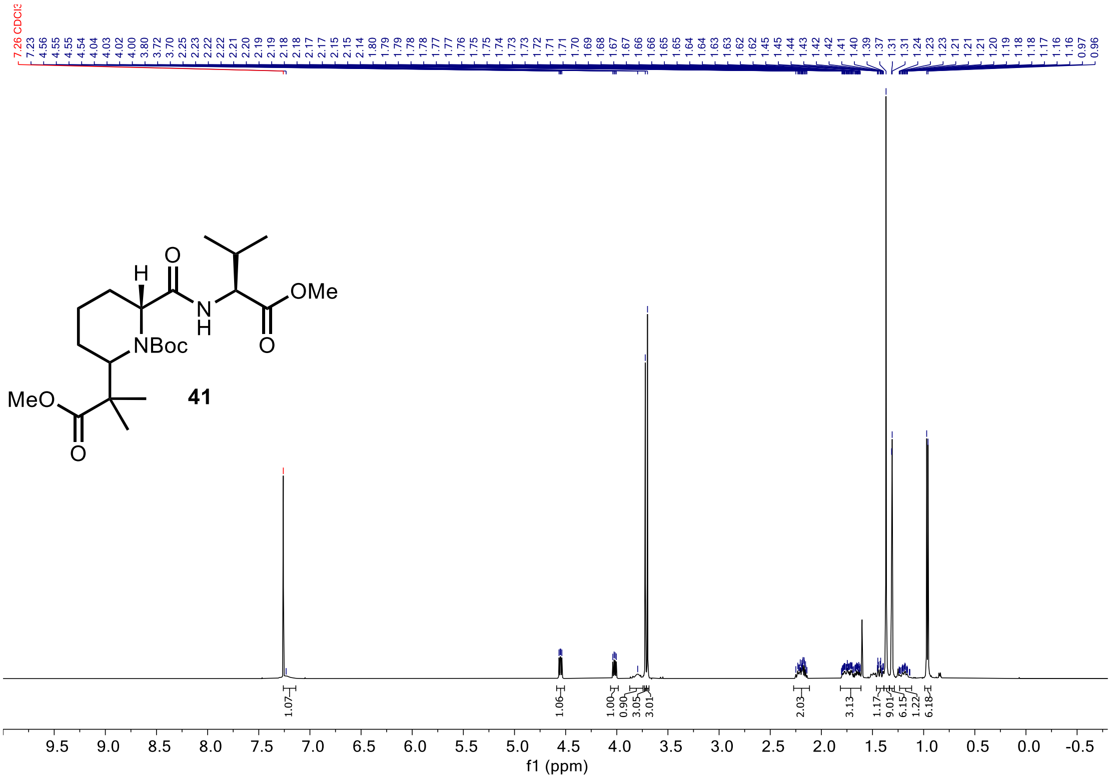

^{13}C NMR (126 MHz, $CDCl_3$) of **41**:

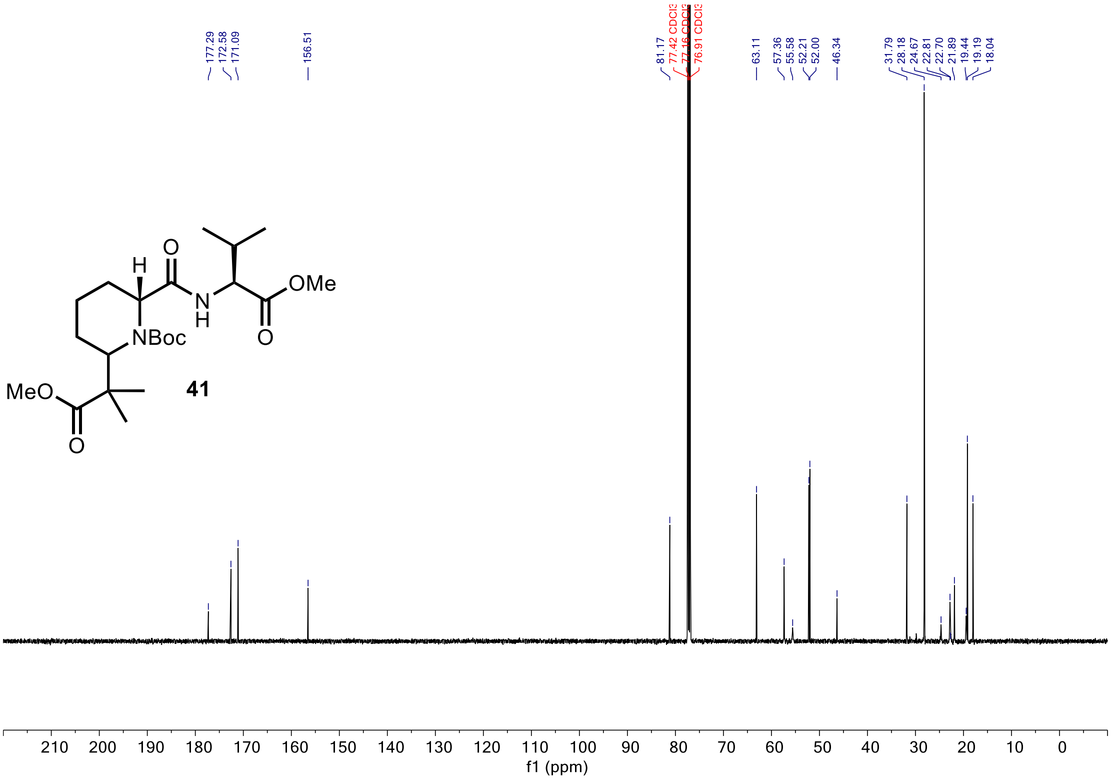

8.2 Enecarbamate Derivatives

^{1}H NMR (600 MHz, $CDCl_3$) of **43** (relative configuration at piperidine *trans*):

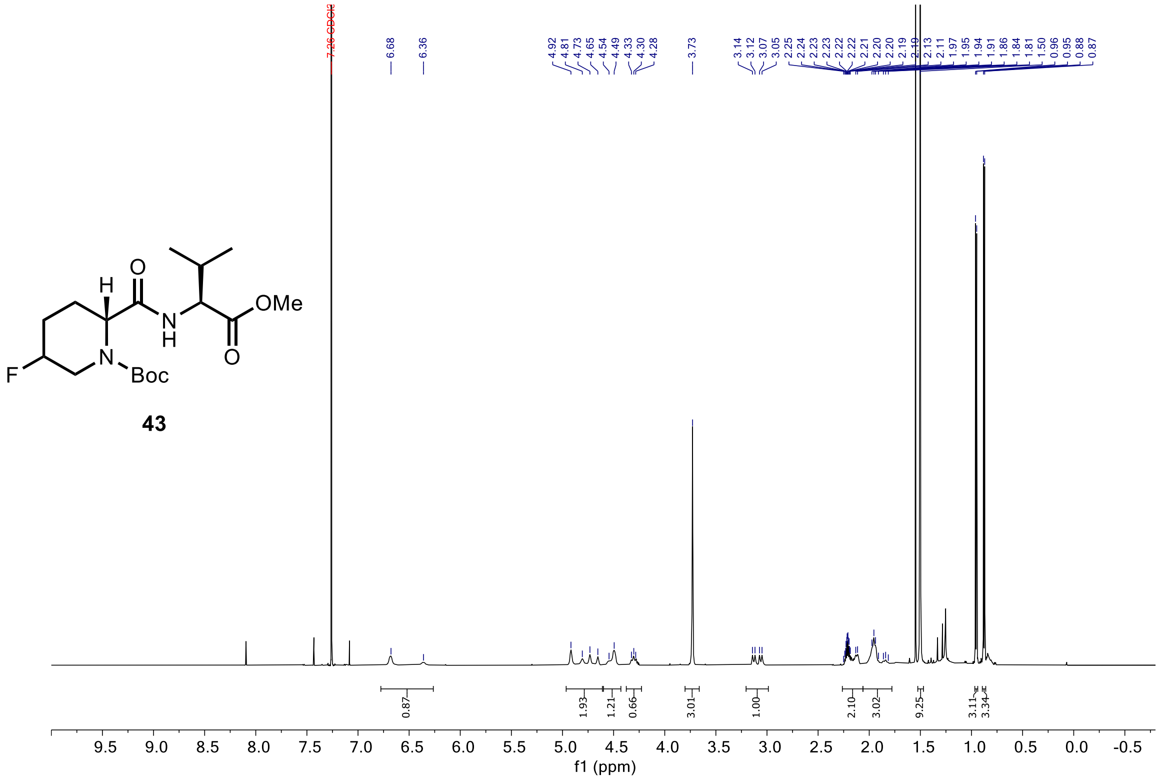

^{13}C NMR (151 MHz, $CDCl_3$) of **43**:

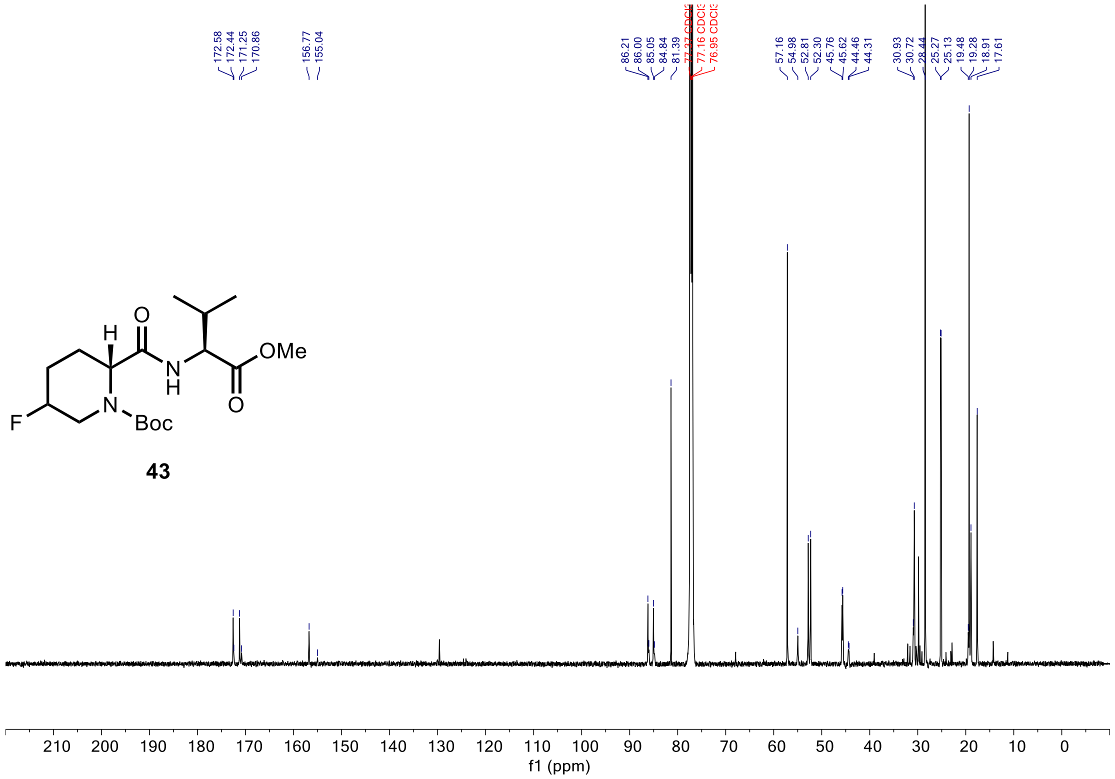

^{19}F NMR (471 MHz, $CDCl_3$) of **43**:

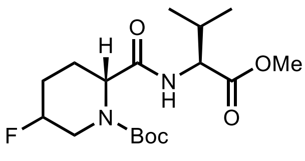

43

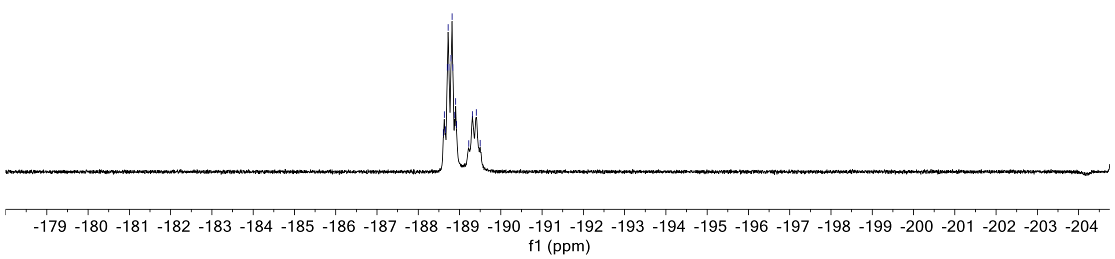

9. References

(1) Rackl, J. W.; Müller, A. F.; Bärtschi, C.; Wennemers, H. *ETHos* – A Swiss-Made Open-Source Modular Photoreactor for Laboratory-Scale Photochemical Reactions**.** *Helv. Chim. Acta* **2024**, 107, 12, e202400154.

(2) Metternich, J. B.; Sagebiel, S.; Lückener, A.; Lamping, S.; Ravoo, B. J.; Gilmour, R. Covalent Immobilization of (−)-Riboflavin on Polymer Functionalized Silica Particles: Application in the Photocatalytic E→Z Isomerization of Polarized Alkenes. *Chem. Eur. J.* **2018**, 24, 4228–4233.

(3) Schützenmeister, N.; Müller, M.; Reinscheid, U. M.; Griesinger, C.; Leonov, A. Trapped in Misbelief for Almost 40 Years: Selective Synthesis of the Four Stereoisomers of Mefloquine. *Chem. Eur. J.* **2013**, 19, 51, 17584 – 17588.

(4) Dieter, R. K.; Sharma, R. A Facile Preparation of Enecarbamates. *J. Org. Chem.* **1996**, 61, 12, 4180–4184.

(5) Gelis, C.; Heusler, A.; Nairoukh, Z.; Glorius, F. Catalytic Transfer Hydrogenation of Arenes and Heteroarenes. *Chem. Eur. J.* **2020**, 26, 14090–14094.

(6) Yin, C.; Zhang, R.; Pan, Y.; Gao, S.; Ding, X.; Bai, S.-T.; Lang, Q.; Zhang, X. PPM Ir-f-phamidol-Catalyzed Asymmetric Hydrogenation of γ-Amino Ketones Followed by Stereoselective Cyclization for Construction of Chiral 2-Aryl-pyrrolidine Pharmacophores. *J. Org. Chem.* **2024**, 89, 1, 527–533.

(7) Ritu; Das, S; Tian, Y-M.; Karl, T.; Jain, N.; König, B. Photocatalyzed Dehydrogenation of Aliphatic N-Heterocycles Releasing Dihydrogen. *ACS Catal*. **2022**, 12, 16, 10326-10332.

(8) Tereshchenko, O. D.; Perebiynis, M. Y.; Knysh, I. V.; Vasylets, O. V.; Sorochenko, A. A.; Slobodyanyuk, E. Y.; Rusanov, E. B.; Borysov, O. V.; Kolotilov, S. V.; Ryabukhin, S. V.; Volochnyuk, D. M. Electrochemical Scaled-up Synthesis of Cyclic Enecarbamates as Starting Materials for Medicinal Chemistry Relevant Building Bocks. *Adv. Synth. Catal.* **2020**, 362, 15, 3229 – 3242.

(9) Yu, X.; Zhao, H.; Li, P.; Koh, M. J. Iron-Catalyzed Tunable and Site-Selective Olefin Transposition. *J. Am. Chem. Soc.* **2020**, 142, 42, 18223–18230.

(10) Pizzuti, M. G.; Minnaard, A. J.; Feringa, B. L. Catalytic asymmetric synthesis of the alkaloid (+)-myrtine. *Org. Biomol. Chem.* **2008**, 6, 3464-3466.

(11) Petricci, E.; Mann, A.; Schoenfelder, A.; Rota, A.; Taddei, M. Microwaves Make Hydroformylation a Rapid and Easy Process. *Org. Lett.* **2006**, 8, 17, 3725–3727.

(12) de Godoy, L. A. F.; Camilo, N. S.; Pilli, R. A. Addition of carbon nucleophiles to cyclic *N*-acyliminium and oxocarbenium ions under solvent-free conditions. *Tetrahedron Lett.* **2006**, 47, 7853–7856.

(13) Shono, T.; Matsumura, Y.; Tsubata, K.; Takata, J. Tayama, E.; Otoyama, S.; Isaka, W. Brønsted acid catalyzed regioselectiveaza-Ferrier reaction: a novel synthetic method for α-(N-Boc-2-pyrrolidinyl) aldehydes. *Chem. Commun.* **2008**, 4216-4218

(14) One Step Synthesis of α-Aminoalkylfurans and its Application to a Facile Synthesis of Pyridoxine (Vitamin B6). *Chem. Lett.* **1981**, 1121-1124

(15) Lankelma, M.; Olivares, A. M.; de Bruin, B. [Co(TPP)]-Catalyzed Formation of Substituted Piperidines. *Chem. Eur. J.* **2019**, 25, 5658-5663.

(16) Ramirez, N. P.; König, B.; Gonzalez-Gomez, J. C. Decarboxylative Cyanation of Aliphatic Carboxylic Acids via Visible-Light Flavin Photocatalysis. *Org. Lett.* **2019**, 21, 5, 1368–1373.

(17) Novaes, L. F. T.; Ho, J. S. K.; Mao, K.; Villemure, E.; Terrett, J. A.; Lin, S. α,β-Desaturation and Formal β-C(sp3)–H Fluorination of *N*-Substituted Amines: A Late-Stage Functionalization Strategy Enabled by Electrochemistry. *J. Am. Chem. Soc.* **2024**, 146, 33, 22982-22992.

(18) Jerkovic, T.; Cruickshank, H.; Chen, Y.; Trindade, A. F.; Dumas, A. M.; Edwards, J.; Alorati, A.; Ho, H. E. Development and Kilogram-Scale Implementation of a Flavin-Catalyzed Photoredox Fluorodecarboxylation. *Org. Process Res. Dev.* **2024**, 28, 1, 266–272.

(19) Shono, T.; Matsumura, Y.; Tsubata, K.; Sugihara, Y.; Yamane, S.-I.; Kanazawa, T.; Aoki, T. Electroorganic Chemistry. 60. Electroorganic Synthesis of Enamides and Enecarbamates and Their Utilization in Organic Synthesis 1. *J. Am. Chem. Soc.* **1982**, 104, 6697-6703.

(20) Boyington, A. J.; Seath, C. P.; Zearfoss, A. M.; Xu, Z.; Jui, N. T. Catalytic Strategy for Regioselective Arylethylamine Synthesis. *J. Am. Chem. Soc.* **2019**, 141, 9, 4147–4153.

(21) Gonnard, L.; Guérinot, A.; Cossy, J. Cobalt-Catalyzed Cross-Coupling of 3- and 4-Iodopiperidines with Grignard Reagents. *Chem. Eur. J.* **2015**, 21, 12797–12803.

(22) Crysalispro, *1.171.43.143a*, **2024**, Rigaku OD.

(23) Sheldrick, G. M. SHELXT - integrated space-group and crystal-structure determination. *Acta Crystallogr A Found Adv.* **2015**, *71* Pt 1, 3-8.

(24) Sheldrick, G. M. Crystal structure refinement with SHELXL. *Acta Crystallogr C Struct Chem.* **2015**, *71* Pt 1, 3-8.

(25) Groom, C. R.; Bruno, I. J.; Lightfoot, M. P.; Ward, S. C. The Cambridge Structural Database. *Acta Crystallogr B Struct Sci Cryst Eng Mater.* **2016**, *72* Pt 2, 171-179.

(26) Kratzert, D. *FinalCif*, *V151*, https://dkratzert.de/finalcif.html.
